# Histone H3 lysine 4 acetylation and methylation dynamics define breast cancer subtypes

**DOI:** 10.18632/oncotarget.6922

**Published:** 2016-01-15

**Authors:** Terri L. Messier, Jonathan A. R. Gordon, Joseph R. Boyd, Coralee E. Tye, Gillian Browne, Janet L. Stein, Jane B. Lian, Gary S. Stein

**Affiliations:** ^1^ Department of Biochemistry and University of Vermont Cancer Center, University of Vermont College of Medicine, Burlington, VT, USA

**Keywords:** epigenetics, breast cancer, estrogen receptor pathway, EMT pathway, histone H3K4 modifications

## Abstract

The onset and progression of breast cancer are linked to genetic and epigenetic changes that alter the normal programming of cells. Epigenetic modifications of DNA and histones contribute to chromatin structure that result in the activation or repression of gene expression. Several epigenetic pathways have been shown to be highly deregulated in cancer cells. Targeting specific histone modifications represents a viable strategy to prevent oncogenic transformation, tumor growth or metastasis. Methylation of histone H3 lysine 4 has been extensively studied and shown to mark genes for expression; however this residue can also be acetylated and the specific function of this alteration is less well known. To define the relative roles of histone H3 methylation (H3K4me3) and acetylation (H3K4ac) in breast cancer, we determined genomic regions enriched for both marks in normal-like (MCF10A), transformed (MCF7) and metastatic (MDA-MB-231) cells using a genome-wide ChIP-Seq approach. Our data revealed a genome-wide gain of H3K4ac associated with both early and late breast cancer cell phenotypes, while gain of H3K4me3 was predominantly associated with late stage cancer cells. Enrichment of H3K4ac was over-represented at promoters of genes associated with cancer-related phenotypic traits, such as estrogen response and epithelial-to-mesenchymal transition pathways. Our findings highlight an important role for H3K4ac in predicting epigenetic changes associated with early stages of transformation. In addition, our data provide a valuable resource for understanding epigenetic signatures that correlate with known breast cancer-associated oncogenic pathways.

## INTRODUCTION

Epigenetic changes in chromatin structure and post-translational modification in response to developmental, environmental and hormonal stimuli, mediate changes in transcriptional output that ultimately define distinct cell phenotypes. A major component of epigenetic regulation is the post-translational modification of histone tails that affects chromatin structure by destabilizing histone interactions with DNA, altering nucleosome contacts, and forming binding sites for transcriptional regulators that influences gene expression. Under normal circumstances these shifts in histone modifications result in programmed phenotypic changes such as cell differentiation. However, there is increasing evidence that alteration of the histone epigenome is one of the earliest steps in oncogenic transformation [1, 2]. Although numerous histone post-translational modifications contribute to chromatin and gene regulation, the tri-methylation of histone 3 lysine 4 (H3K4me3) is a well-established marker of active or poised transcription [3] and enzyme complexes regulating this modification have been implicated in oncogenesis and cancer-related functions [4-6]. In contrast, the role of acetylation of H3K4 has been less studied. This is surprising given the importance of other acetylation marks, and given the implication of histone acetyltransferase and deacetylase enzymes in the progression of several types of tumors [7, 8]. In other cell systems, there is a coordinated interaction between H3K4me3 and H3K4ac modifications; however, it is unclear how these marks function independently to regulate gene expression and a detailed, global analysis of these modifications in cancer has yet to be directly evaluated [9-11]. In this study, we tested the hypothesis that the differential enrichment of these specific histone marks may define the cellular and molecular characteristics that are hallmarks of breast cancer cell phenotypes.

Reliable early detection and prevention of cancer depend on a better understanding of the initial molecular events involved in driving mammary epithelial cells toward a transformed state. Although there has been a decline in breast cancer incidence in recent years, it remains the second leading cause of death among women in the US with an estimated 234,000 new cases of invasive breast cancer and 60,000 new cases of carcinoma in situ diagnosed in 2015 [12]. The most common classification system for breast cancer is presently based on histological type, tumor grade, lymph node status and the presence of predictive markers such as estrogen receptor alpha (ESR1), progesterone receptor (PGR) and epidermal growth factor receptor (HER2). More recently, molecular profiling has been used to categorize breast cancers into multiple subtypes with different prognoses and treatment responses [13, 14]. The triple negative basal-like subtype is the most difficult to treat due to an absence of therapeutic targets. Basal-like breast cancer cell lines often lose their epithelial characteristics and undergo epithelial-to-mesenchymal transition (EMT) with characteristic gene expression changes [15]. The mesenchymal phenotype in breast cancer cells has been linked with increased invasion, metastasis, and the acquisition of stem cell characteristics [16-18]. In this study, three well-established human mammary cell lines were chosen to represent a normal-like subtype (MCF10A; fibrocystic disease) and two cancer subtypes, luminal (MCF7; ESR1/PGR+) and basal-like metastatic (MDA-MB-231; ESR1/PGR- HER2-); together these provide a relatively comprehensive model to recapitulate the transitional differentiation observed in breast cancer subtypes, as it is difficult to mirror the heterogeneity associated with clinical samples with a single cell line [13, 14, 19]. By establishing changes in H3K4me3 and H3K4ac in these three model cell lines, we will be able to define an epigenetic signature of distinct stages of cancer and determine molecular differences leading to phenotypic changes during cancer progression.

Here we provide a global map of H3K4me3 and H3K4ac in breast cancer. Our analysis defines a pattern of H3K4 enrichment that correlates with breast cancer cell subtype, estrogen response and the EMT oncogenic pathway. These discoveries highlight a specific role for H3K4ac as an epigenetic hallmark of cellular transformation, provide a better overall understanding of epigenetic changes leading to cancer, and may identify new biomarkers for breast cancer surveillance and pharmacologic targets for treatment.

## RESULTS

### Specificity of histone H3K4 tri-methylation and acetylation patterns in breast cancer cell subtypes

**Figure 1:**
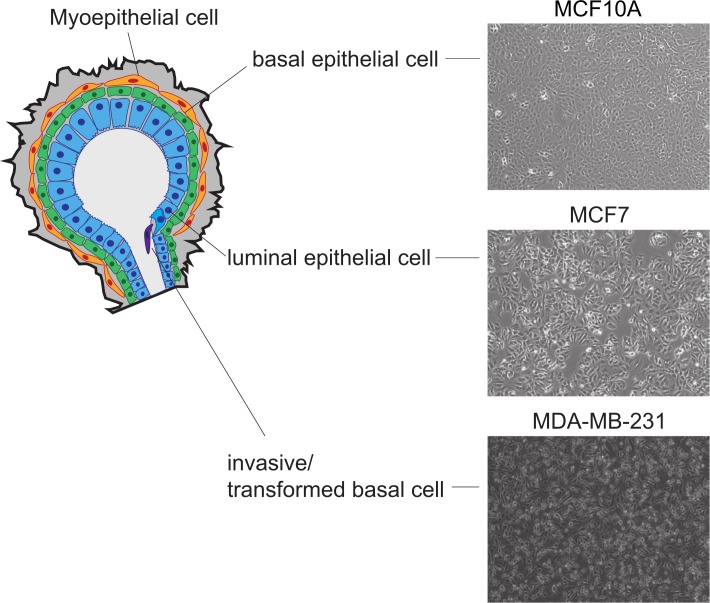
Characteristics of cell lines used to model the diverse heterogeneity observed in breast cancer Schematic model of breast cancer cell lines used to assay histone modification changes. MCF10A represents a normal basal epithelial, ESR/PGR low-negative, MCF7 represents the luminal cell subtype, ESR1+/ESR2+/PGR+, correlative with a more favorable prognosis, and MDA-MB-231 is representative of the metastatic basal cell subtype, ESR1-/PGR-/Her2- correlative with a less favorable prognosis.

As a model of distinct stages of breast cancer progression, we selected three well-characterized cell lines (Figure 1). MCF10A is a non-cancerous cell line showing characteristics of epithelial hyperplasia and falls within the normal-basal subtype exhibiting minimal to no expression of estrogen or progesterone steroid receptors. The MCF7 cell line represents the mature luminal subtype of breast cancer expressing both the estrogen and progesterone receptors. This subtype (luminal, ESR1+, PGR+) represents 40-75% of newly diagnosed breast cancers and responds well to hormone therapy. The MDA-MB-231 cell line is an invasive metastatic adenocarcinoma that is ESR1-, PGR-, HER2- (triple negative). This invasive, basal subtype represents 10-20% of breast cancers and has a poor short-term prognosis primarily due to a lack of targeted therapies. The identity of each cell line was verified by short tandem repeat (STR) DNA fingerprinting and characterized by q-PCR for expression of key regulatory markers attributed to each cell type (data not shown).

**Figure 2:**
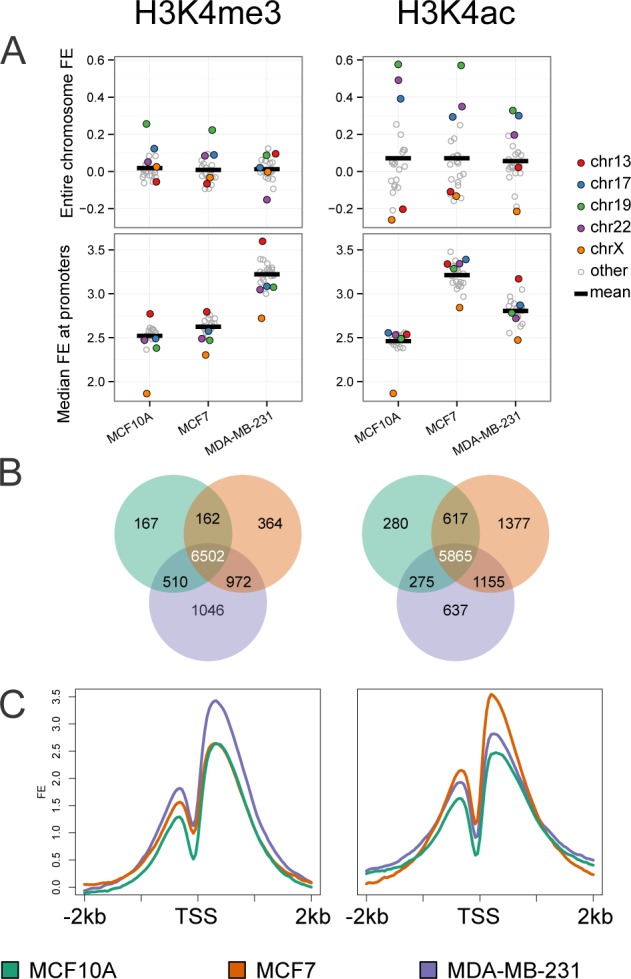
Genome-wide analysis of histone H3K4me3 and H3K4ac patterns by cell line **A.** Normalized read counts for H3K4me3 (left) and H3K4ac (right) were calculated for each chromosome and expressed as log_2_ FE (upper panels) and median FE at promoters of each chromosome (lower panel) for the three cell lines. Chromosomes 13,17,19,22, and X (highlighted in color) fall furthest from the mean (thick black line). **B.** Gene promoters (+/− 1kb from TSS) with significant H3K4me3 or H3K4ac enrichment were determined by calculating relative enrichment over input (> log_2_2 fold) and compared across the three cell lines (MCF10A: green, MCF7: orange, and MDA-MB-231: purple). 9,723 genes are marked by H3K4me3 in at least one cell line; likewise 10,206 are marked by H3K4ac. **C.** FE signal within +/− 2kb of TSS was plotted for each cell line. An increase in H3K4me3 enrichment was observed in the MDA-MB-231 cell line (left panel, purple line) and increased for H3K4ac enrichment in the MCF7 cell line (right panel, orange line).

One of the most extensively studied modifications of H3K4 is tri-methylation (H3K4me3), which has been shown to be highly regulated and tightly associated with active gene transcription. H3K4 acetylation (H3K4ac) is less well studied, but is reported to be associated with both promoter and transcribed regions of active genes [20, 21]. We also observed that a large percentage (greater than 70%) of the H3K4me3 and H3K4ac peaks were associated with promoters and gene bodies (Supplementary Figure S1). To address the contribution of these activating marks in relation to each cancer cell subtype as a model of cancer progression, we examined the signature of these histone modifications in each cell line. Using ChIP-seq analysis we assembled a genome wide map of H3K4me3 and H3K4ac patterns. Initially we characterized the distribution of these marks at the chromosome level (Figure 2A). The mean enrichment of H3K4me3 appeared relatively constant among the three cells lines, with the exception of elevated enrichment of chromosome 19 in MCF10A and MCF7 cell lines, and decreased enrichment in chromosome 22 in the metastatic MDA-231 cell line. Patterns for H3K4ac across all chromosomes showed the most dynamic change in MCF10A and MCF7 while MDA-MB-231 had a relatively uniform pattern of distribution across chromosomes. Chromosome 19 demonstrated the highest levels of H3K4ac in all cell lines. However, H3K4ac enrichment was markedly higher in MCF10A and MCF7 relative to the MDA-MB-231 cell line. This observation is consistent with the high gene density of chromosome 19 and suggests a focused role of this modification in regulation of genes in the MCF10A and MCF7 cell lines. Additionally, H3K4ac levels were increased on chromosomes 17 and 22 in the normal-like MCF10A cell line compared to MCF7 and MDA-MB-231.

Although we noted distinct changes at the whole chromosome level we sought to define changes in H3K4me3 and H3K4ac modifications at gene promoters (+/− 1Kb from the TSS). The relative enrichment of both marks showed little variation among chromosomes within cell lines when measured at promoters (Figure 2A, lower panel). However, overall changes in H3K4 epigenetic marks are increased at gene promoters in the two breast cancer cell lines as compared to the normal-like MCF10A cell line. Notably, H3K4me3 was highest in MDA-MB-231 (mean fold-enrichment (FE) of 9.3 for MDA-MB-231 compared to 6.1 for MCF7 and 5.7 for MCF10A); while H3K4ac at promoters was higher in both cancer cell lines MCF7 and MDA-MB-231 (mean FE of 9.2 and 7.0, respectively, compared to 5.5 for MCF10A). These trends suggest that increased H3K4ac may be an early cancer status epigenetic change, while H3K4me3 may reflect a late stage epigenetic mark. In addition, we noted a decrease in both H3K4 modifications on the X- chromosome for MCF10A, as expected due to inactive X promoters while in both breast cancer cell lines the chromosome X promoters are more highly marked by both H3K4me3 and H3K4ac. These results suggest a degree of gene reactivation consistent with reports of epigenetic instability of the inactive X in breast cancer [22].

We further narrowed our analysis to define enrichment of H3K4 marks at 10,964 protein coding gene promoters with a 4 fold or greater enrichment (FE) over input for at least one histone mark in at least one cell line (Figure 2B-2C). Similar to our chromosome-wide analysis, the subset of protein-coding promoters show the same increased enrichment of H3K4me3 in MDA-MB-231 when compared to the other two cell lines, while an increased enrichment of H3K4ac was observed in both the MCF7 and MDA-MB-231 cancer cell lines (Figure 2C).

To provide insight into the unique and overlapping marked gene promoters among the three cell lines, we constructed Venn diagrams using a threshold of 4 FE; 2 in log_2_ over input (Figure 2B). There are a large number of promoters in all three cell lines marked by either H3K4 tri-methylation (6502, 65%) or acetylation (5865, 58%). However, a distinct pattern of promoters that are more highly marked by either tri-methylation (range of 2-10%) or acetylation (range of 3-14%) among the three cell lines was also observed. Overall, the two cancer cell lines have the highest number of cell line specific marks for both H3K4me3 and H3K4ac. MDA-MB-231 showed the highest number (1046) of H3K4me3 marked promoters while the highest number (1377) for H3K4ac was observed in the MCF7 cells.

**Figure 3:**
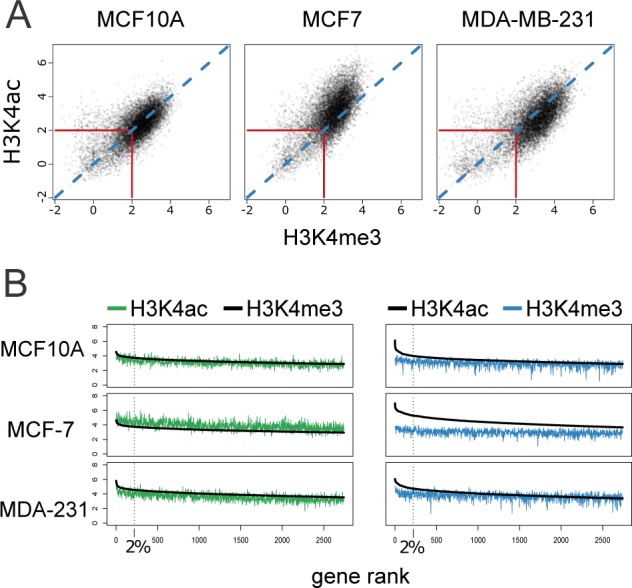
Correlation of H3K4me3 and H3K4ac enrichment at gene promoters among cancer-related cell lines **A.** Scatter plot of FE of H3K4me3 and H3K4ac at gene promoters. A strong correlation of the two marks was observed in the three cell lines with a large number of points clustered around the diagonal (blue dotted line) exceeding 4 fold (log_2_2) enrichment over input. **B.** The top 25% of H3K4me3 (left panel) or H3K4ac (right panel) enriched promoters were ranked by decreasing FE, log_2_2. Corresponding mark is depicted (H3K4ac in green and H3K4me3 in blue) using the identical rank order (with smoothing). Black dotted line denotes the top 2% of marked gene promoters. There is a greater divergence of acetylation enrichment from methylation enrichment in the highest ranked acetylated genes.

After evaluating the distribution of the two histone modifications among the three cell lines, we next addressed the correlation of the two marks within each cell line to reveal either tight association of the two marks or distinct patterns of each among the three cell lines. Using the same FE threshold as for the Venn diagrams, we found (10,964 promoters) that are marked by H3K4me3 and/or H3K4ac in any of the three cell lines. The scatterplots shown in Figure 3A reveal a strong correlation of H3K4 tri-methylation and acetylation within each cell line. In consideration of differences in antibody efficiency, we compared distribution patterns of the two cancer cell lines to the normal-like MCF10A cell line. MCF7 exhibits a striking shift toward higher H3K4ac compared to MCF10A. In contrast the MDA-MB-231 cell line has a more dispersed pattern with a trend toward H3K4me3.

To further interrogate the observed shifts in the correlative pattern of the two marks, we ranked the top quartile of gene promoters (2741) based on the highest fold enrichment for either H3K4me3 (Figure 3B, left panel) or H3K4ac (Figure 3B, right panel) and plotted the mean signal versus the corresponding mark mean signal (H3K4ac or H3K4me3, respectively) in each cell line. Of interest, the mean acetylation signal in MCF7 was higher than the signal for H3K4me3 in the top quartile of H3K4me3 gene promoters. In contrast, in MDA-MB-231 H3K4ac was lower than H3K4me3 (Figure 3B, left panel). A striking difference was observed in the top 2% (220 genes) of H3K4ac gene promoters as compared to H4K4me3 in all three cell lines. In the MCF7 cell line this difference extended beyond the top 2% (Figure 3B, right panel).

To address the biological pathway or processes associated with H3K4 acetylated gene promoters, we subjected the top 2% from the ranked list to Gene Ontology (GO) analysis. GO terms were related to regulation of metabolic process, regulation of gene expression, nucleic acid metabolism, cellular macromolecular metabolic process, and chromatin organization (Supplementary Table S3). Although our ChIP-seq data sets for modifications at histone H3K4 produced the interesting observation of a subset of highly H3K4 acetylated promoters, there were no indications of specific pathways (*e.g.*, tumor suppression, estrogen signaling, metastasis) that would define phenotypes ascribed to the individual cell lines.

### Epigenetic modifications correlate with gene set enrichment defined datasets

**Figure 4:**
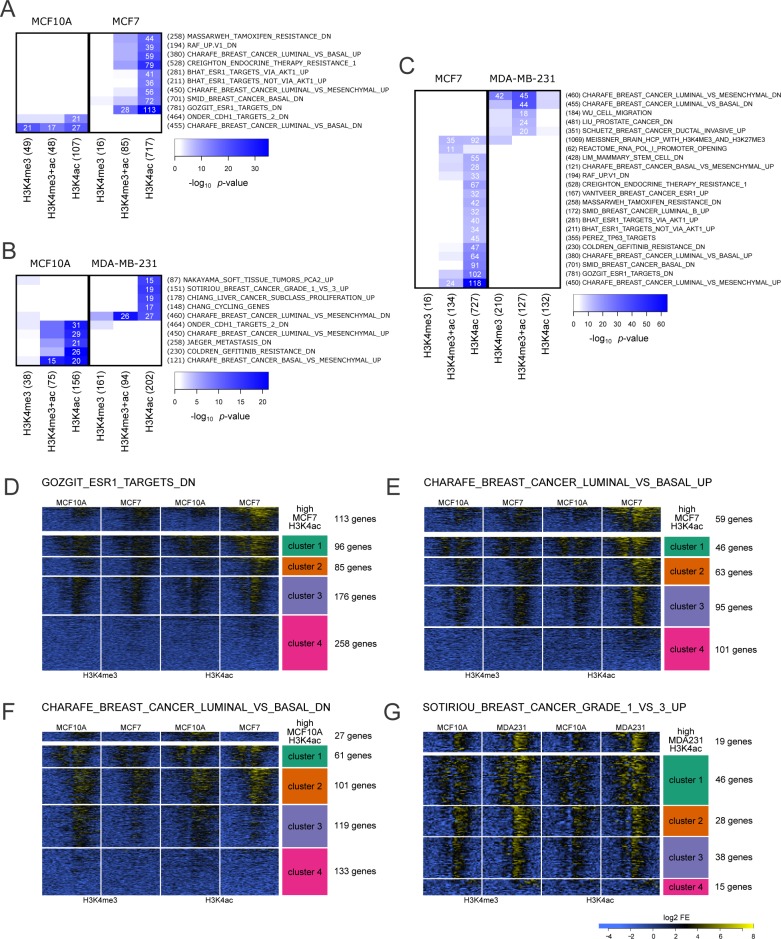
Cell line specific over-representation of marked gene promoters in MSigDB gene sets **A.**-**C.** Cell line comparisons showing the level of over-representation for gene promoters marked by H3K4me3 and/or H3K4ac (columns) within MSigDB gene sets (rows) with -log10 p-value indicated by color intensity. The number of marked genes and the size of each gene set are indicated in parentheses and the number of genes intersecting for combinations meeting significance threshold is noted in white. Each cancer cell line was compared to the normal MCF10A as shown in panels **A.** (MCF10A to MCF7) and **B**. (MCF10A to MDA-MB-231). **C**. Comparison of the two cancer cell lines, MCF7 and MDA-MB-231. K-means clustered heatmaps of enrichment profiles for gene promoters (+/− 2kb of TSS) from selected MSigDB datasets with highest (> 4 fold) differentially marked genes in each set were plotted separately. Clear patterns of differential localization and intensity of each mark were discovered in each MSigDB list. Analysis of selected lists from comparisons of MCF10A to MCF7, **D**.-**F**., and MCF10A to MDA-MB-231, **G**., are depicted.

To explore the biological significance of the dynamic changes in H3K4 modifications we used our data sets to interrogate gene expression lists to determine whether our enriched gene promoter subsets were associated with any defined biological processes or pathways within the Molecular Signatures Database (MSigDB). These analyses provided information from our histone modification signatures with specific *a priori* defined pathways associated with cancer (Figure 4). Our initial focus on the subset of gene promoters with at least a 4 fold change in enrichment between cell lines linked phenotypic properties of the three cell lines to defined curated gene sets.

Comparison of MCF10A to MCF7 at H3K4me3 and H3K4ac enriched gene promoters correlated with MSigDB gene expression sets from cancer-related curated gene sets (C2) and oncogenic signatures (C6). The dynamic histone changes between these two cell lines correlated with gene lists associated with endocrine response pathways, endocrine therapy resistance and basal versus luminal or mesenchymal versus luminal cell subtype comparisons (Figure 4A). Both MCF10A and MCF7 cell lines were associated with the same phenotype-specific list (*e.g.*, luminal vs. basal and luminal vs. mesenchymal) down and up, respectively. The correlation for MCF10A was with both H3K4 marks while MCF7 correlated predominantly with H3K4ac (Figure 4A). In addition, MCF7 H3K4ac was associated with endocrine response pathways and endocrine therapy resistance which reflects the ESR1+ status of this cell line. Of interest, a higher number of H3K4ac marked gene promoters in the MCF10A cell line were associated with genes down-regulated after knock-down of E-cadherin (CDH1) which plays a role in the dynamic epithelial to mesenchymal transition (EMT) response associated with cancer progression [23].

When comparing MCF10A to MDA-MB-231 (Figure 4B) we identified MSigDB gene lists associated with cellular subtype classification, proliferation, serum response genes regulated during cell cycle, and metastasis. When compared to MCF10A, changes in H3K4me3 and H3K4ac in MDA-MB-231 were associated with basal/luminal vs. mesenchymal down or up, respectively. Notably, the most significant and highest number of gene sets intersected with H3K4ac for MDA-MD-231 when compared to MCF10A and were associated with proliferation, serum response genes regulated during the cell cycle and genes upregulated with advanced grade of invasive breast cancer.

A comparison of the two breast cancer cell lines (MCF7 vs. MDA-MB-231; Figure 4C) identified many of the same MSigDB gene expression sets as were previously identified when comparing each cancer cell line against MCF10A. This suggests that the contrast of H3K4ac enrichment profiles between a normal epithelial cell (MCF10A) and ESR1+/PGR+ luminal cell (MCF7) or triple negative mesenchymal (MDA-MB-231) are dictated by phenotypic traits. Although comparison of H3K4ac in MCF7 to MDA-MB-231 returns many of the same gene sets, we see the addition of H3K4me3 mark associated with the gene sets. Of interest, in the comparison of MCF7 to MDA-MB-231 a new gene set, mammary stem cells, was associated with change in H3K4ac (MCF7) and H3K4me3.

We next wanted to determine if the same trends in H3K4ac and H3K4me3 would persist outside of the queried set of dynamically marked gene promoters by clustering all genes in the MSigDB lists (Figure 4D-4G). Identified highly enriched genes used to detect MSigDB lists were excluded and the remaining genes were clustered by k-means for comparison.

From the GOZGIT_ESR1_TARGETS_DN gene set [24], 113 genes were identified as highly acetylated in MCF7 compared to MCF10A, an additional 357 genes of the 728 total genes in this list exhibit the same trend but at a lower level of differential enrichment (Figure 4D). Within the CHARAFE_BREAST_CANCER_LUMINAL_VS_BASAL_UP gene set [25], MCF7 had higher enrichment than MCF10A for H3K4ac in 3 out of 4 clusters, encompassing 204 of 305 genes (Figure 4E). In contrast, for the CHARAFE_BREAST_CANCER__LUMINAL_VS_BASAL_DN gene set, MCF10A gene promoters showed a less distinct pattern of differential acetylation and methylation. However a substantial portion of genes (119 of 414) in the acetylation list follow the same trend as the query set (Figure 4F). When comparing MCF10A to MDA-MB-231, in the context of gene lists associated with SOTIRIOU_BREAST_CANCER_GRADE_1_VS_3_UP gene set [26] we observed 112 genes of 127 following the same trend as our 19 highly H3K4ac enriched in MDA-MB-231 (Figure 4G). It should be noted that H3K4me3 enrichment is also higher in MDA-MB-231 cell line for a set of genes within this list.

Overall for the lists identified, the trend exhibited by the highly enriched query set extended to a substantial number of additional genes in each list providing support that H3K4ac at promoters is predictive of gene sets that are differentially expressed in defined cancer etiologies. Supplementary Figure S2 contains heat maps for every overrepresented MSigDB gene set. Although we observed correlation with both H3K4me3 and H3K4ac, the presence of H3K4ac is more closely associated with an epigenetic signature of breast cancer than the more widely studied H4K4me3 histone modification.

**Figure 5:**
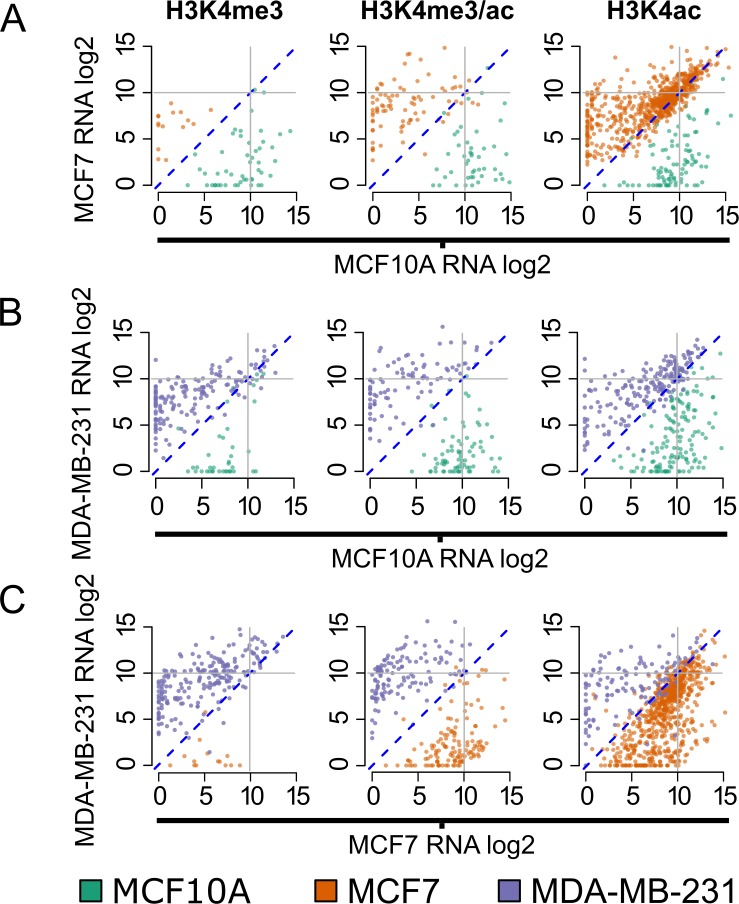
Genes dynamically marked by H3K4me3/ac are differentially expressed Cell line comparisons showing normalized (log2) gene expression for gene promoters differentially marked by H3K4me3, H3k4me3/ac and H3K4ac. Each point represents the expression level and the color denotes the cell line with a ≥ 4-fold change in histone modification enrichment: green (MCF10A), orange (MCF7), and purple (MDA-MB-231). Pairwise comparisons are **A**. MCF7 versus MCF10A, **B**. MDA-MB-231 versus MCF10A and **C**. MDA-MB-231 versus MCF7. Diagonal (dotted) line represents log2 0 (no change) and solid (gray) lines demarcates highly expressed genes (> 10 log2) in each cell line.

We next validated the differentially marked gene sets used to query MSigDB with RNA-seq data generated using the three cell lines (Figure 5A-5C). Gene expression levels for promoters differentially marked by H3K4ac, H3K4me3, or both modifications were compared for each pair of cell lines. When comparing MCF7 to MCF10A (Figure 5A), genes which are associated with changes in H3K4ac (alone or with H3K4me3) have high (> 10 log2) average expression levels. In addition, genes associated with H3K4ac alone show > 6 fold increase in expression levels compared to H3K4me3 alone (median normalized counts: 617.36 versus 100.43). We observed a similar increase in expression levels in MCF10A when comparing it to MDA-MB-231, and again in MCF7 when comparing it to MDA-MB-231. These findings suggest that H3K4ac is not only associated with cancer-related genes (as defined by our query of MSigDB, Figure 4), but strongly correlates with the level of gene expression.

### H3K4ac enrichment correlates with cancer associated pathways

**Figure 6:**
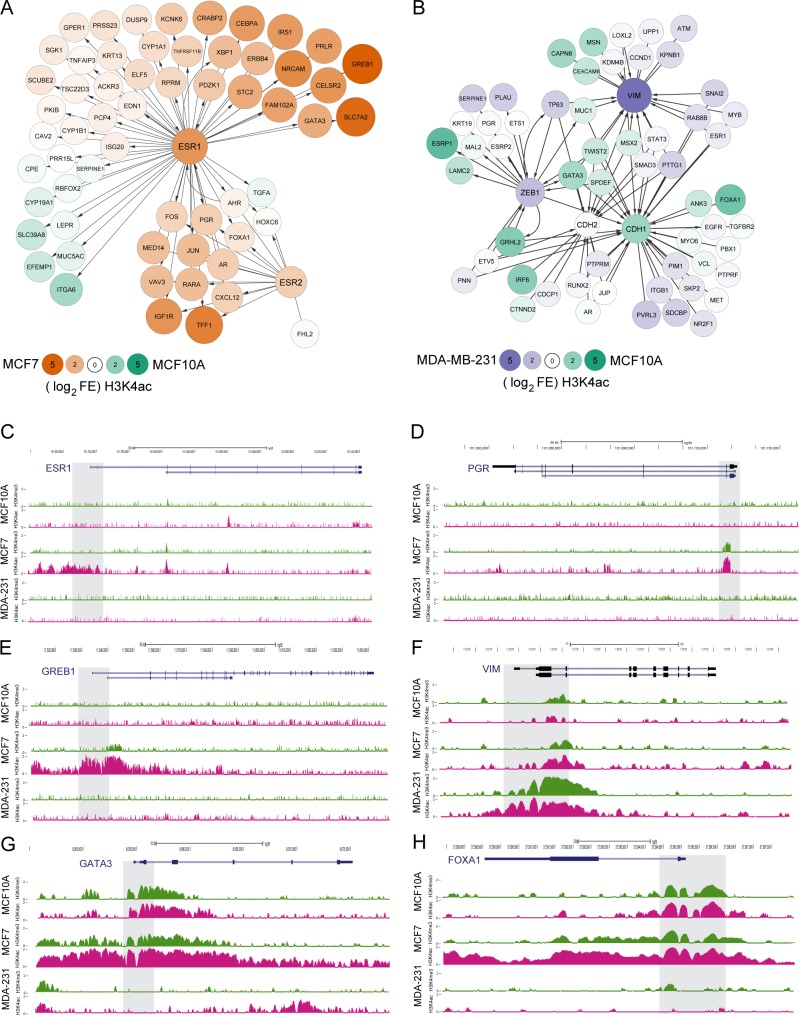
H3K4ac is enriched at promoters of transcribed genes in regulated pathways in cancer Changes in H3K4ac enrichment for genes related to endocrine response and EMT response. **A.** Endocrine response genes were defined by GOZGIT_ESR1 targets up or down lists that also directly interact with ESR1 or ESR2 by IPA and demonstrate relative FC (fold change) of enrichment between cell lines as indicated by color and size of nodes. **B.** EMT response genes were defined by CHARAFE luminal/basal/mesenchymal cell type comparison gene sets that are also directly connected to VIM, ZEB1, CDH1, and CDH2 by IPA and demonstrate relative FC of enrichment between cell lines as indicated by color and size of nodes. **C.**-**H.** Examples of the localization pattern and differential enrichment of H3K4me3 and H3K4ac at individual genes associated with breast cancer. In MCF7 cells, higher levels of H3K4ac enrichment were observed near promoters (grey shading) of genes involved in estrogen response including **C.** ESR1 (estrogen receptor α), **D.** PGR (progesterone receptor), and **E.** GREB1 (growth regulation by estrogen in breast cancer 1) an early estrogen-responsive gene. Genes involved in regulation of critical proteins involved in EMT demonstrated cell specific patterns of H3K4ac enrichment at gene promoters. **F.** H3K4ac enrichment at the VIM (vimentin) promoter was elevated in MDA-MB-231 while G. GATA3 and H) FOXA1 demonstrate a relative loss of H3K4ac enrichment in MDA-MB-231 cells compared to MCF7 and MCF10A cells.

From our analysis of MSigDB gene expression and RNA-seq datasets, we identified two molecular pathways associated with cancer phenotypes. We found the ESR1 pathway, corresponding to endocrine response (GOZGIT) for MCF10A to MCF7 (Figure 6A), and the breast cancer cell line subtype characterization (CHARAFE) for MCF10A to MDA-MB-231, corresponding to EMT related genes [25, 27], were correlated with differential H3K4ac enrichment (Figure 6B).

Using Ingenuity Pathway Analysis (IPA) we observe a large number of genes within the ESR1 and ESR2 pathways that are marked by increased H3K4ac in the MCF7 cell line relative to the normal-like MCF10A cell line. Several of the genes are breast cancer related, including growth regulation by estrogen in breast cancer 1 gene (GREB1) that is reported to be highly expressed in hormone responsive tissues in cancer [28-30], the transcription factor GATA3, known to play a role in luminal breast cell differentiation and is implicated in breast cancer progression [31-33], and SLC7A2 a less well described cationic amino acid transporter known to exhibit moderate to low levels of protein expression in breast cancer with no expression observed in normal breast epithelium [34]. A smaller number of genes are differentially marked by H3K4ac in the MCF10A cell line including Integrin alpha 6 (ITGA6), which is involved in cell adhesion and cell-surface mediated signaling [25] and transforming growth factor alpha (TGFA) a growth factor ligand involved in cell proliferation, differentiation and normal cell development [35].

For the MCF10A to MDA-MB-231 comparison, MSigDB gene expression datasets defining breast cancer cell subtypes were used to select well-established EMT associated genes (VIM, CDH1, ZEB1) as central nodes to define an IPA gene association network (Figure 6B). E-cadherin (CDH1), which is expressed in epithelial tissues, is marked by H3K4ac in the normal-like epithelial derived MCF10A cell line as is TWIST2 and ANK3. VIM, ZEB1, and SMAD3 show enrichment of H3K4ac in MDA-MB-231 and are well-characterized biomarkers in mesenchymal derived cells that have undergone EMT [16]. In addition some genes in the endocrine response pathways are marked by higher H3K4ac enrichment in MCF10A, including GATA3 and its downstream target FOXA1, again suggesting a role of H3K4ac in regulation of endocrine related epigenetic response.

Gene by gene analysis on UCSC genome browser demonstrated that promoters of both estrogen-receptor alpha (ESR1) transcripts had visibly more H3K4 acetylation in MCF7 cells compared to MCF10A and MDA-MB-231 (Figure 6C). In addition, the progesterone receptor (PGR) was marked by H3K4ac in MCF7 and not in the other cell lines (Figure 6D). GREB1 was associated with high amounts of promoter-associated H3K4ac in MCF7 cells and had comparatively low levels of H3K4me3 (Figure 6E), suggesting that H3K4ac may be a primary indicator of transcriptional control of this gene.

Examination of genes associated with EMT demonstrated that, at the individual gene level, increased H3K4ac enrichment could be observed at specific genes in MDA-MB-231, such as vimentin (VIM) (Figure 6F). Greater H3K4ac enrichment could be observed on promoters of GATA3 (Figure 6G) and FOXA1 (Figure 6H) in MCF10A compared to MDA-MB-231. In addition, GATA3 and FOXA1 were associated with the ESR1 pathway (Figure 6A) and have more enrichment of H3K4ac in MCF7 compared to MCF10A.

The clearly defined correlation of H3K4ac enrichment with endocrine responsive gene expression in the MCF7 luminal cell line indicates that H3K4ac should be considered when analyzing epigenetic changes related to hormone response. In addition, the identification by our analysis of several known genes expressed in breast cancer shows that H3K4ac enrichment is more dynamic at cancer-related gene promoters and may be more closely linked to cellular responses such as EMT in breast cancer than the more widely studied H4K4me3 histone modification.

## DISCUSSION

Epigenetic investigations in breast cancer have made it increasingly evident that post-translational histone modifications can provide valuable insight into cancer risk, onset of disease and therapeutic targets. Our studies provide new insight into the characterization of breast cancer phenotypes compared to normal epithelial cells. We focused on histone modifications associated with H3K4, a lysine residue associated with regulation of gene transcription, to evaluate the histone modifications associated with biological responses of epithelial and cancer cells. Several unique features were observed regarding modifications at H3K4, including our observation of the presence of both acetylation and methylation activating modifications in each of the cell lines. One of the hallmarks of cancer progression is the loss of epigenetic regulation leading to aberrant gene expression and ultimately, oncogenic transformation [1, 2]. In our study, we observed a global increase in acetylated H3K4 promoters in both cancer cell lines (MCF7, MDA-MB-231), and a global increase in H3K4 tri- methylated promoters in the MDA-MB-231 metastatic cell line; these findings suggest that oncogenic progression is associated with increases in both of these marks. At the individual chromosome level, changes in H3K4 tri-methylation and acetylation were observed as a function of cancer progression, including on the X-chromosome, which suggests a loss or dysregulation of X-inactivation in both cancer cell lines, consistent with reports of epigenetic instability of the inactive X-chromosome in breast cancer [22].

Methylation and acetylation of H3K4 are both associated with actively transcribed genes; this is in contrast to H3K9 and H3K27, where methylation and acetylation occur at the same amino acid with opposing roles in transcriptional regulation [20, 36]. While there has been extensive analysis of the role of methylation of H3K4, the implications of alternative modifications, such as acetylation, have not been as extensively studied. Work in yeast has shown that H3K4me3 and H3K4ac can be found in the same promoters and that methylation may regulate the abundance and localization of H3K4ac [9]. In addition, studies in drosophila have shown that H3K4ac is selectively enriched on akirin-dependent but not akirin-independent promoters [37]. These authors suggest that replacement of H3K4me3 by H3K4ac is needed for full transcription at a subset of akirin-dependent genes. In addition, a regulator of acetylation, HDAC3, was identified as an important mediator of EMT by recruiting WDR5 to promoters of mesenchymal genes. [10]. The authors observed that H3K4ac at EMT-related genes was removed by HDAC3 during EMT, followed by the addition of H3Kme2/3 to the same amino acid; this result suggests that H3K4me3 and H3K4ac play complementary roles in gene regulation. Together these studies highlight the importance of H3K4 acetylation for support of critical cellular processes.

From our data, a large number of promoters are enriched for H3K4me3 and H3K4ac in the two cancer-related cell lines when compared to a normal breast epithelial cell line. The increase in H3K4ac marks observed in both cancer cell lines compared to the normal-like MCF10A indicates that H3K4ac may represent an early step in cancer progression, while the global increase in H3K4me3 at promoters was primarily observed in the MDA-MB-231 cell line suggesting these changes are more correlative with late stage metastatic cancer. For all the cell lines, gene promoters are differentially marked, with the normal-like MCF10A having fewer marked promoters compared to the cancer cell lines. In addition, we observe an increase in the number of promoters marked by acetylation in the MCF7 cells and an increase in H3K4 tri-methylation in the MDA-MB-231 cells.

Examination of differential enrichment of H3K4ac/me3 at gene promoters was also informative in defining specific biological pathways integrally involved in cancer progression. We identified a role for H3K4ac in the cellular ESR response pathway in the MCF7 cells, while H3K4ac together with H3K4me3 is more reflective of the EMT response pathway in the MDA-MB-231 cell line. These results indicate that the H3K4me3 mark in MDA-MB-231 late stage cells identifies cancer-related genes and pathways; however, we find that inclusion of H3K4ac provides increased confidence in association with pathways that correspond to the metastatic cancer phenotype.

By examining sets of genes associated with cancer, we found that H3K4ac enrichment is more informative than H3K4me3 for identifying poised or active transcription. In addition, we found that in MCF7, genes that were dynamically marked by H3K4ac at their promoters demonstrated a higher level of expression with median normalized count values that were > 6 fold greater than MCF10A. Dynamic changes in H3K4ac enrichment were also observed at the individual gene level, specifically in cancer-related genes. For example, GREB1, an estrogen-responsive gene, displayed a robust acetylation pattern with a high level of expression, and no detectable H3K4me3 near the promoter of this gene in MCF7. Our findings highlight another important regulatory dimension that acetylation may represent a more dynamic and readily reversible mark, modulating transcriptional responses to external stimuli, such as ESR signaling. The inclusion of H3K4ac enrichment as an indicator of epigenetic changes more accurately defines both genes and pathways involved in specific phenotypes of cancer, providing a more comprehensive picture of regulatory events that contribute to the onset and progression of cancer.

Our findings indicate that H3K4ac is a predictor of deregulated cancer related pathways and that it is a strong indicator of progression from initial transformation to aggressive metastatic phenotypes. Our studies establishing the biological importance of H3K4ac provide a compelling rationale for H3K4ac as a therapeutic target for cancer intervention; however to date no enzymes have been identified that specifically acetylate or de-acetylate H3 lysine 4. An important consideration is the dynamics of acetylation and methylation of H3K4 in supporting gene expression in cancer cells which would involve the activity of several different enzyme complexes. However our results indicate that the gain of acetylation is associated with cancer pathways, and suggest that the role of histone acetyltransferases (HATs) should be further explored. HAT inhibitors are currently in various stages of clinical trials and may prevent cancer progression from the earliest stages.

## MATERIALS AND METHODS

### Cell culture and reagents

MCF10A cells were grown in DMEM: F12 (Hyclone: SH30271), 5% (v/v) horse serum (Gibco: 16050) + 10 μg/ml human insulin (Sigma: I-1882) + 20 ng/ml recombinant hEGF (Peprotech: AF-100-15) + 100 ng/ml cholera toxin (Sigma: C-8052) + 0.5 μg/ml hydrocortisone (Sigma: H-0888) 50 IU/ml penicillin/50 μg/ml streptomycin and 2 mM glutamine (Life Technologies: 15140-122 and 25030-081, respectively). MCF7 cells and MDA-MB-231 were grown in DMEM: F12 + 10% (v/v) FBS (Atlanta Biologicals: S11550). Cell lines were validated by short tandem repeat (STR) analysis using the Promega GenePrint® 10 System at the UVM Cancer Center DNA Analysis Facility according to manufacturer's instructions (Promega: B9510). STR profiles were confirmed to be identical to known STR fingerprints in ATCC STR Profile (http://www.atcc.org/STR_Database) and/or Cell Line Integrated Molecular Authentication database (CLIMA) database version 0.1.200808) (http://bioinformatics.istge.it/clima/) [38].

### Chromatin immunoprecipitation

Cells were seeded at 1.5×10^6^ cells per 100 mm dish and grown to 80% confluence before harvesting. For fixation, cells were washed twice with 10 ml of warm PBS and formaldehyde (Sigma: F8775) added to final concentration of 0.8%. Cells were cross-linked for 10 minutes at room temperature and formaldehyde neutralized with excess glycine (0.125M) for 5 minutes. Cells were then washed with ice-cold PBS + Roche-Protease cOmplete Inhibitor Cocktail (Roche: 04693132001), dissociated by scraping, collected by centrifugation, flash frozen and stored at −80°C. Nuclei extraction was performed using a protocol modified from Dignam *et al.* [39] and previously described in detail [40]. Isolated nuclei were sonicated using a Covaris S-220 ultrasonic processor to shear chromatin to an average fragment size of 500 bp. A total of 30 μg of sheared chromatin was used for immunoprecipitation with 10 μg of anti-H3K4me3 (Abcam: ab1012) or anti-H3K4ac (Active Motif: 39381) antibody. Immune complexes were then isolated by addition of 50 μl of pre-washed (0.5% BSA/PBS solution) Protein-G Dynabeads (Life Technologies: 10004D) for 4 hours with constant rotation at 4°C. The immunoprecipitated complexes were then sequentially washed with 3 × 1 ml cold RIPA buffer (150mM NaCl); 2 × 1 ml cold high-salt RIPA buffer (500mM NaCl); and 1 × 1 ml TE + NaCl (10mM Tris, 1mM EDTA, and 50mM NaCl). Protein-associated DNA fragments were recovered by heating overnight at 65°C, treated with 0.2 mg/ml RNase A (Life Technologies: AM2269) for 2 hours at 37°C, followed by treatment with 0.2 mg/ml proteinase K (Life Technologies: AM2548) at 55°C for 2 hours. Samples were then phenol extracted, precipitated with ethanol, with final re-suspension in 10 mM Tris-HCl. DNA was then quantified by Qubit fluorimeter (Life Technologies) prior to proceeding to the library preparation.

### Library and sequencing preparation

Sequencing libraries were prepared using either TruSeq ChIP sample preparation kit (Illumina: IP-202-1024) or TruSeq Stranded Total RNA LT with Ribo-Zero Gold Kit (Illumina: RS-122-2301) following the manufacturer's instructions. RNA was isolated using Qiagen RNeasy Plus kit (Qiagen: 74134) following manufacturer's directions, analyzed for RNA integrity and then, amplified and adaptered. Briefly for ChIP libraries, samples were processed for end-repair, A-tailing and adapter ligation using ChIP-DNA at a concentration of 120-160 pg/μl in a volume of 50 μl in a 96-well microplate. DNA was recovered between each enzymatic treatment step using AMPure XP beads (Beckman Coulter: A63881) at a 1:1 (DNA: bead) ratio. After adapter ligation, excess adapters and adapter dimers were removed using DNA:AMPure XP beads at a 1:1 ratio. Adaptered DNA was then recovered by elution in 30 μl volume of 10 mM Tris-HCl, pH 8.5. Libraries were then amplified by PCR (1 × 98°C - 30 seconds; 14-16 × 98°C - 10 seconds, 60°C - 30 seconds, 72°C - 30 seconds; 1 × 72°C - 5 minutes). ChIP-Seq libraries were size selected by resolving 300-400 bp products on a 2% agarose gel, gel purified on MinElute gel extraction columns (Qiagen: 28604). Final concentrations for all libraries were quantified using the Qubit fluorimeter (Life Technologies), Agilent 2100 Bioanalyzer, and by KAPA library quantification kit (Kapa Biosystems: KK4408) prior to sequencing.

### Bioinformatics analysis

Sequencing base calls were generated on the HiSeq 1500 instrument in the Advanced Genome Technologies Core Massively Parallel Sequencing Facility. For the ChIP-Seq analysis Fastq conversion and demultiplexing were done by bcl2fastq (Ilumina, v1.8.4), evaluated (Fastqc) and processed to remove low quality reads (FastX toolkit). Reads were mapped to the human genome (hg38) using STAR aligner (version 2.4) with splicing disabled (—alignIntronMax 1) [41]. Wig tracks and enriched regions (peak calls) for each replicate were generated by MACS2 [42] and replicates were then evaluated by wigCorrelate [43] and IDR [44]. Biological replicates were then combined and wig tracks regenerated for the combined signal. Values for read alignments and replicate analysis are provided in Supplementary Table S1. All raw data were deposited at the NCBI Gene Expression Omnibus (GSE69377). For RNA-Seq analysis, raw sequences were filtered using cutadapt/fastq quality trimmer [45, 46], aligned to hg38 Tophat (version 2.0.9) [47] with GENCODE v21 supplied as a reference annotation [48]. Reads were quantified using HTSeq-count (version 0.6) [49] and genes with very low expression (< 3 counts) were removed from the analysis. Differential expression was calculated using DESeq2 version 1.4.5 package in R 3.1.0 using the mean value of gene-wise dispersion estimates [50]. RNAseq values for averages across the 3 replicates are provided in Supplementary Table S2. All raw data were deposited at the NCBI Gene Expression Omnibus (reference series - GSE75169).

For analysis of gene promoter regions, annotations were generated by defining the TSS for each protein coding ENSMBL gene (GENCODE release 21) and extending by +/− 1Kb. Enrichment profiles at gene promoters for each cell line was calculated by RPKM^treatment^/RPKM^input^ where input was minimally limited to the 5^th^ percentile of all calculated RPKM (RPKM^total^) using HTseq [49]. Fold enrichment (FE) of chromosomes was determined by calculating RPKM based on total aligned reads and chromosome length, and calculating fold increase over input. Relative enrichment of histone-associated DNA at gene promoters between cell lines was defined by calculating FE (log_2_ B - log_2_ A). Values for all detected gene promoters are provided in Supplementary Table S2. To ensure that signal was quantifiably different between two cell lines a FE value of > 4 was used as a minimum cutoff. For aggregate plots and heatmaps, NGSplot (version 2.47) [51] was used to generate FE profiles from alignment files. In contrast to the +/− 1Kb promoter definition used to calculate values, plot profiles were adjusted to +/− 2Kb from each TSS to better capture the pattern of histone marks at each promoter.

The resultant lists of differentially enriched gene promotors was then used to query 4914 MSigDB gene sets [52] in order to define over-represented gene sets using a binomial test (probability of success = MSigDB list size / 21439 total unique genes in all MSigDB lists; number of trials = size of dynamically marked genes list; number of success = size of intersection of dynamically marked genes list and MSigDB list). Significance of over-representation was determined with a *p*-value threshold of 4.9×10^−9^ after Bonferroni correction.

Heatmaps of each MSigDB list were generated with a *k*-means method (R v 3.2.1) initialized with 4 random centers and allowed to run for 10 iterations or until convergence. Cluster centers were then individually sorted in decreasing order of total FE. Genes used to define MSigDB lists were excluded from clustering and plotted in a separate group presented at the top of each heatmap.

To evaluate molecular pathways associated with differential enrichment of histone marks between cell lines, Ingenuity® Pathway Analysis (IPA) (Qiagen, www.qiagen.com/ingenuity) was used to query interactivity networks. Seed genes (ESR1, ESR2, VIM, ZEB1, CDH1, and CDH2) were used to establish central nodes and related genes (directly interacting molecules from the IPA database) were added and limited to genes also associated with individual, discovered MSigDB lists. The resulting interacting network was then overlaid with FE data (mapped to node size and color) in Cytoscape [53].

## SUPPLEMENTARY MATERIAL FIGURES AND TABLES










