# Supplementary material for: Histone H3 lysine 4 acetylation and methylation dynamics define breast cancer subtypes
Source: Oncotarget. 2016 Jan 15;7(5):5094–109. doi: 10.18632/oncotarget.6922 (PMC4868673; doi:10.18632/oncotarget.6922)
Supplement: Supplementary file 1 [file oncotarget-07-5094-s001.pdf]

Histone H3 lysine 4 acetylation and methylation dynamics define breast cancer subtypes

Supplementary Material

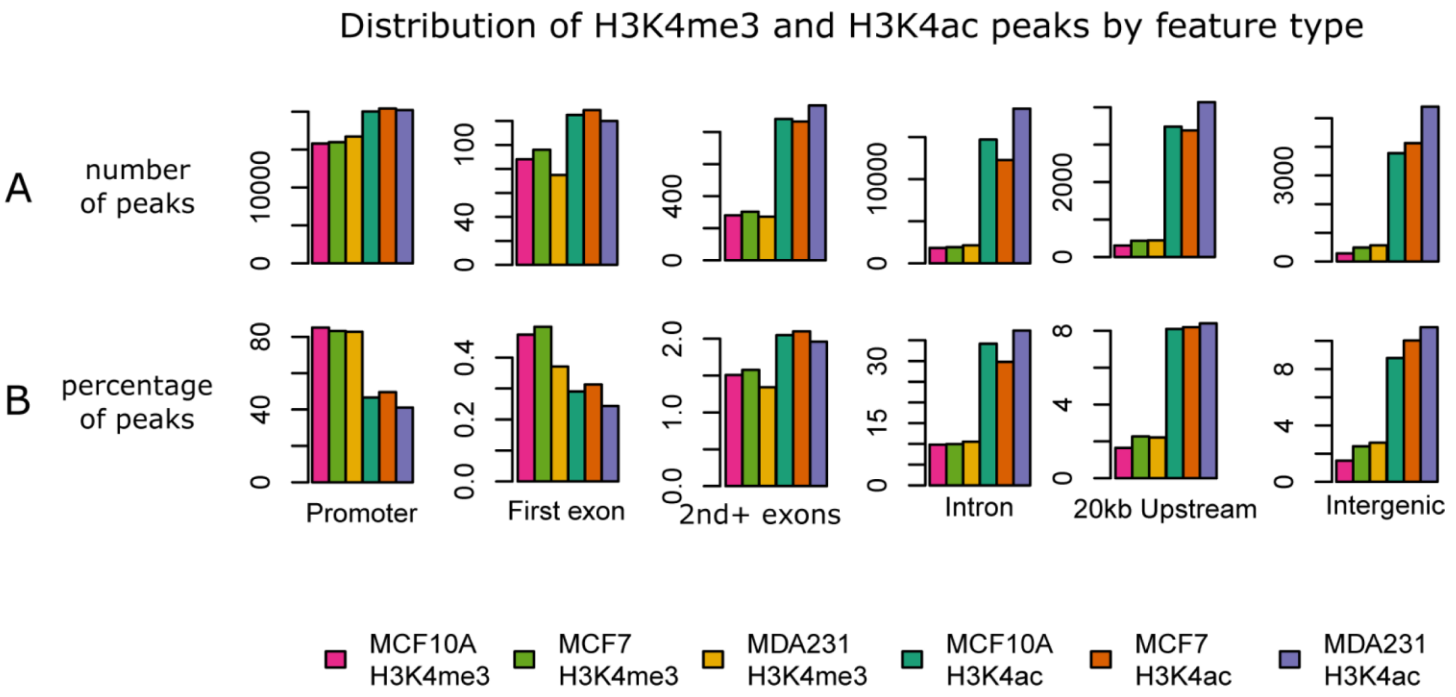

Supplemental Figure 1: Distribution of H3K4me3 and H3K4ac peaks. A. The number of peaks called by MACS2 for the H3K4me3 (first 3 bars) and H3K4ac (second 3 bars) marks in each of the three cell lines: MCF10A, MCF7, and MDA-MB-231. There is one bar plot for each category of genomic location; from left-to-right: promoters (+/- 2kb from TSS), the first exon of each gene, any other exon, introns, within 20kb upstream from TSS, and intergenic (none of the above). **B.** The distribution of peaks as a percentage of total number of peaks called per cell line histone and mark. Approximately 90% of H3K4me3 and 70% of H3K4ac peaks are gene associated (promoter, exon or intron). Given that gene associated groups are 2% of the genome, this suggests that both of these signals very specific to gene regions.
