# Supplementary material for: Histone H3 lysine 4 acetylation and methylation dynamics define breast cancer subtypes
Source: Oncotarget. 2016 Jan 15;7(5):5094–109. doi: 10.18632/oncotarget.6922 (PMC4868673; doi:10.18632/oncotarget.6922)
Supplement: Supplementary file 2 [file oncotarget-07-5094-s002.pdf]

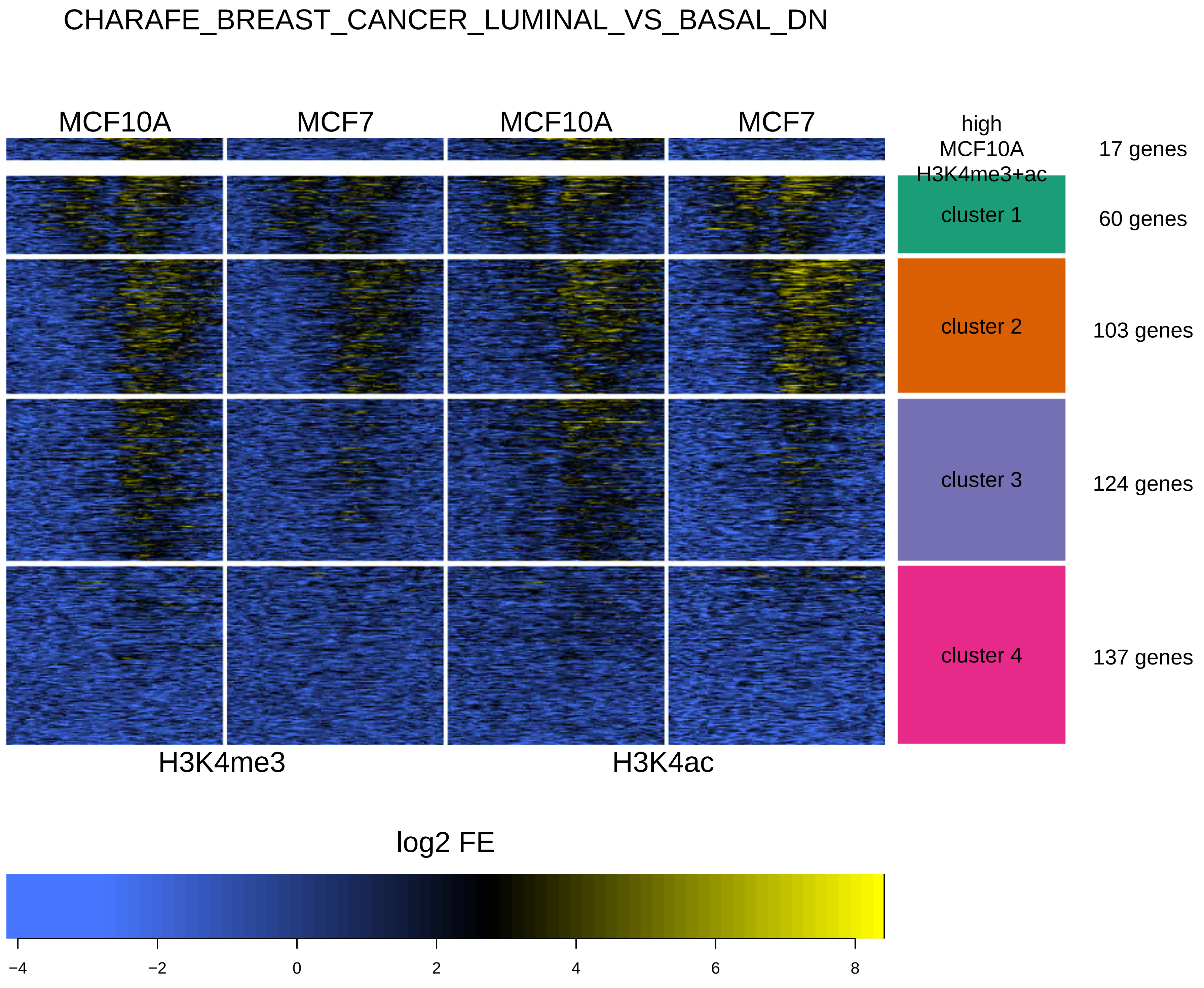

CHARAFE_BREAST_CANCER_LUMINAL_VS_BASAL_DN
MCF10A
MCF7
MCF10A
MCF7
high MCF10A H3K4me3+ac
17 genes
cluster 1
60 genes
cluster 2
103 genes
cluster 3
124 genes
cluster 4
137 genes
H3K4me3
H3K4ac
log2 FE
−4
−2
0
2
4
6
8

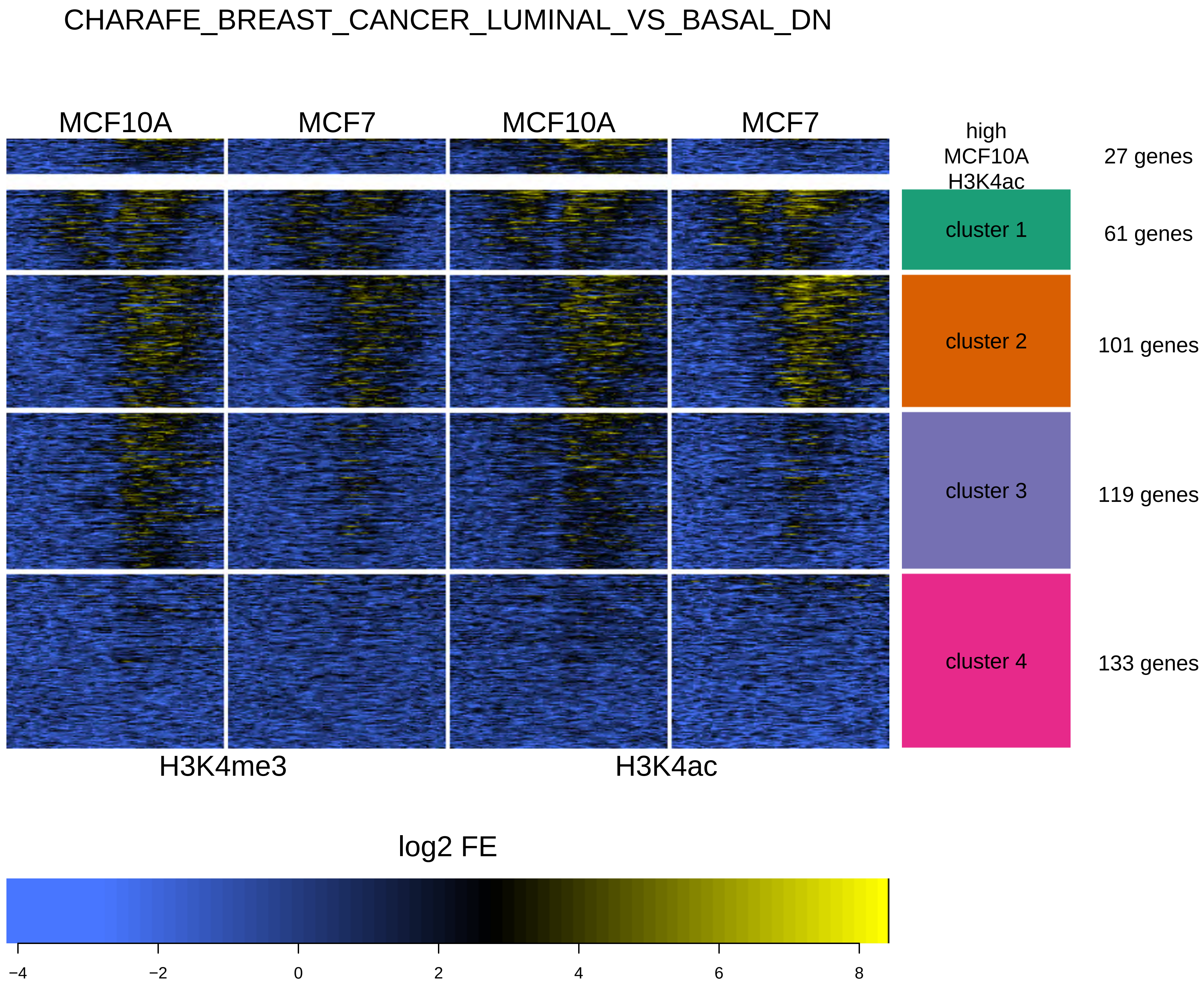
CHARAFE_BREAST_CANCER_LUMINAL_VS_BASAL_DN
MCF10A
MCF7
MCF10A
MCF7
high
MCF10A
H3K4ac
27 genes
cluster 1
61 genes
cluster 2
101 genes
cluster 3
119 genes
cluster 4
133 genes
H3K4me3
H3K4ac
log2 FE
−4
−2
0
2
4
6
8

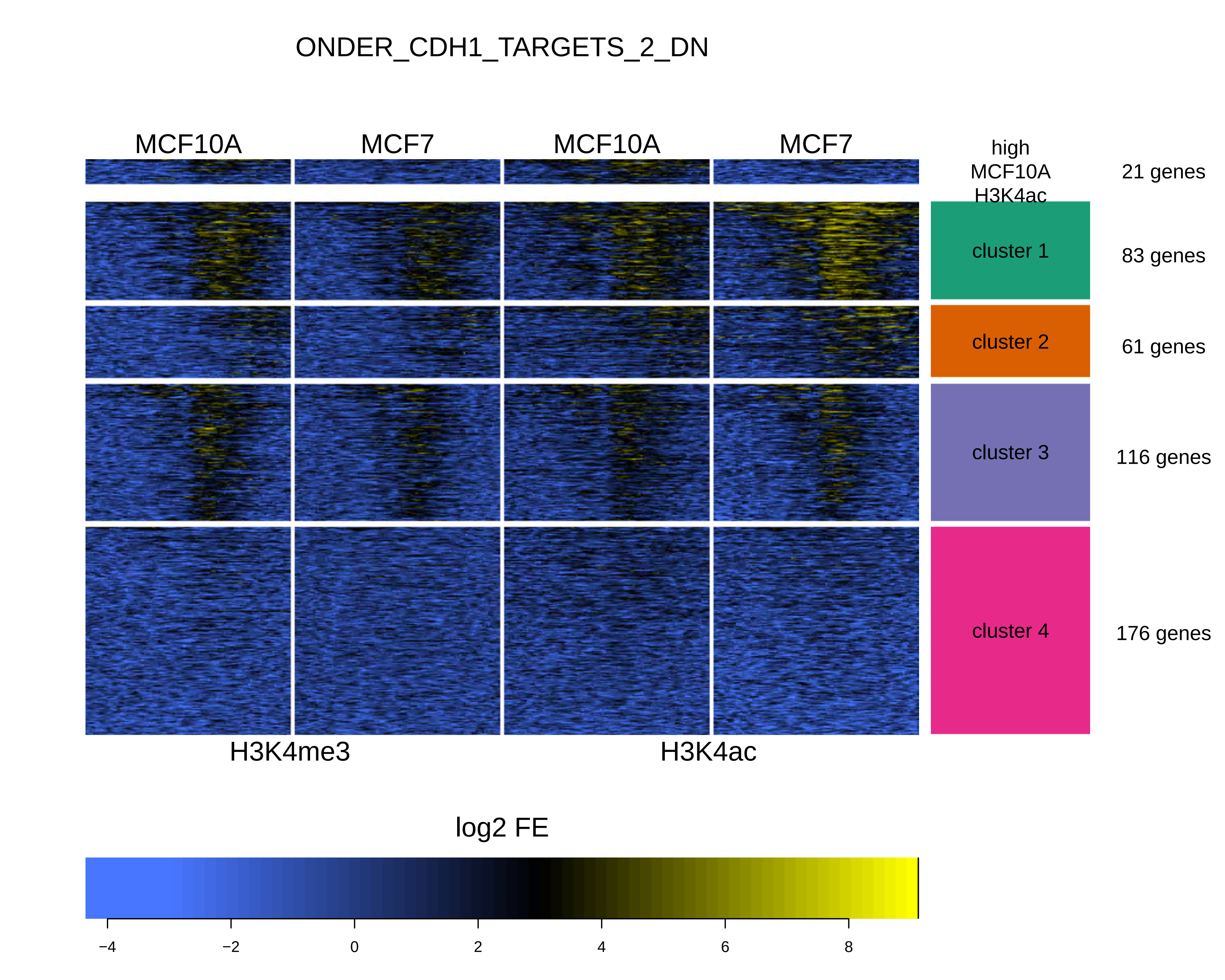
ONDER_CDH1_TARGETS_2_DN
MCF10A
MCF7
MCF10A
MCF7
high
MCF10A
H3K4ac
21 genes
cluster 1
83 genes
cluster 2
61 genes
cluster 3
116 genes
cluster 4
176 genes
H3K4me3
H3K4ac
log2 FE
−4
−2
0
2
4
6
8

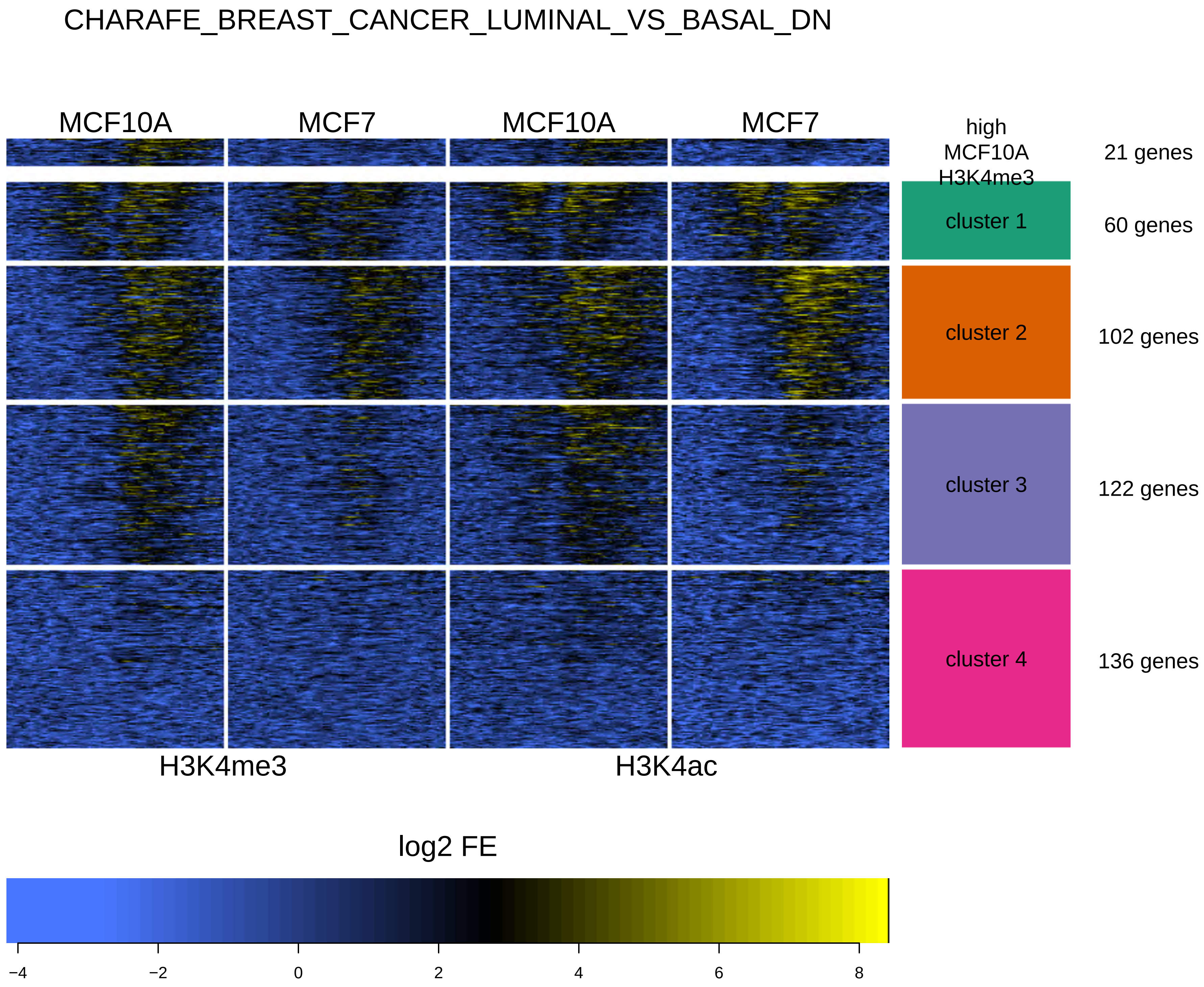
CHARAFE_BREAST_CANCER_LUMINAL_VS_BASAL_DN
MCF10A
MCF7
MCF10A
MCF7
high
MCF10A
H3K4me3
21 genes
cluster 1
60 genes
cluster 2
102 genes
cluster 3
122 genes
cluster 4
136 genes
H3K4me3
H3K4ac
log2 FE
−4
−2
0
2
4
6
8

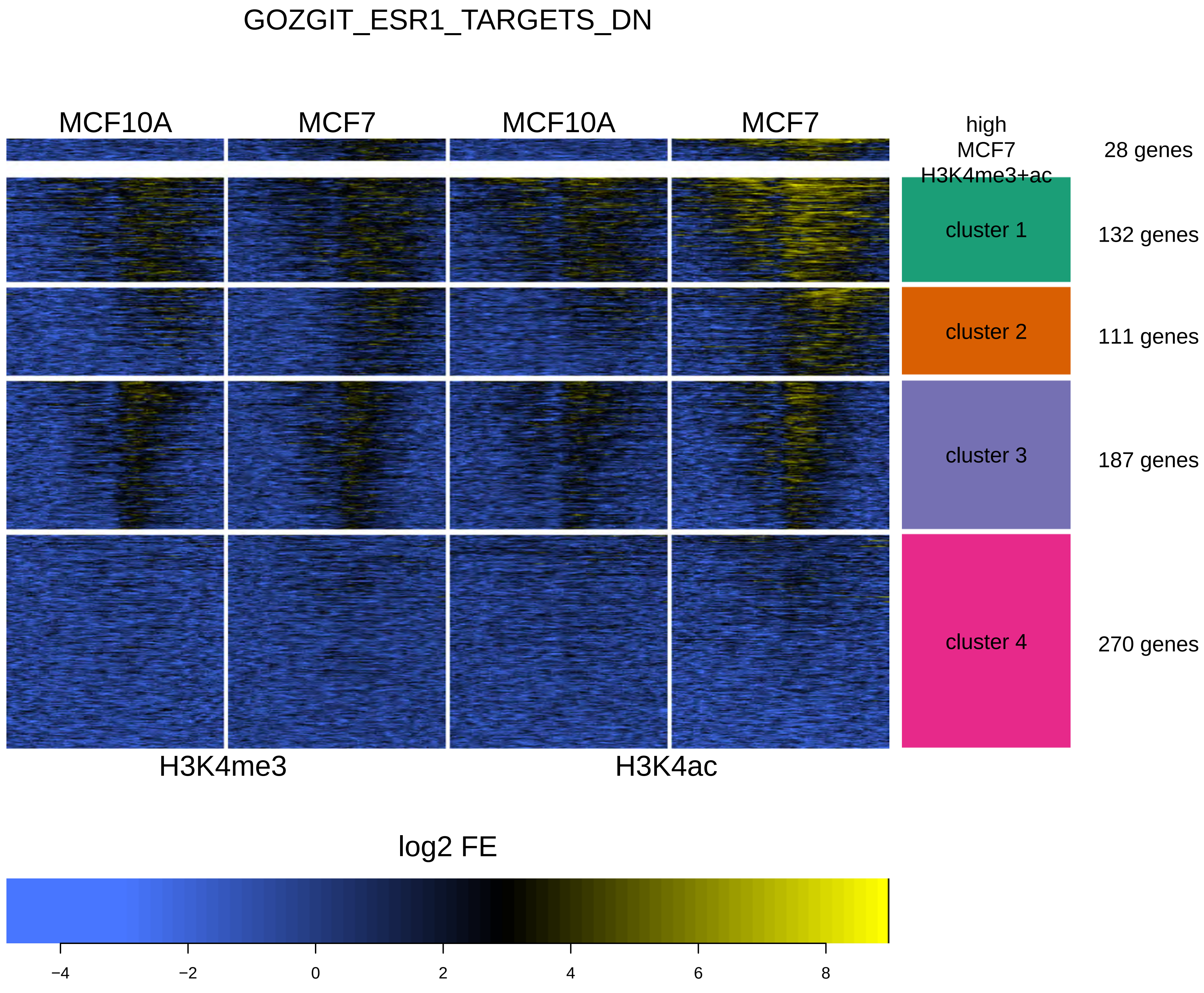
GOZGIT_ESR1_TARGETS_DN
MCF10A
MCF7
MCF10A
MCF7
high MCF7 H3K4me3+ac
28 genes
cluster 1
132 genes
cluster 2
111 genes
cluster 3
187 genes
cluster 4
270 genes
H3K4me3
H3K4ac
log2 FE
−4
−2
0
2
4
6
8

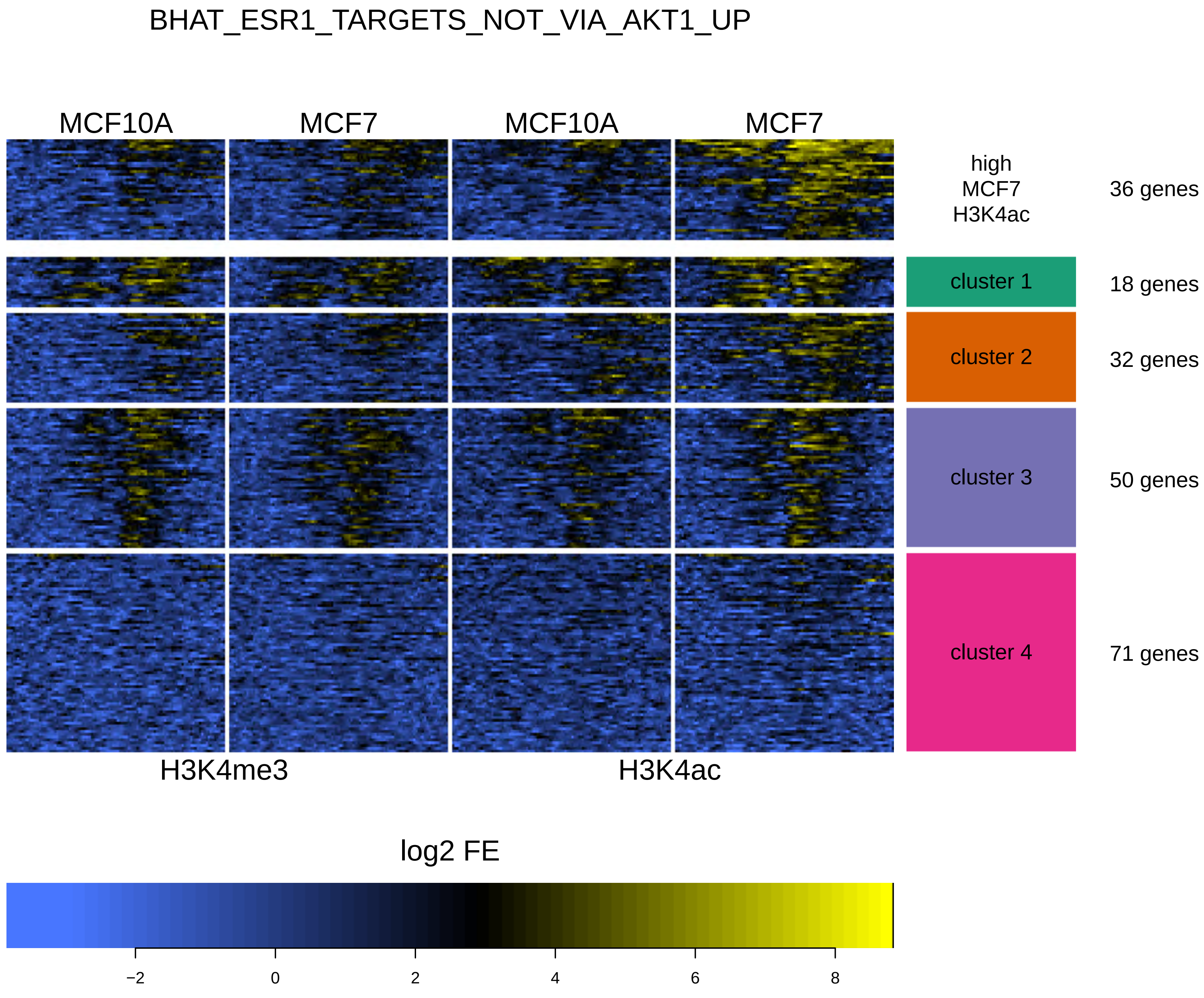
BHAT_ESR1_TARGETS_NOT_VIA_AKT1_UP
MCF10A
MCF7
MCF10A
MCF7
high
MCF7
H3K4ac
36 genes
cluster 1
18 genes
cluster 2
32 genes
cluster 3
50 genes
cluster 4
71 genes
H3K4me3
H3K4ac
log2 FE
−2
0
2
4
6
8

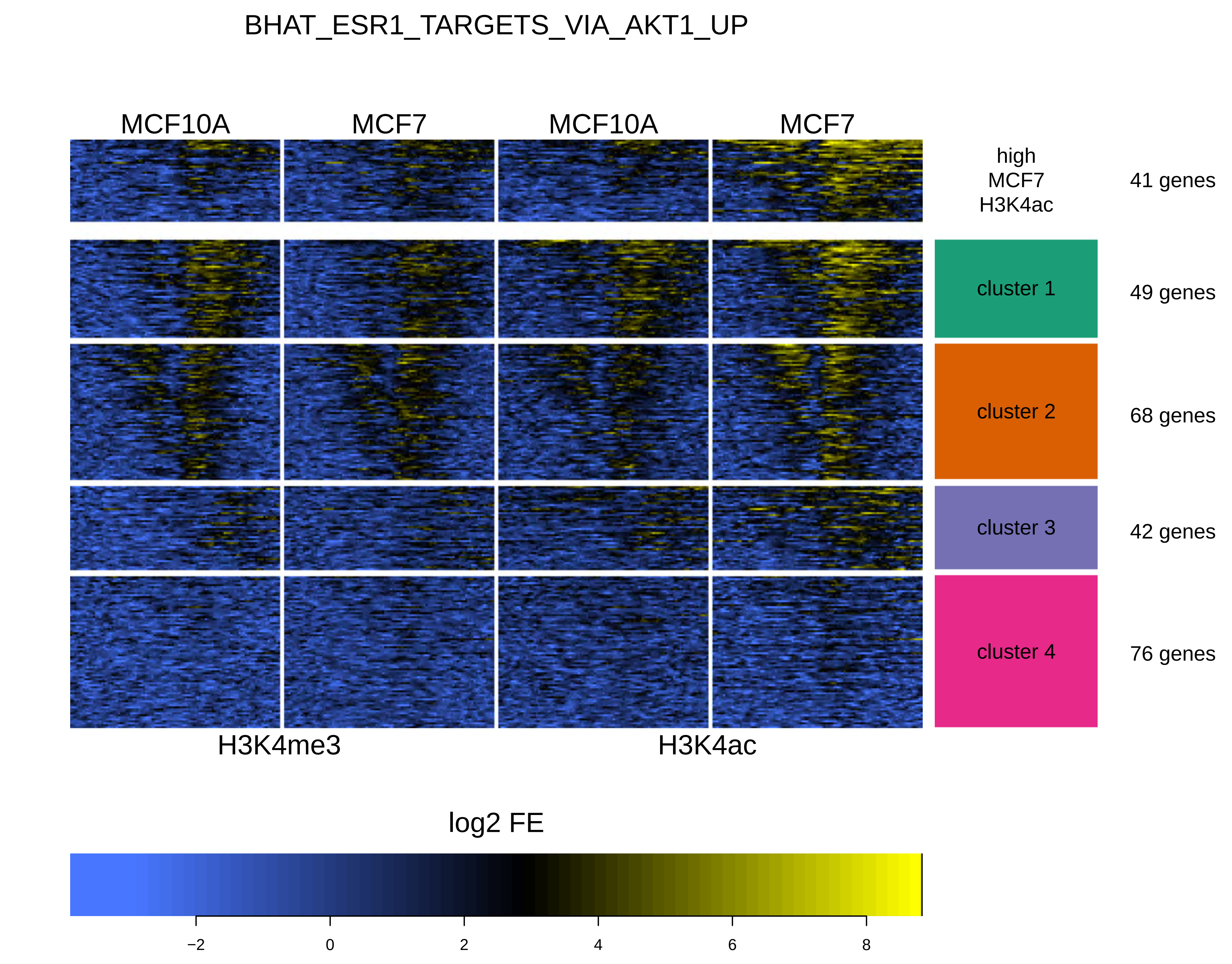
BHAT_ESR1_TARGETS_VIA_AKT1_UP
MCF10A
MCF7
MCF10A
MCF7
high MCF7 H3K4ac
41 genes
cluster 1
49 genes
cluster 2
68 genes
cluster 3
42 genes
cluster 4
76 genes
H3K4me3
H3K4ac
log2 FE
−2
0
2
4
6
8

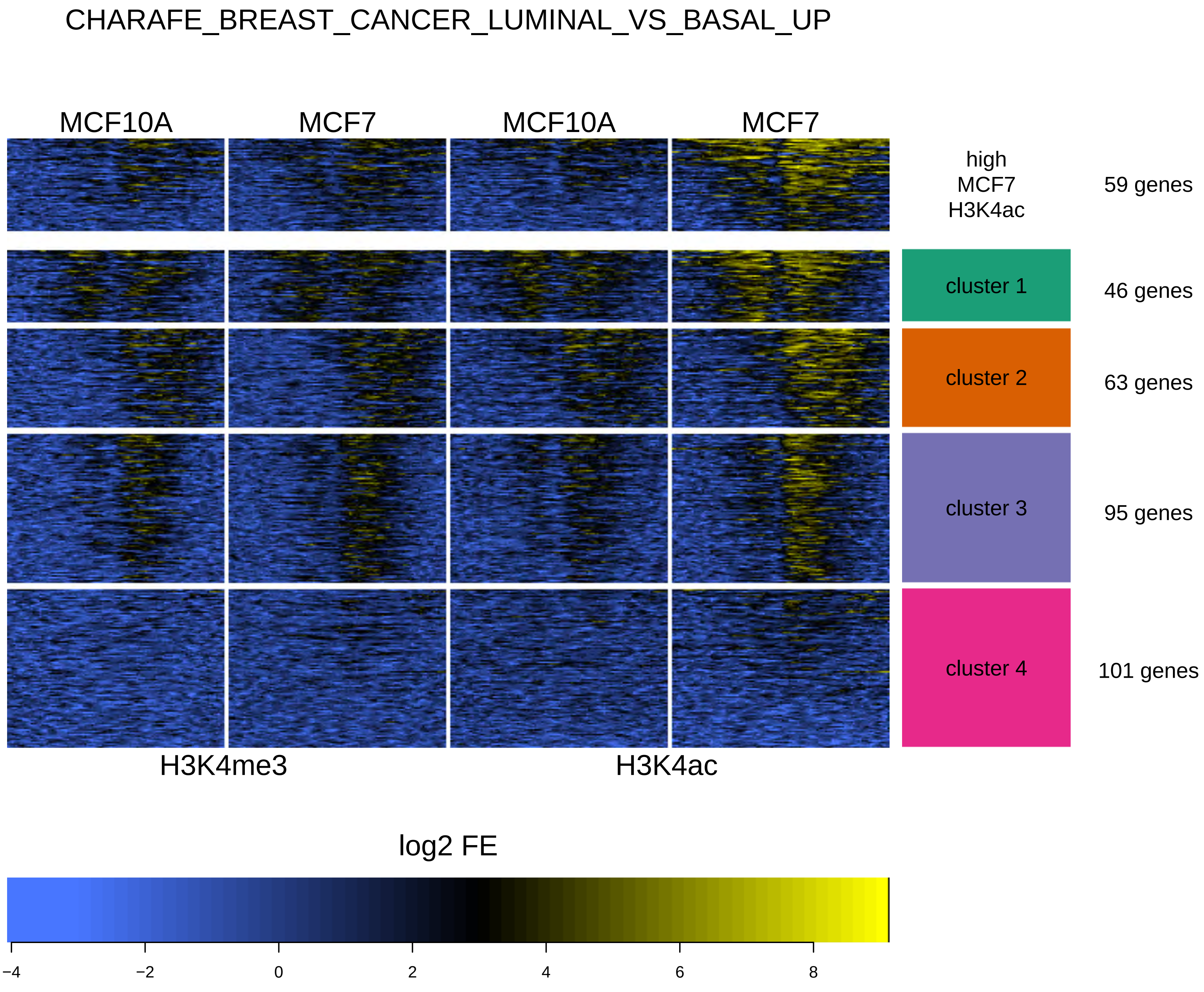
CHARAFE_BREAST_CANCER_LUMINAL_VS_BASAL_UP
MCF10A
MCF7
MCF10A
MCF7
high MCF7 H3K4ac
59 genes
cluster 1
46 genes
cluster 2
63 genes
cluster 3
95 genes
cluster 4
101 genes
H3K4me3
H3K4ac
log2 FE
−4
−2
0
2
4
6
8

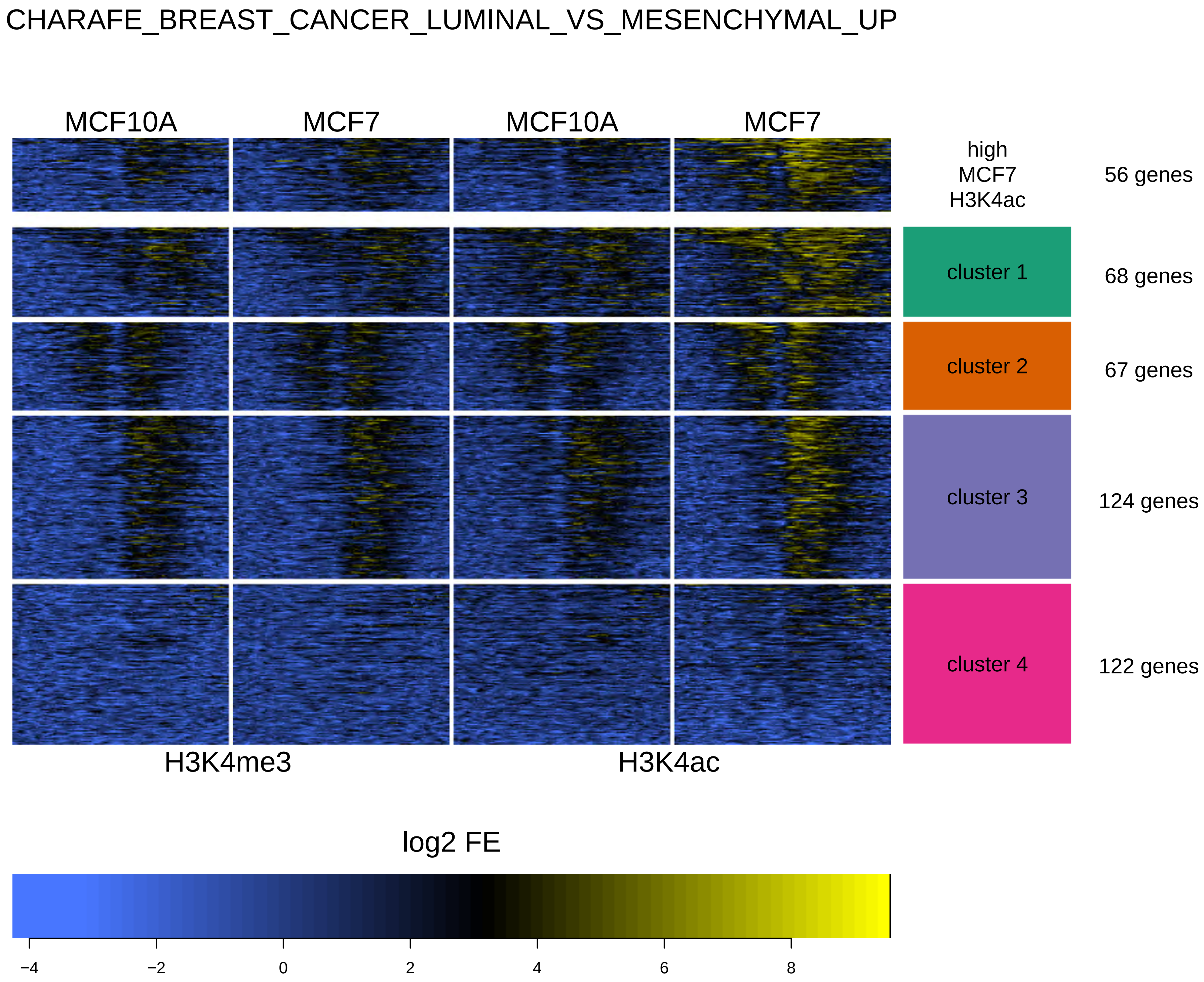

CHARAFE_BREAST_CANCER_LUMINAL_VS_MESENCHYMAL_UP
MCF10A
MCF7
MCF10A
MCF7
high
MCF7
H3K4ac
56 genes
cluster 1
68 genes
cluster 2
67 genes
cluster 3
124 genes
cluster 4
122 genes
H3K4me3
H3K4ac
log2 FE
−4
−2
0
2
4
6
8

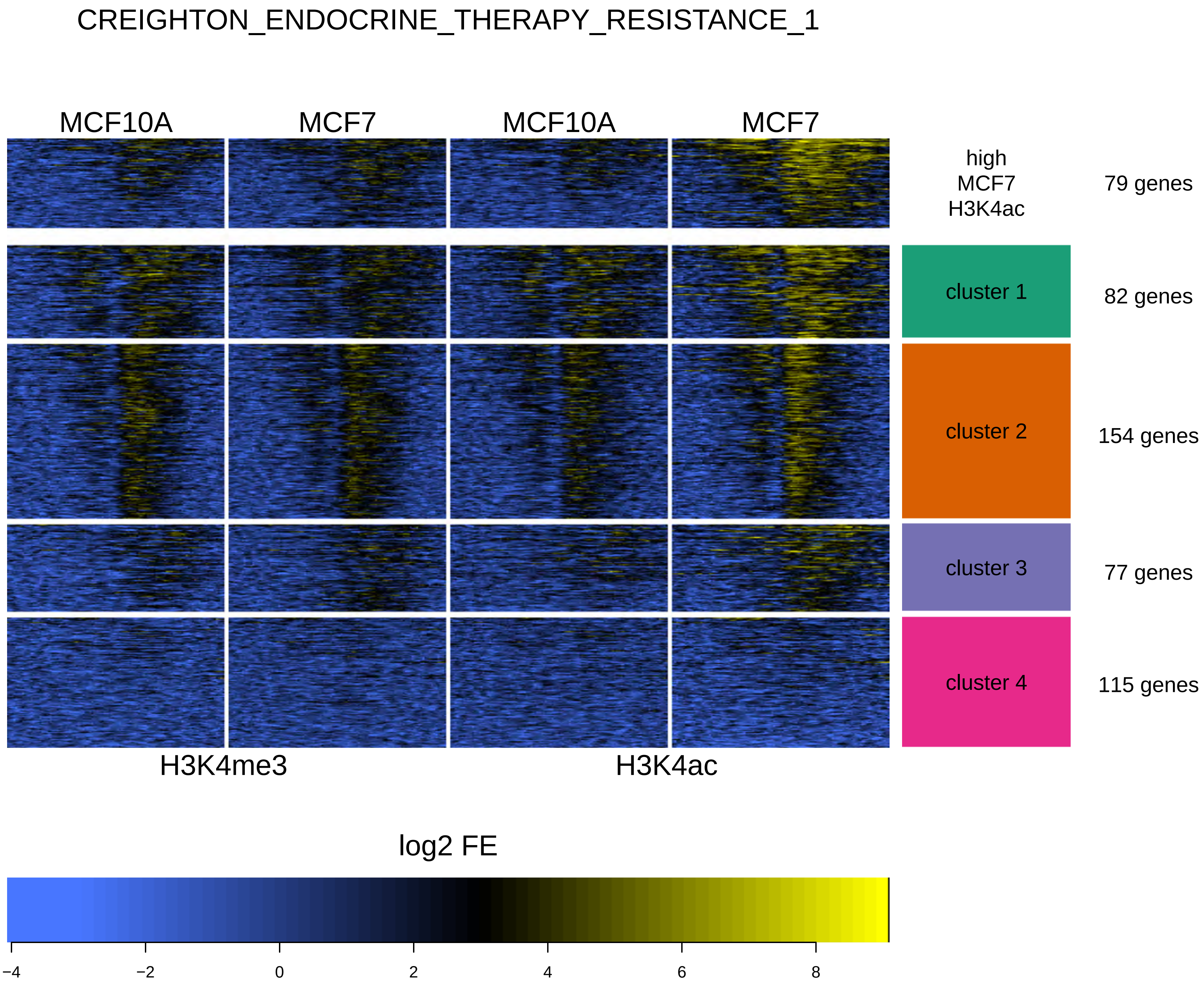
CREIGHTON_ENDOCRINE_THERAPY_RESISTANCE_1
MCF10A
MCF7
MCF10A
MCF7
high MCF7 H3K4ac
79 genes
cluster 1
82 genes
cluster 2
154 genes
cluster 3
77 genes
cluster 4
115 genes
H3K4me3
H3K4ac
log2 FE
−4
−2
0
2
4
6
8

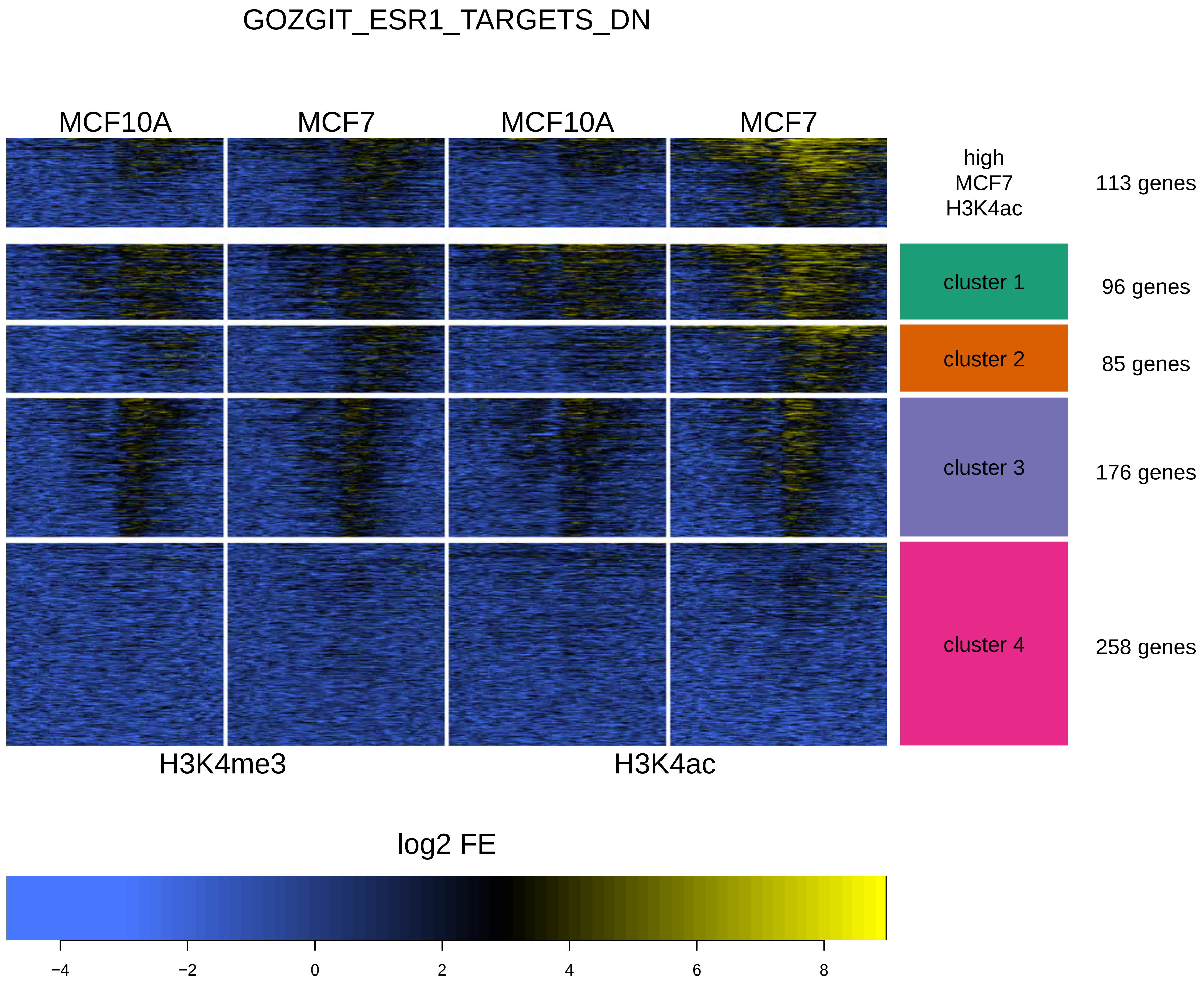
GOZGIT_ESR1_TARGETS_DN
MCF10A
MCF7
MCF10A
MCF7
high
MCF7
H3K4ac
113 genes
cluster 1
96 genes
cluster 2
85 genes
cluster 3
176 genes
cluster 4
258 genes
H3K4me3
H3K4ac
log2 FE
−4
−2
0
2
4
6
8

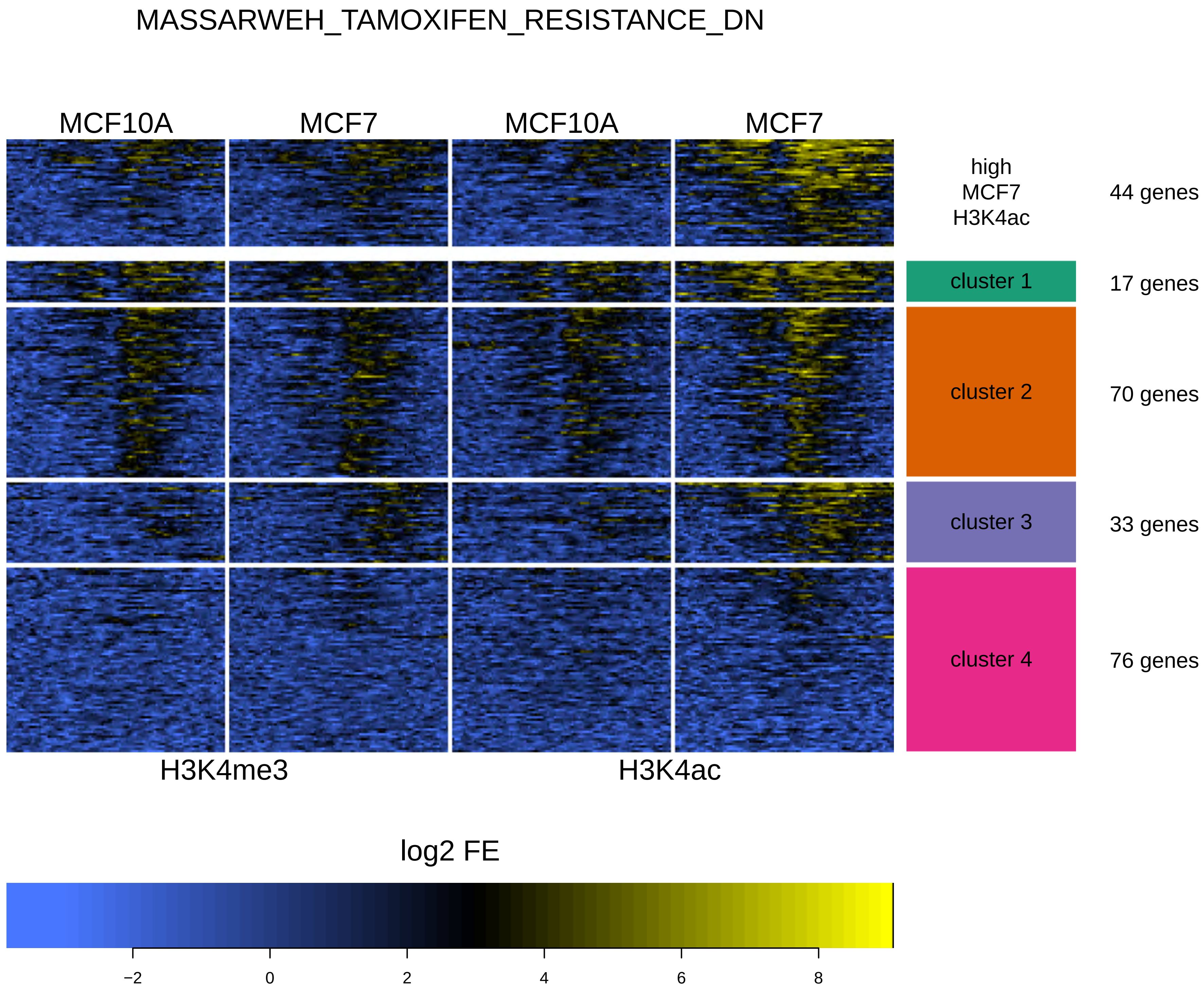
MASSARWEH_TAMOXIFEN_RESISTANCE_DN
MCF10A
MCF7
MCF10A
MCF7
high
MCF7
H3K4ac
44 genes
cluster 1
17 genes
cluster 2
70 genes
cluster 3
33 genes
cluster 4
76 genes
H3K4me3
H3K4ac
log2 FE
−2
0
2
4
6
8

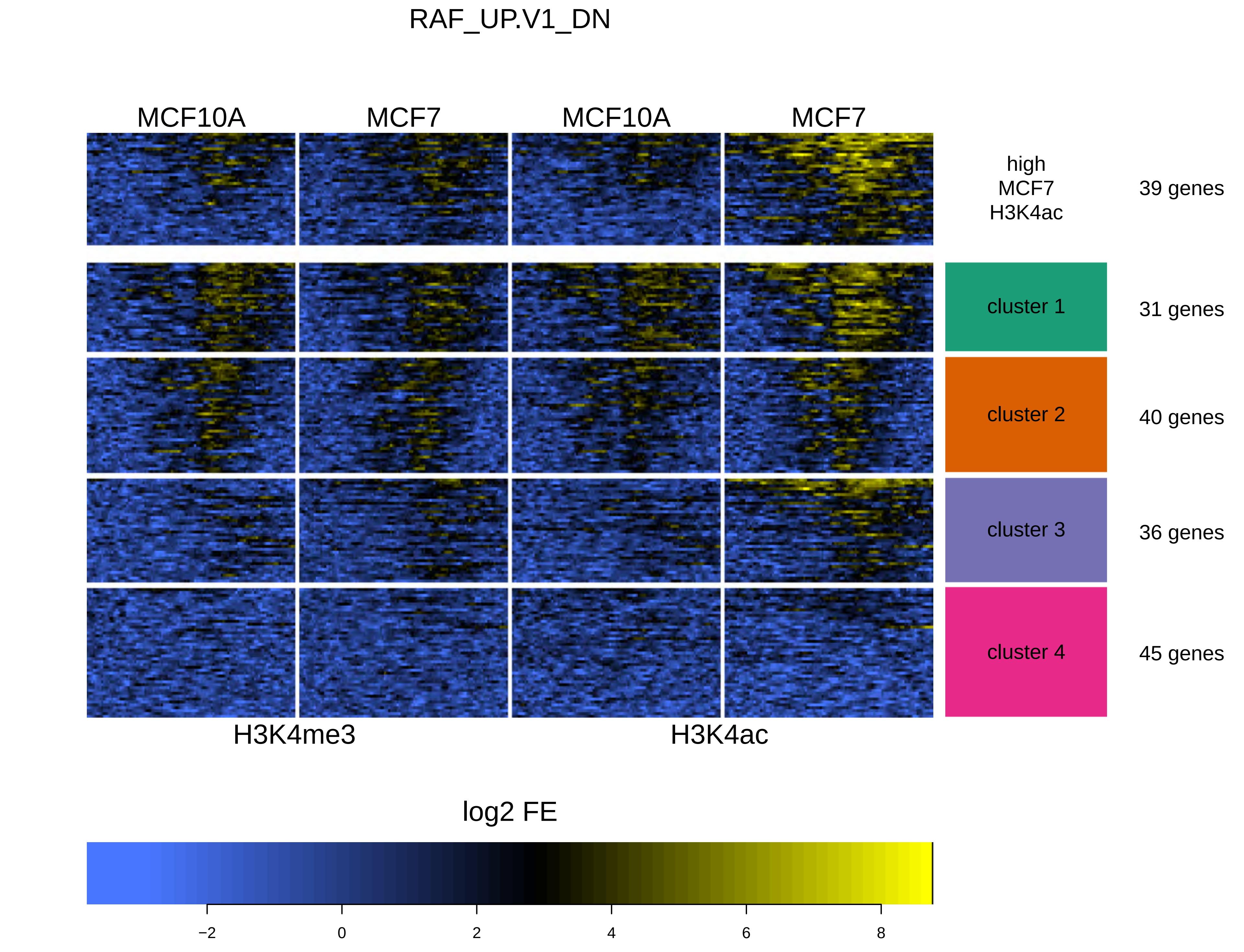
RAF_UP.V1_DN
MCF10A
MCF7
MCF10A
MCF7
high
MCF7
H3K4ac
39 genes
cluster 1
31 genes
cluster 2
40 genes
cluster 3
36 genes
cluster 4
45 genes
H3K4me3
H3K4ac
log2 FE
−2
0
2
4
6
8

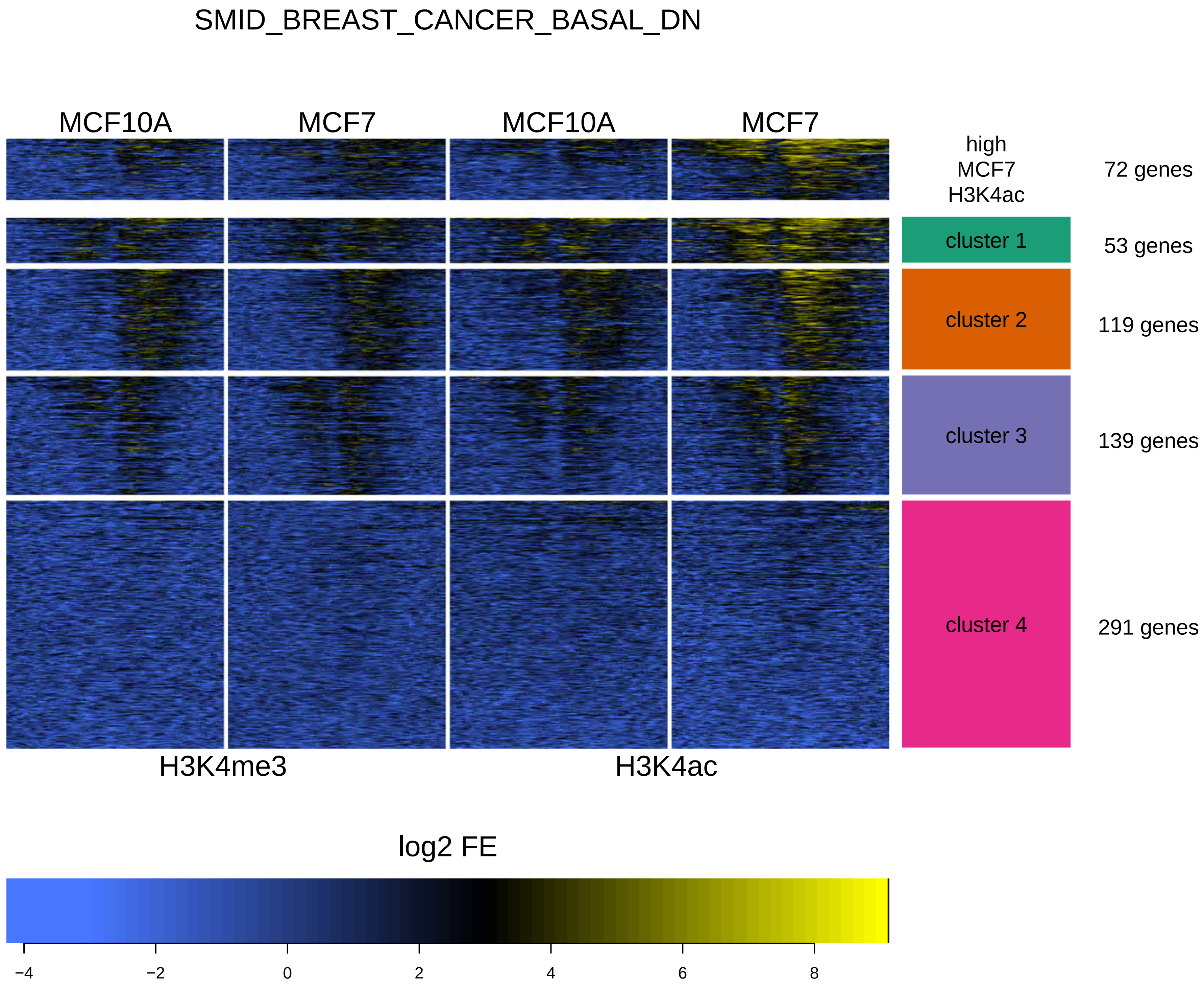
SMID_BREAST_CANCER_BASAL_DN
MCF10A
MCF7
MCF10A
MCF7
high
MCF7
H3K4ac
72 genes
cluster 1
53 genes
cluster 2
119 genes
cluster 3
139 genes
cluster 4
291 genes
H3K4me3
H3K4ac
log2 FE
−4
−2
0
2
4
6
8

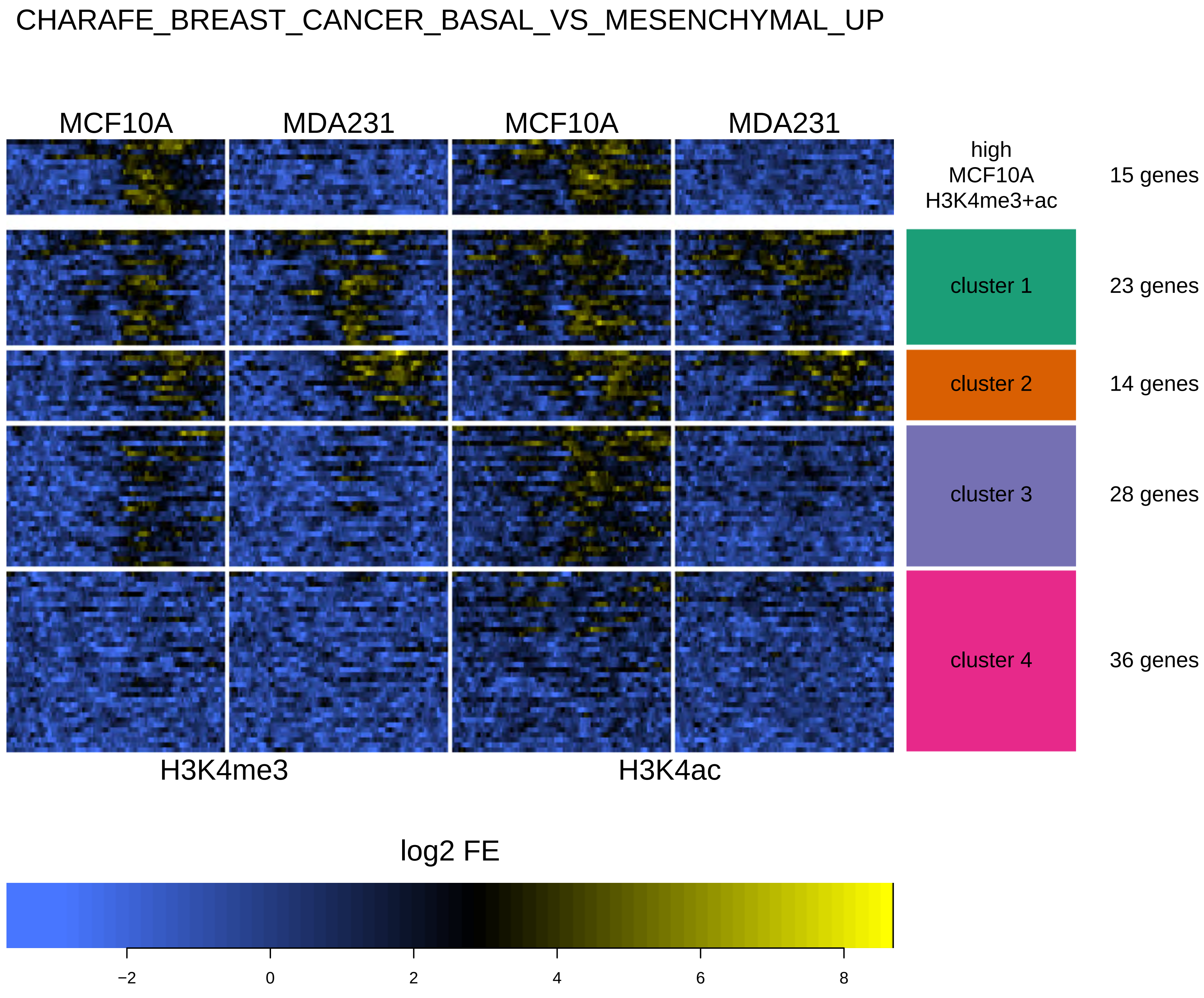
CHARAFE_BREAST_CANCER_BASAL_VS_MESENCHYMAL_UP
MCF10A
MDA231
MCF10A
MDA231
high MCF10A H3K4me3+ac
15 genes
cluster 1
23 genes
cluster 2
14 genes
cluster 3
28 genes
cluster 4
36 genes
H3K4me3
H3K4ac
log2 FE
−2
0
2
4
6
8

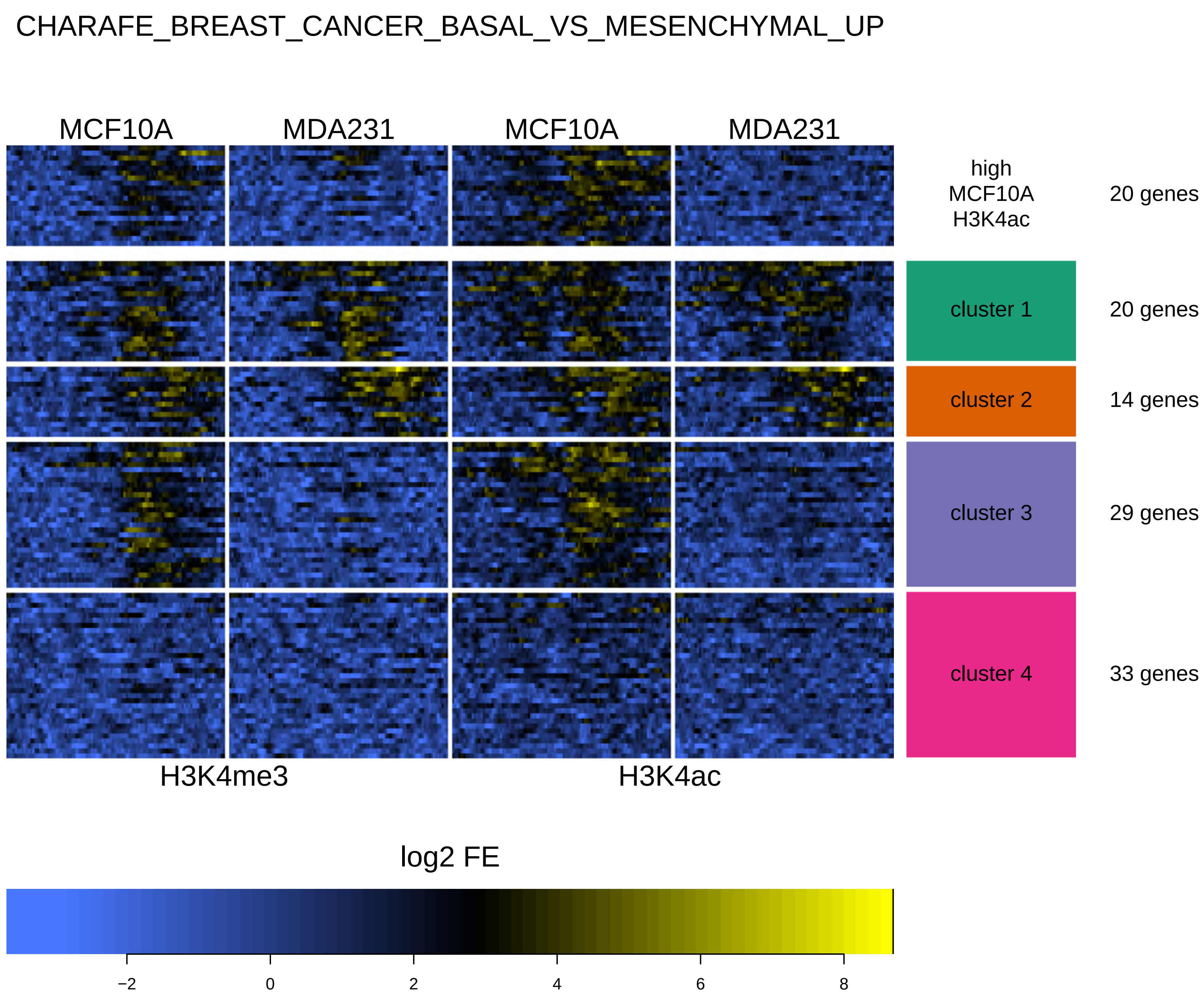
CHARAFE_BREAST_CANCER_BASAL_VS_MESENCHYMAL_UP
MCF10A
MDA231
MCF10A
MDA231
high MCF10A H3K4ac
20 genes
cluster 1
20 genes
cluster 2
14 genes
cluster 3
29 genes
cluster 4
33 genes
H3K4me3
H3K4ac
log2 FE
−2
0
2
4
6
8

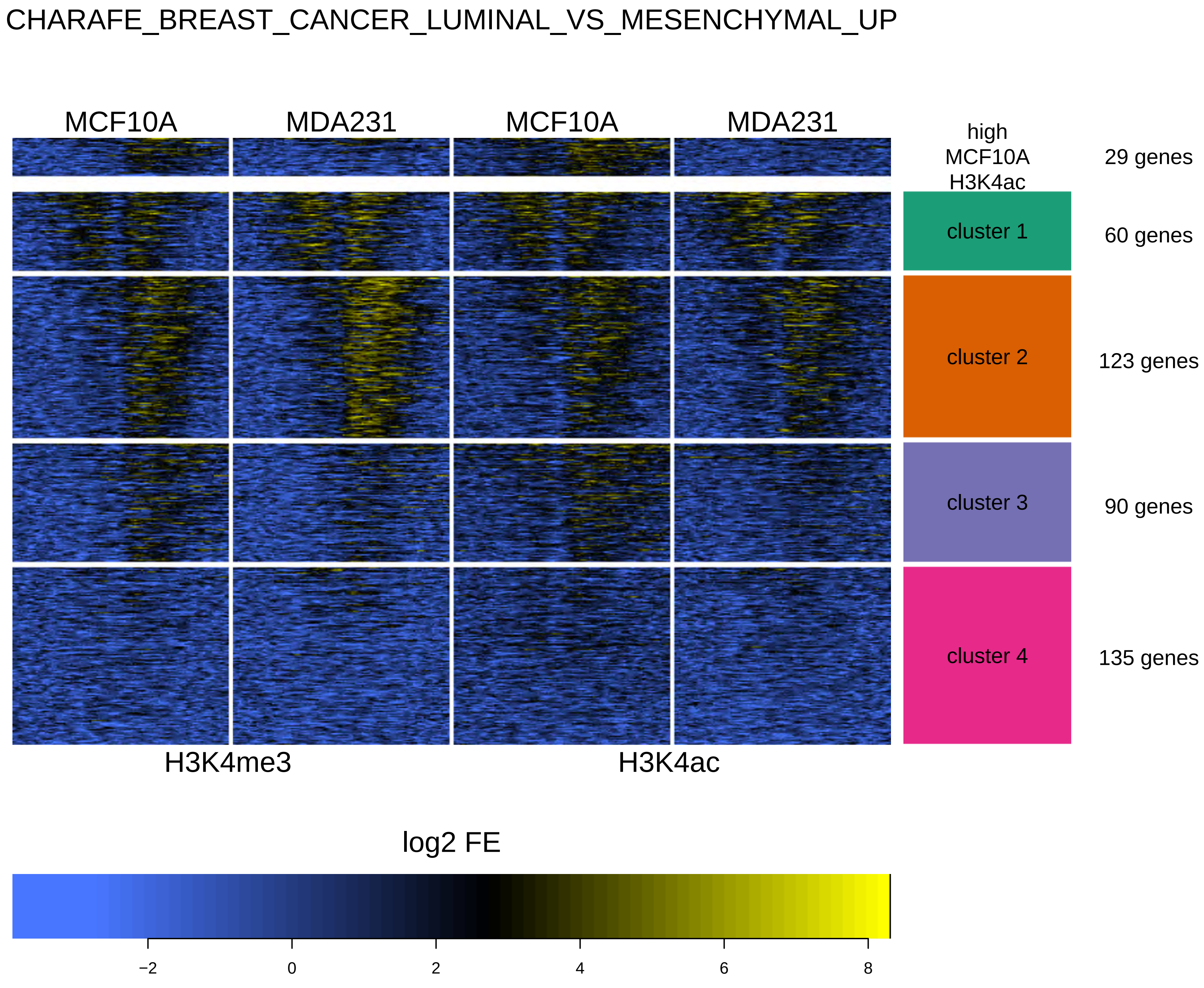
CHARAFE_BREAST_CANCER_LUMINAL_VS_MESENCHYMAL_UP
MCF10A
MDA231
MCF10A
MDA231
high
MCF10A
H3K4ac
29 genes
cluster 1
60 genes
cluster 2
123 genes
cluster 3
90 genes
cluster 4
135 genes
H3K4me3
H3K4ac
log2 FE
−2
0
2
4
6
8

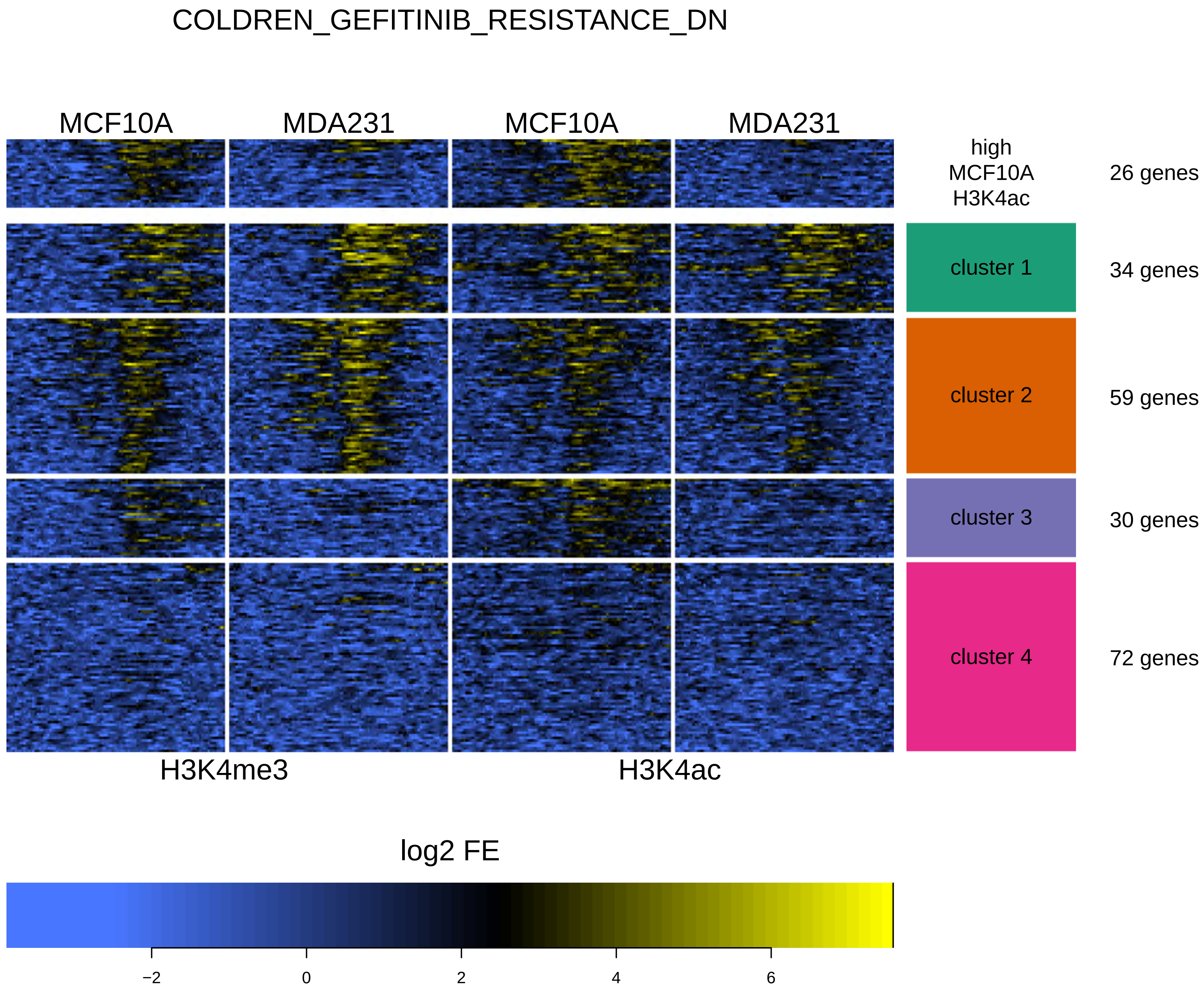
COLDREN_GEFITINIB_RESISTANCE_DN
MCF10A
MDA231
MCF10A
MDA231
high MCF10A H3K4ac
26 genes
cluster 1
34 genes
cluster 2
59 genes
cluster 3
30 genes
cluster 4
72 genes
H3K4me3
H3K4ac
log2 FE
−2
0
2
4
6

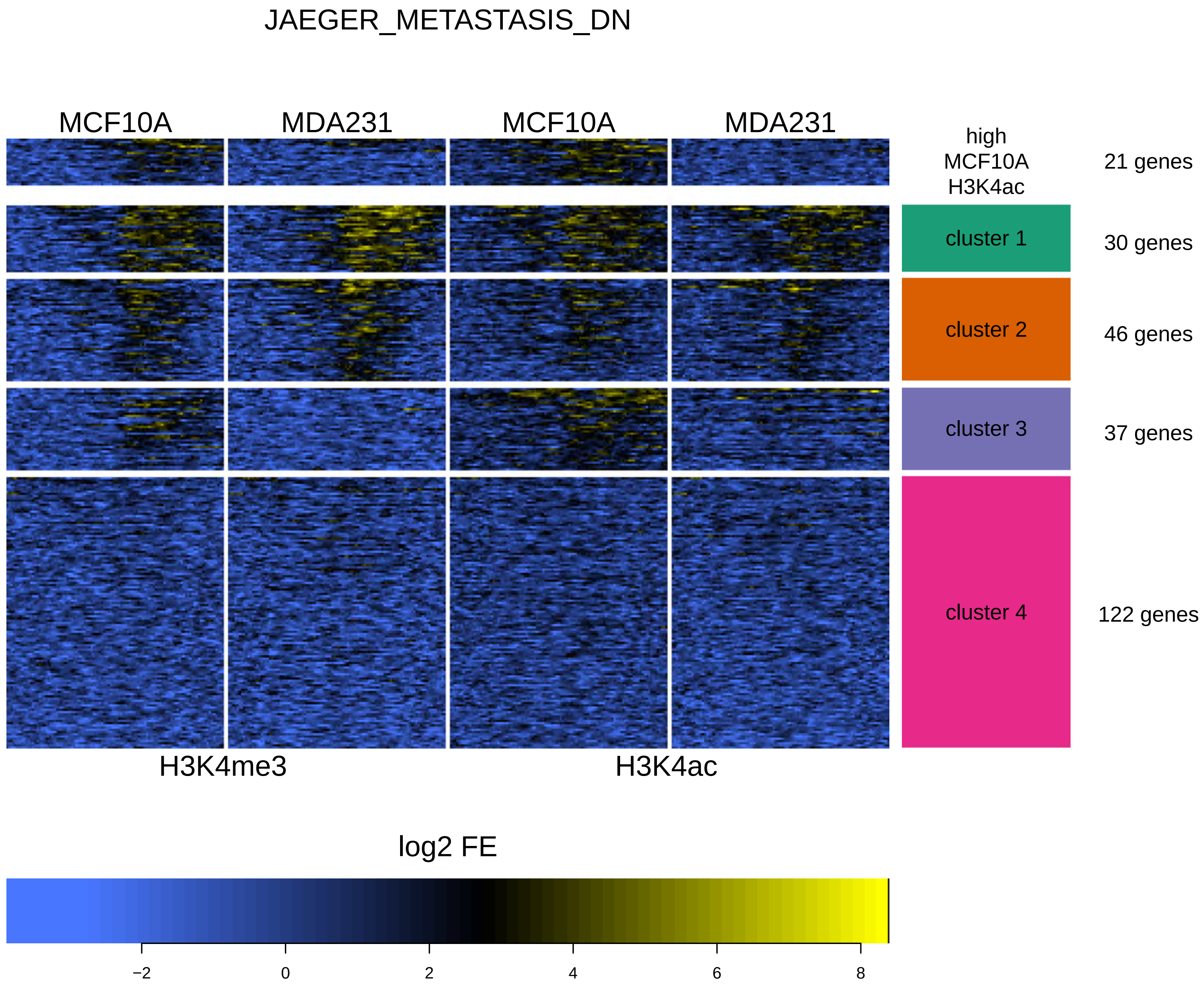

JAEGER_METASTASIS_DN
MCF10A
MDA231
MCF10A
MDA231
high
MCF10A
H3K4ac
21 genes
cluster 1
30 genes
cluster 2
46 genes
cluster 3
37 genes
cluster 4
122 genes
H3K4me3
H3K4ac
log2 FE
−2
0
2
4
6
8

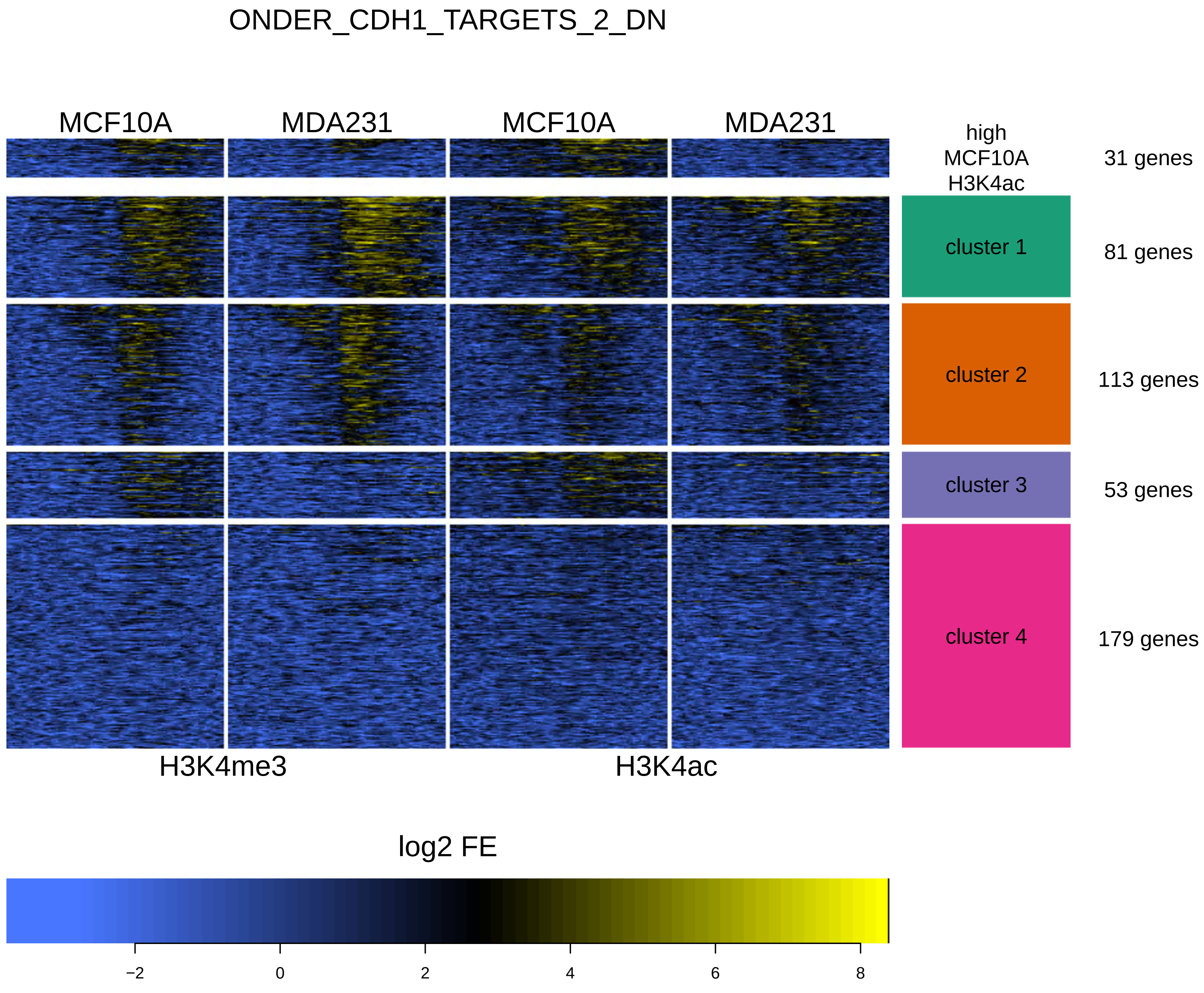

ONDER_CDH1_TARGETS_2_DN
MCF10A
MDA231
MCF10A
MDA231
high
MCF10A
H3K4ac
31 genes
cluster 1
81 genes
cluster 2
113 genes
cluster 3
53 genes
cluster 4
179 genes
H3K4me3
H3K4ac
log2 FE
−2
0
2
4
6
8

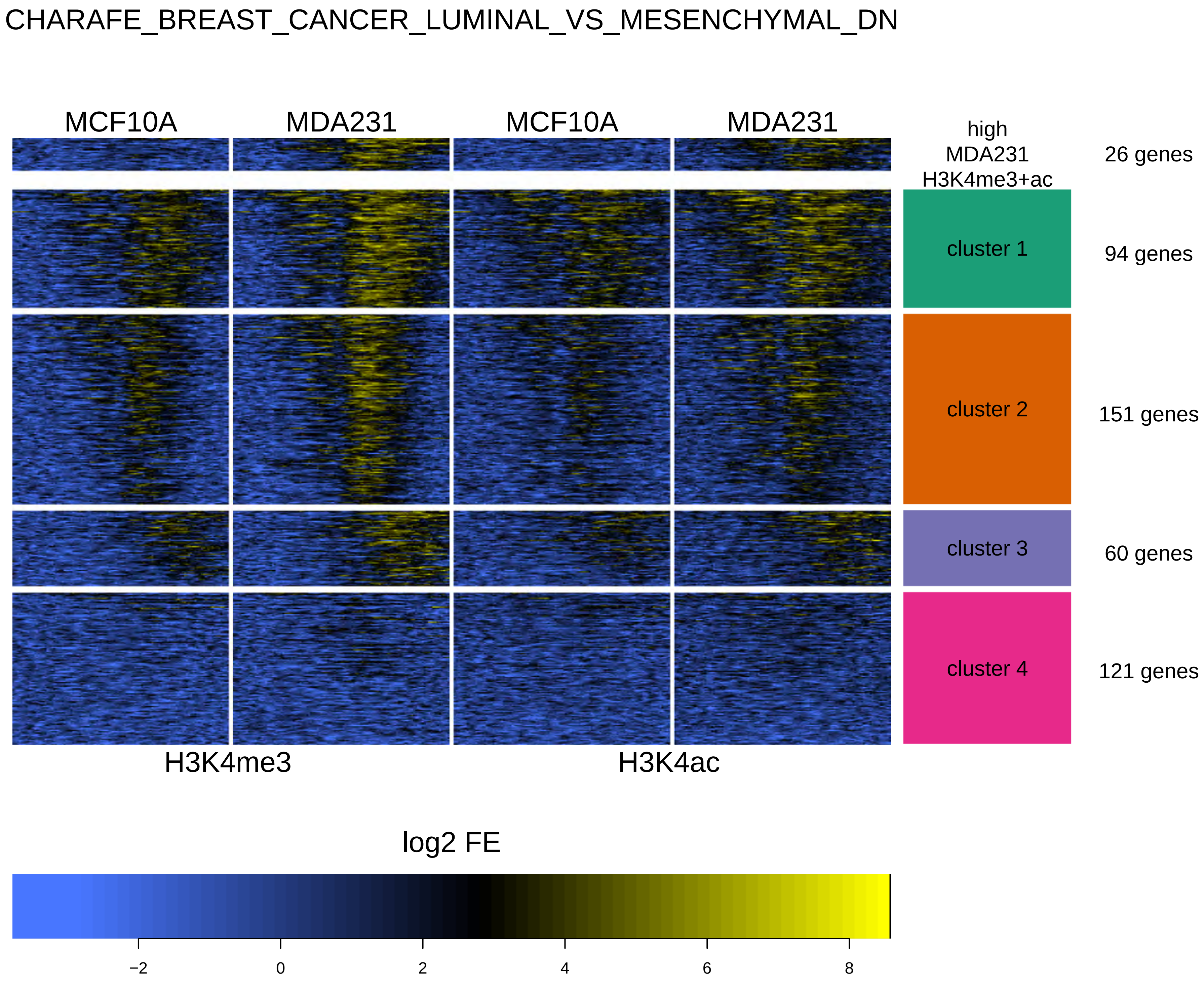
CHARAFE_BREAST_CANCER_LUMINAL_VS_MESENCHYMAL_DN
MCF10A
MDA231
MCF10A
MDA231
high
MDA231
H3K4me3+ac
26 genes
cluster 1
94 genes
cluster 2
151 genes
cluster 3
60 genes
cluster 4
121 genes
H3K4me3
H3K4ac
log2 FE
−2
0
2
4
6
8

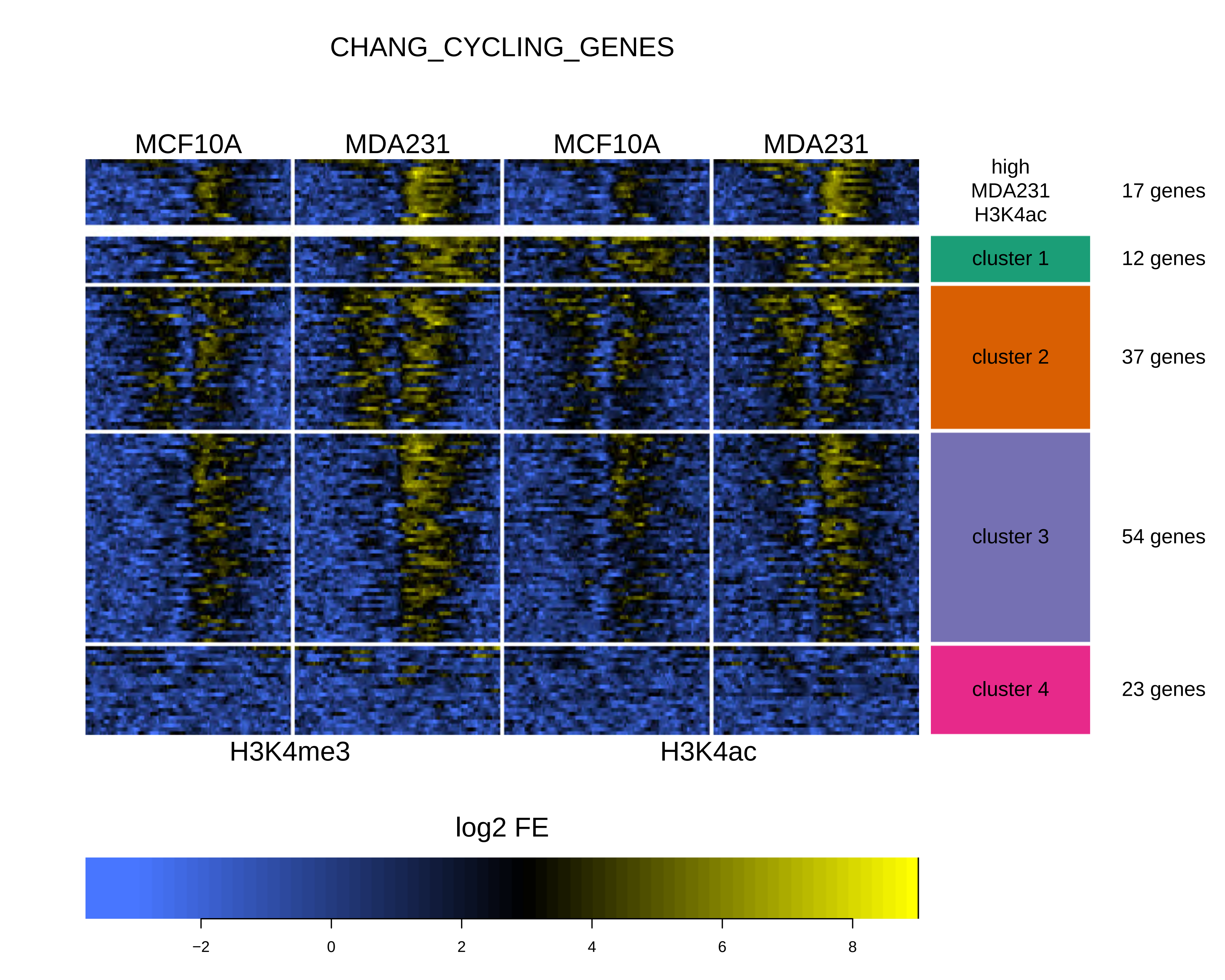

CHANG_CYCLING_GENES
MCF10A
MDA231
MCF10A
MDA231
high
MDA231
H3K4ac
17 genes
cluster 1
12 genes
cluster 2
37 genes
cluster 3
54 genes
cluster 4
23 genes
H3K4me3
H3K4ac
log2 FE
−2
0
2
4
6
8

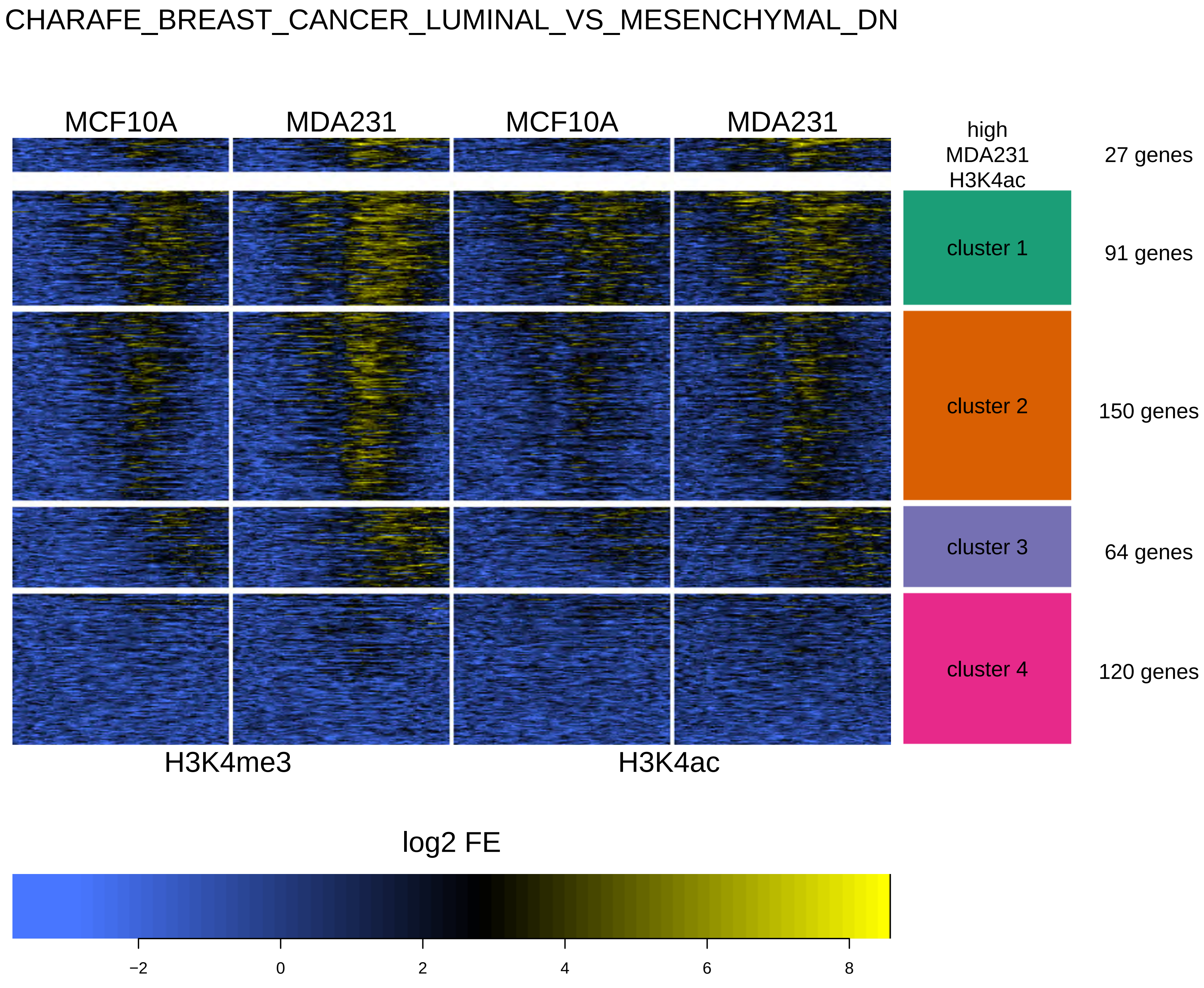
CHARAFE_BREAST_CANCER_LUMINAL_VS_MESENCHYMAL_DN
MCF10A
MDA231
MCF10A
MDA231
high MDA231 H3K4ac
27 genes
cluster 1
91 genes
cluster 2
150 genes
cluster 3
64 genes
cluster 4
120 genes
H3K4me3
H3K4ac
log2 FE
−2
0
2
4
6
8

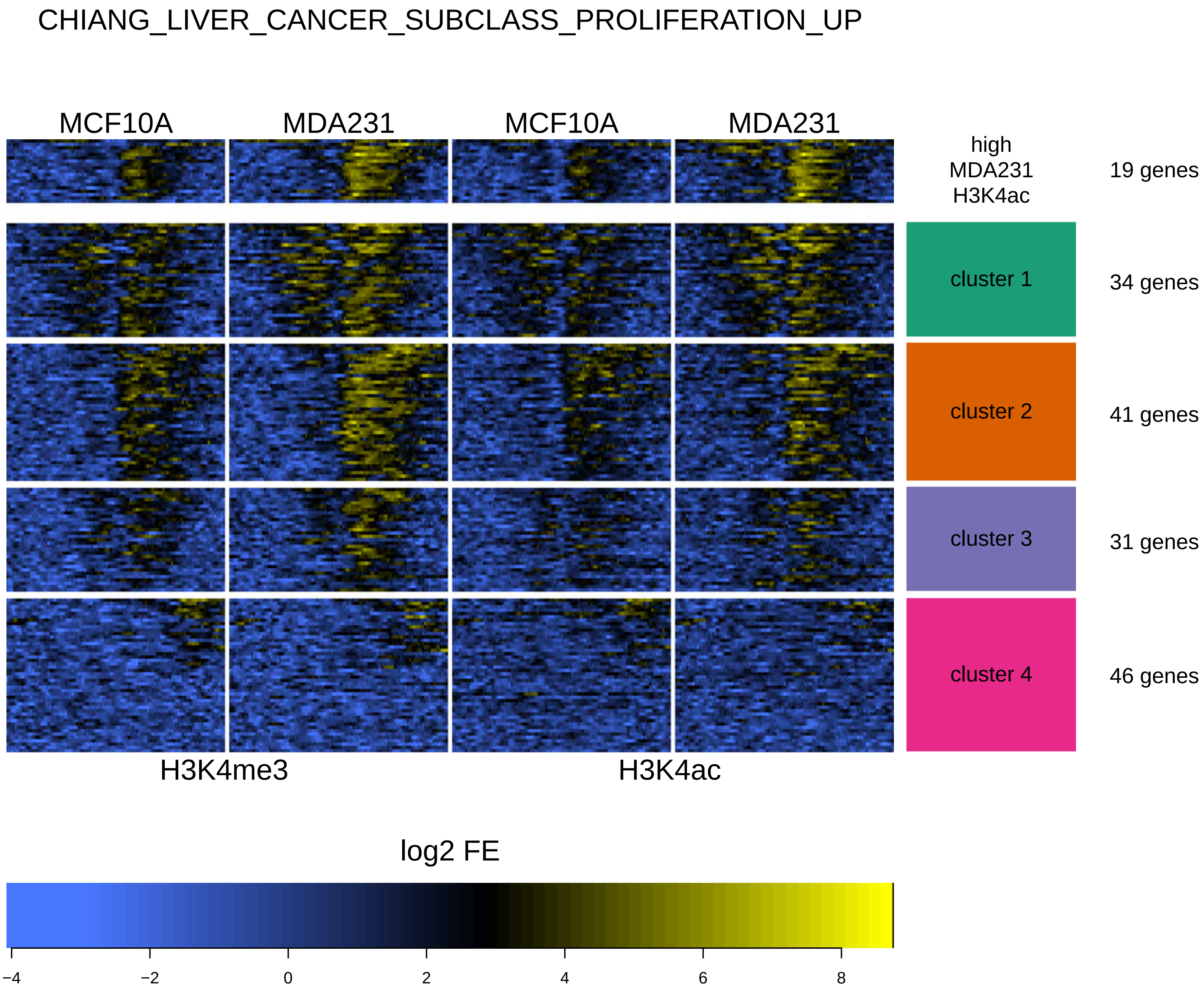
CHIANG_LIVER_CANCER_SUBCLASS_PROLIFERATION_UP
MCF10A
MDA231
MCF10A
MDA231
high
MDA231
H3K4ac
19 genes
cluster 1
34 genes
cluster 2
41 genes
cluster 3
31 genes
cluster 4
46 genes
H3K4me3
H3K4ac
log2 FE
−4
−2
0
2
4
6
8

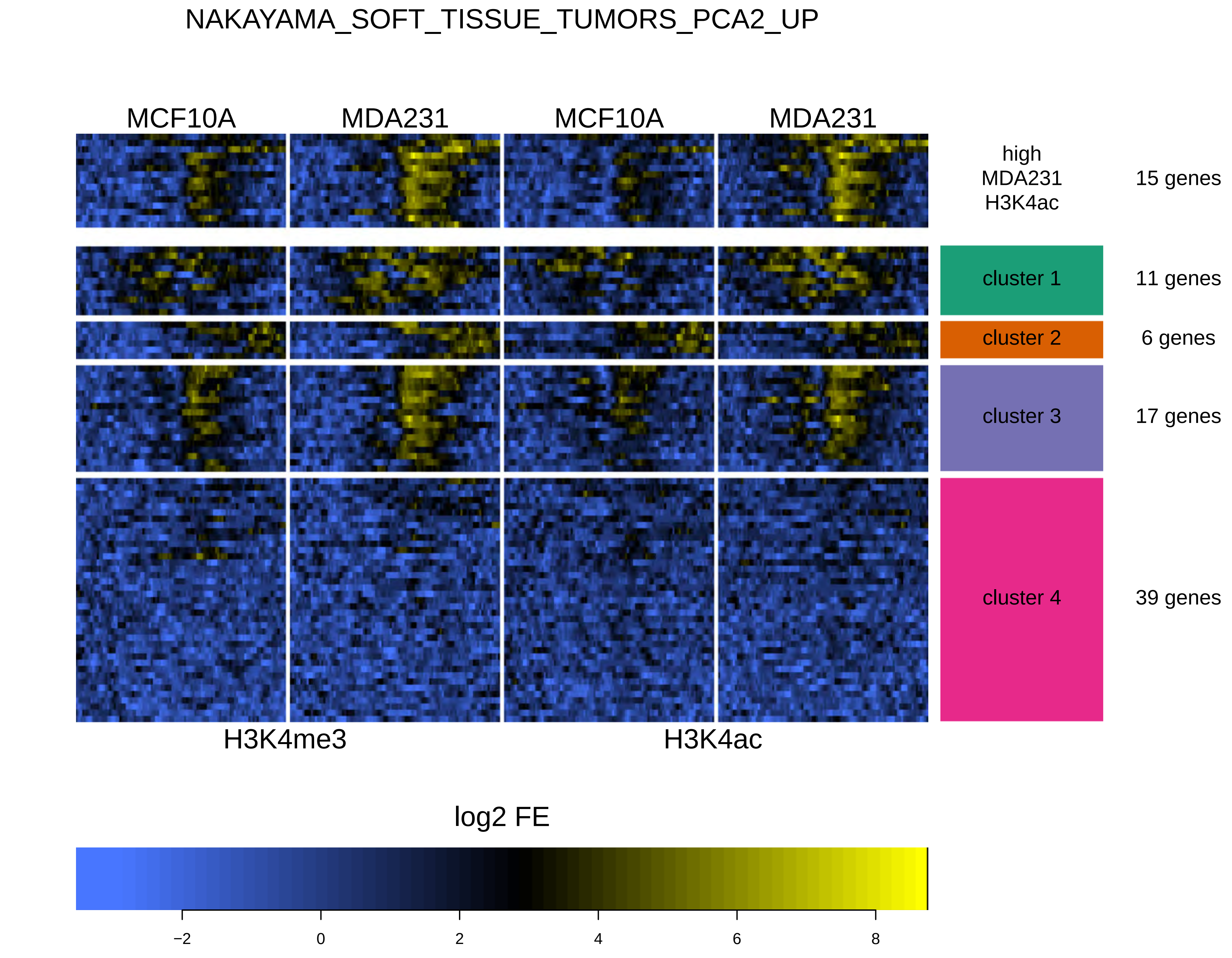

NAKAYAMA_SOFT_TISSUE_TUMORS_PCA2_UP
MCF10A
MDA231
MCF10A
MDA231
high MDA231 H3K4ac
15 genes
cluster 1
11 genes
cluster 2
6 genes
cluster 3
17 genes
cluster 4
39 genes
H3K4me3
H3K4ac
log2 FE
−2
0
2
4
6
8

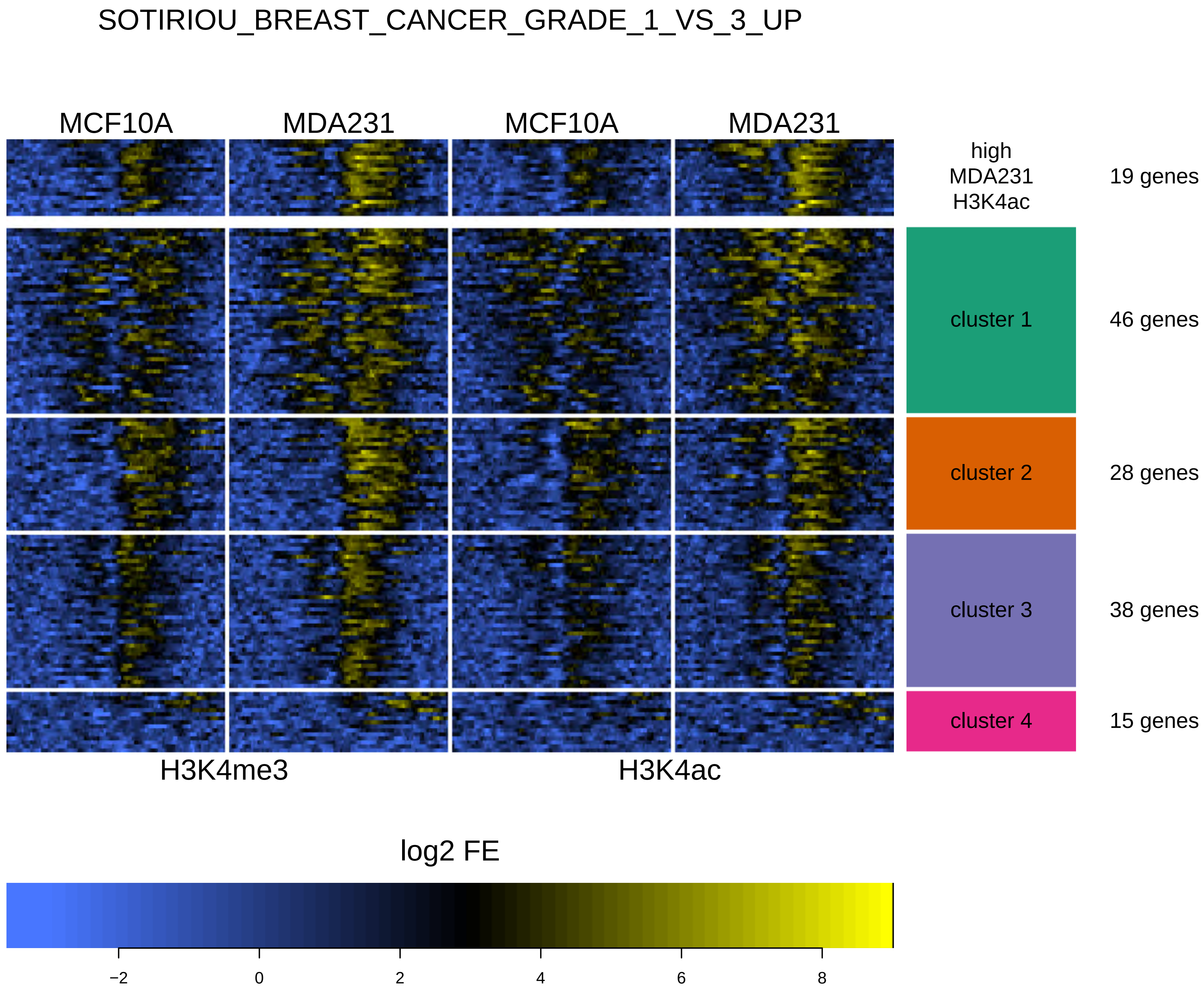
SOTIRIOU_BREAST_CANCER_GRADE_1_VS_3_UP
MCF10A
MDA231
MCF10A
MDA231
high
MDA231
H3K4ac
19 genes
cluster 1
46 genes
cluster 2
28 genes
cluster 3
38 genes
cluster 4
15 genes
H3K4me3
H3K4ac
log2 FE
−2
0
2
4
6
8

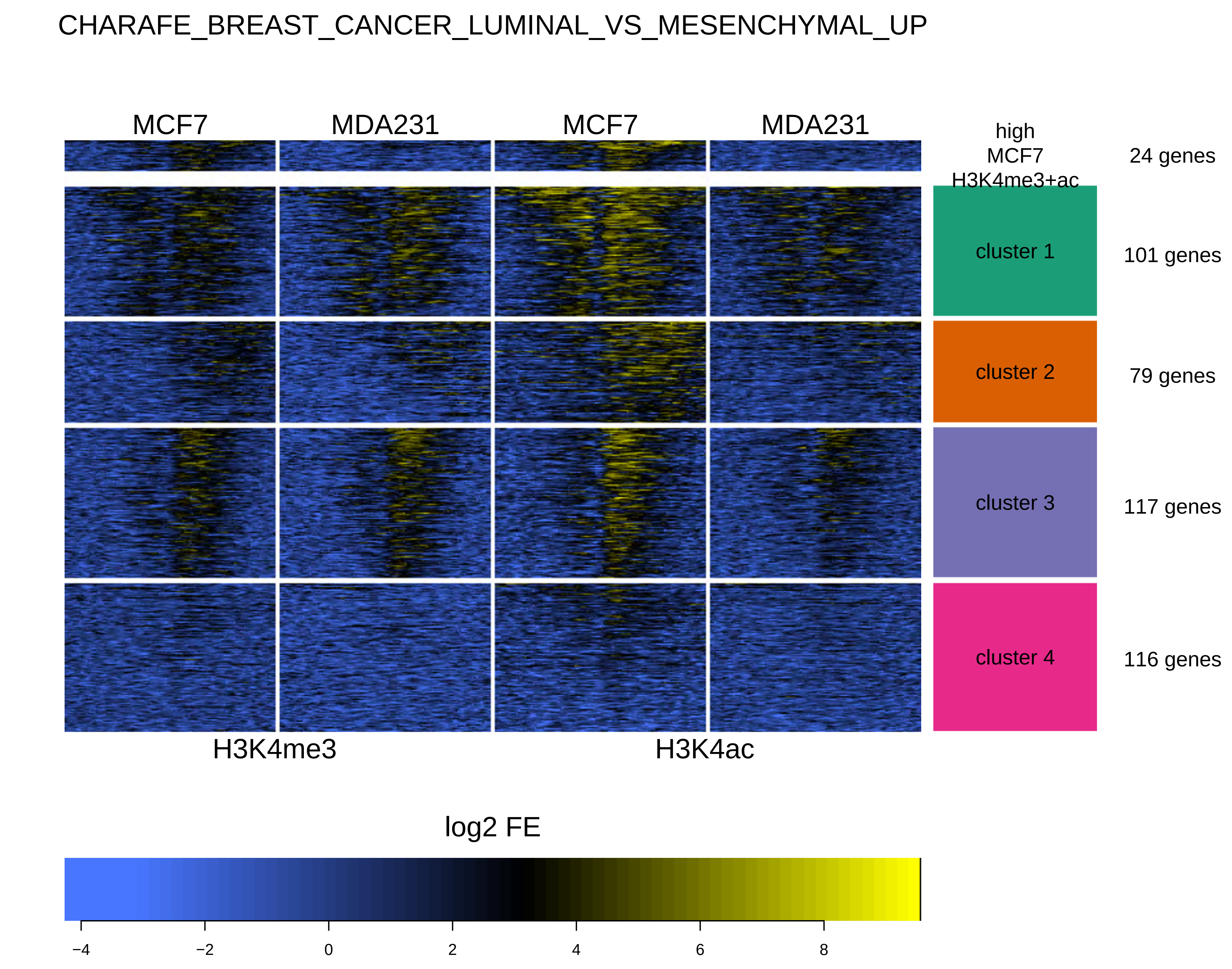
CHARAFE_BREAST_CANCER_LUMINAL_VS_MESENCHYMAL_UP
MCF7
MDA231
MCF7
MDA231
high MCF7 H3K4me3+ac
24 genes
cluster 1
101 genes
cluster 2
79 genes
cluster 3
117 genes
cluster 4
116 genes
H3K4me3
H3K4ac
log2 FE
−4
−2
0
2
4
6
8

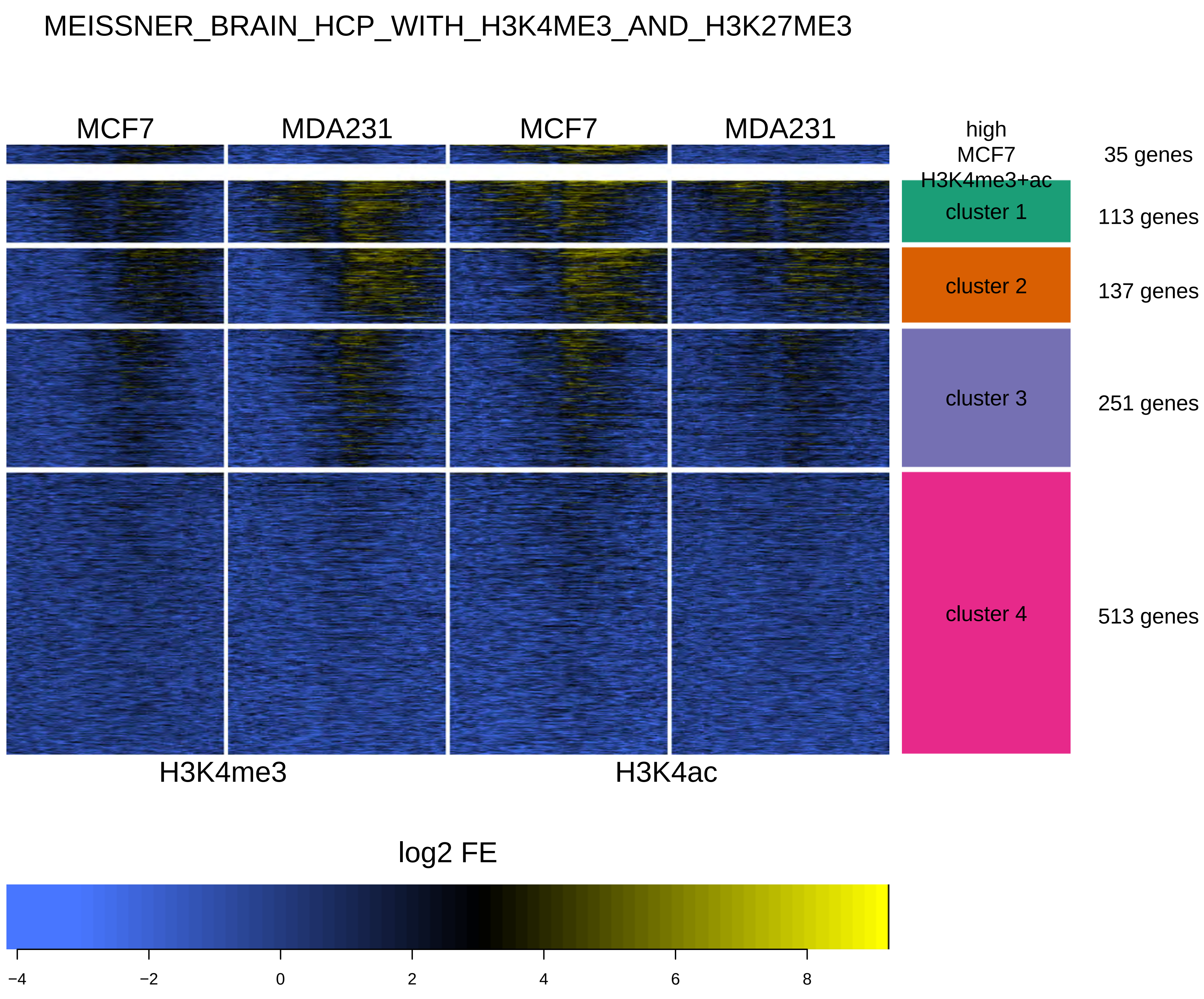
MEISSNER_BRAIN_HCP_WITH_H3K4ME3_AND_H3K27ME3
MCF7
MDA231
MCF7
MDA231
high
MCF7
H3K4me3+ac
35 genes
cluster 1
113 genes
cluster 2
137 genes
cluster 3
251 genes
cluster 4
513 genes
H3K4me3
H3K4ac
log2 FE
−4
−2
0
2
4
6
8

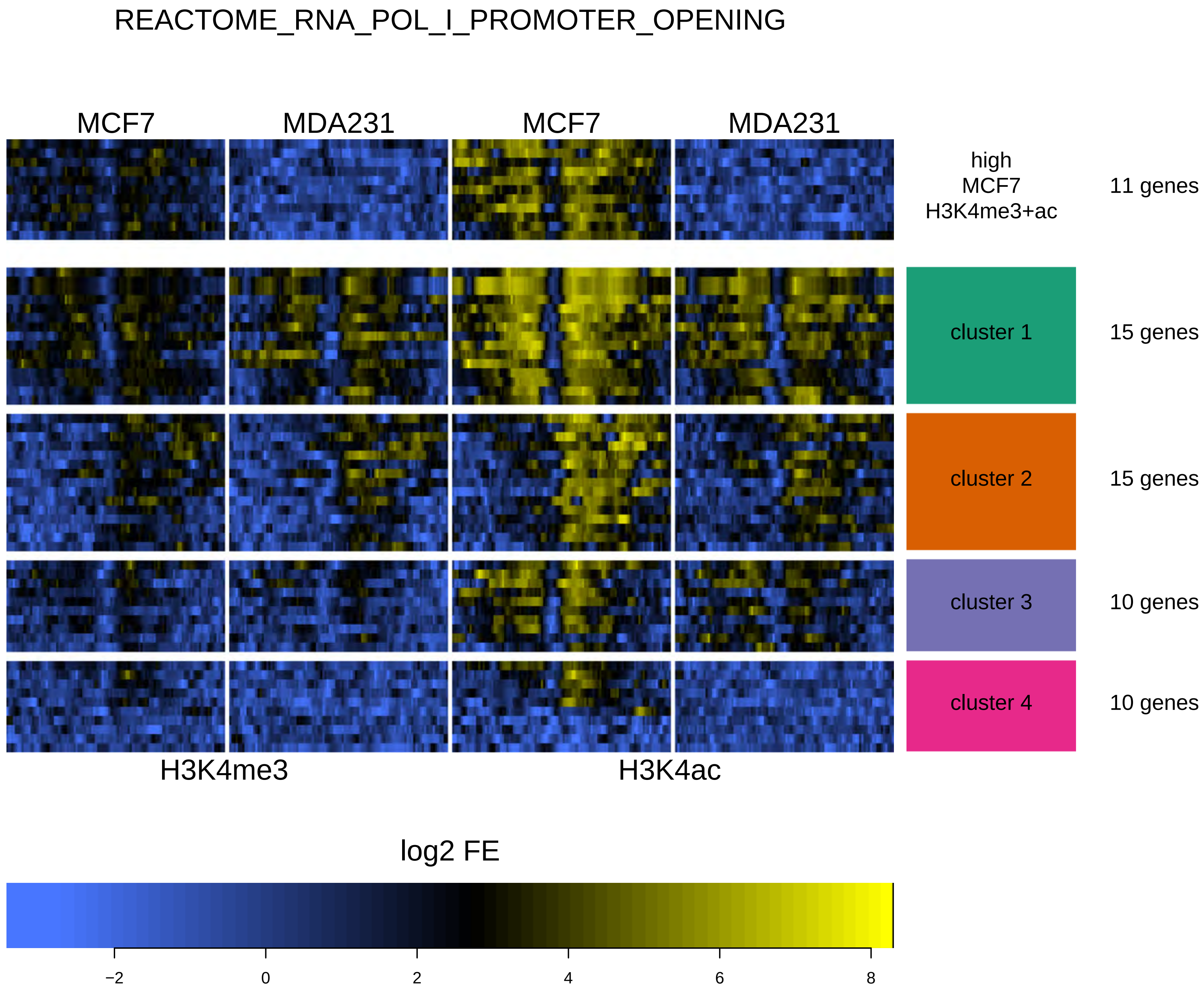

REACTOME_RNA_POL_I_PROMOTER_OPENING
MCF7
MDA231
MCF7
MDA231
high
MCF7
H3K4me3+ac
11 genes
cluster 1
15 genes
cluster 2
15 genes
cluster 3
10 genes
cluster 4
10 genes
H3K4me3
H3K4ac
log2 FE
−2
0
2
4
6
8

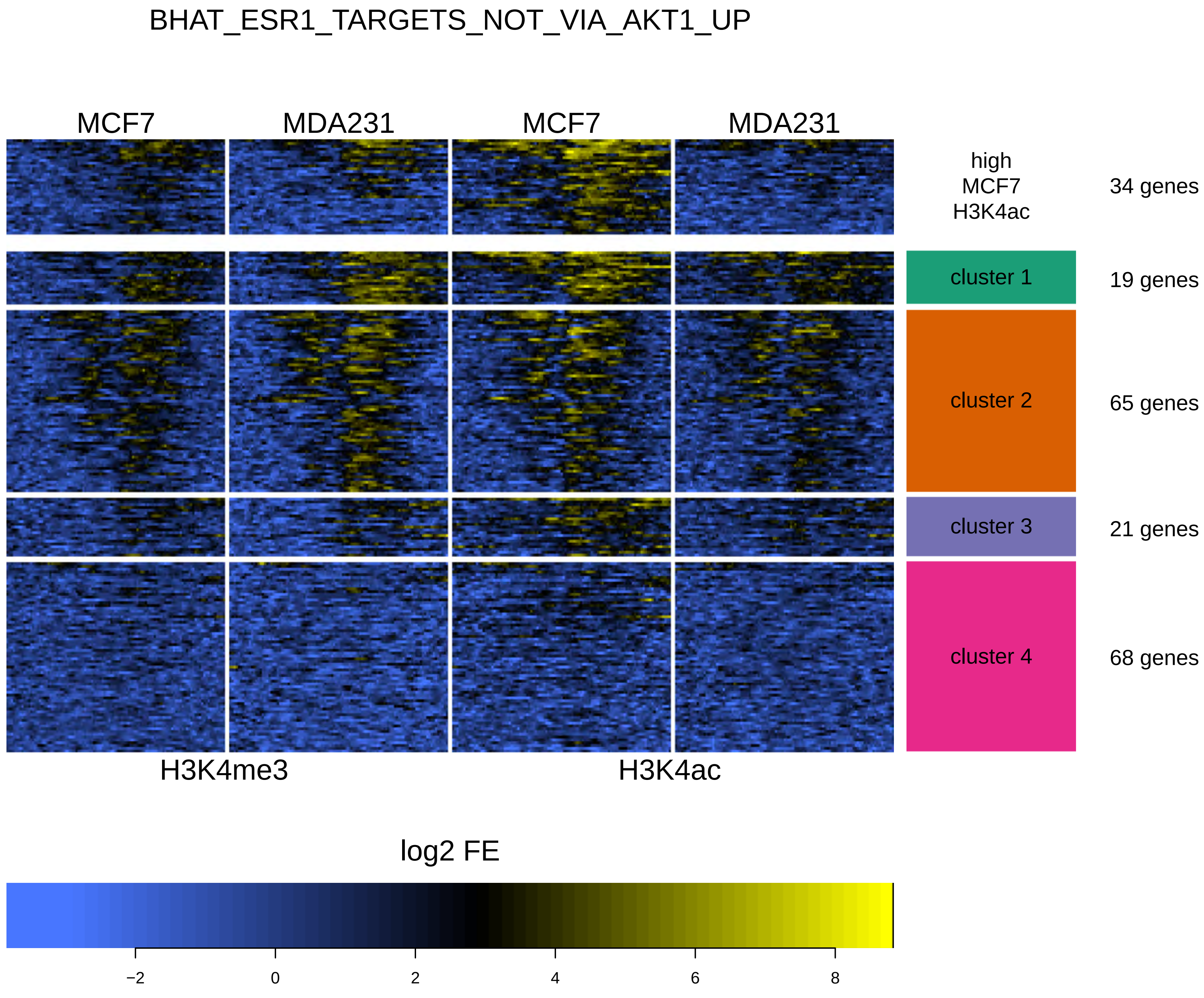
BHAT_ESR1_TARGETS_NOT_VIA_AKT1_UP
MCF7
MDA231
MCF7
MDA231
high MCF7 H3K4ac
34 genes
cluster 1
19 genes
cluster 2
65 genes
cluster 3
21 genes
cluster 4
68 genes
H3K4me3
H3K4ac
log2 FE
−2
0
2
4
6
8

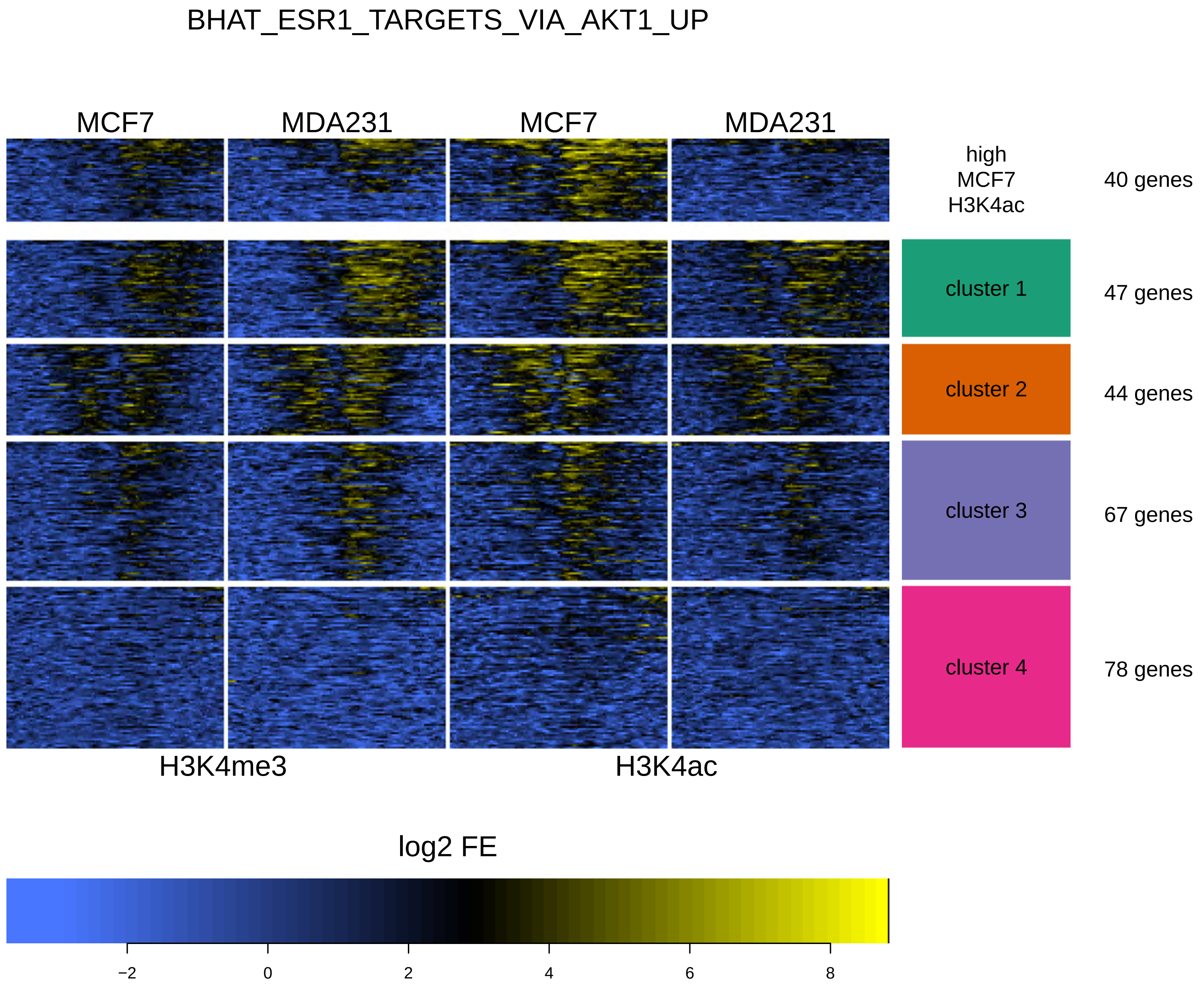
BHAT_ESR1_TARGETS_VIA_AKT1_UP
MCF7
MDA231
MCF7
MDA231
high
MCF7
H3K4ac
40 genes
cluster 1
47 genes
cluster 2
44 genes
cluster 3
67 genes
cluster 4
78 genes
H3K4me3
H3K4ac
log2 FE
−2
0
2
4
6
8

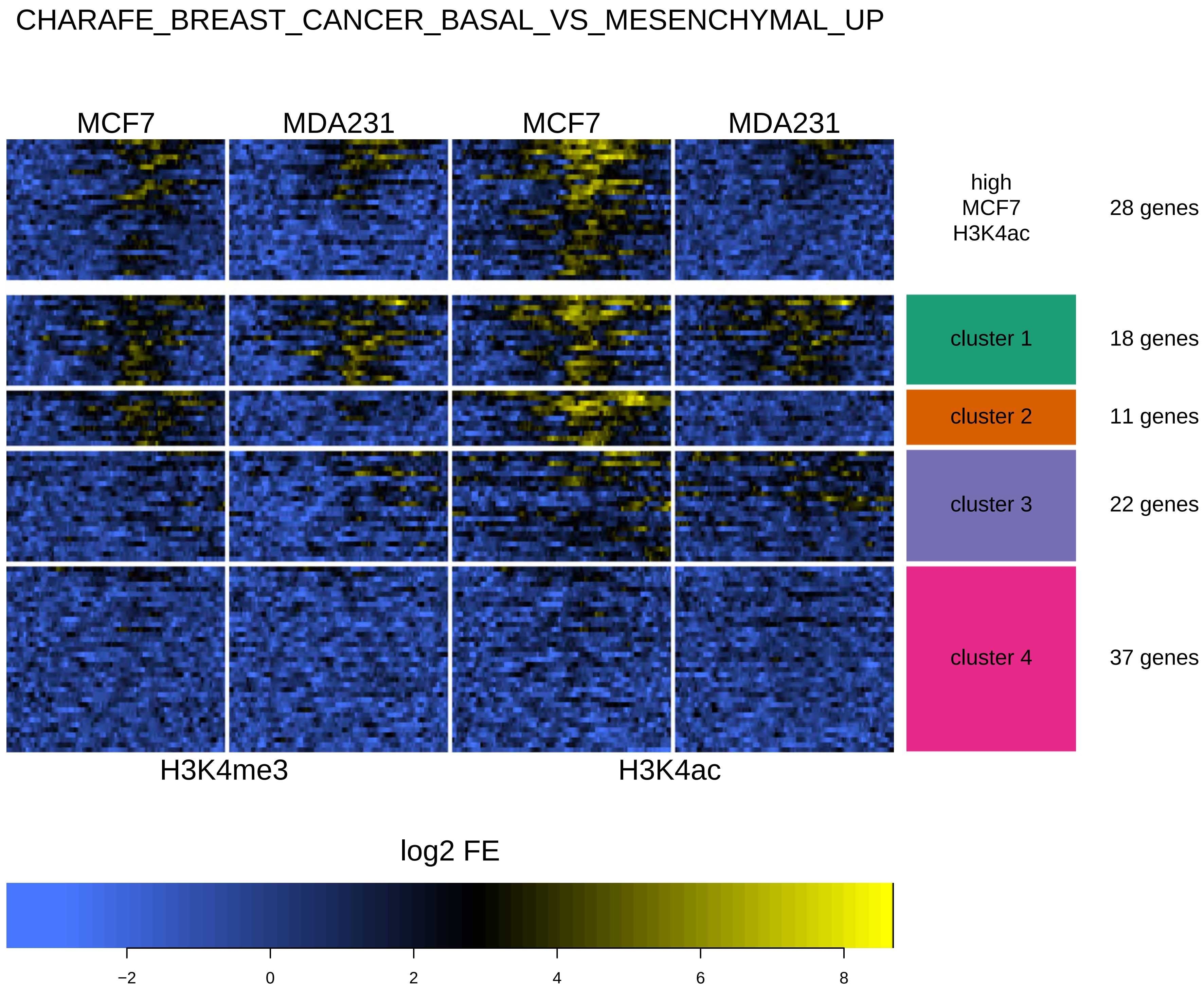

CHARAFE_BREAST_CANCER_BASAL_VS_MESENCHYMAL_UP
MCF7
MDA231
MCF7
MDA231
high
MCF7
H3K4ac
28 genes
cluster 1
18 genes
cluster 2
11 genes
cluster 3
22 genes
cluster 4
37 genes
H3K4me3
H3K4ac
log2 FE
−2
0
2
4
6
8

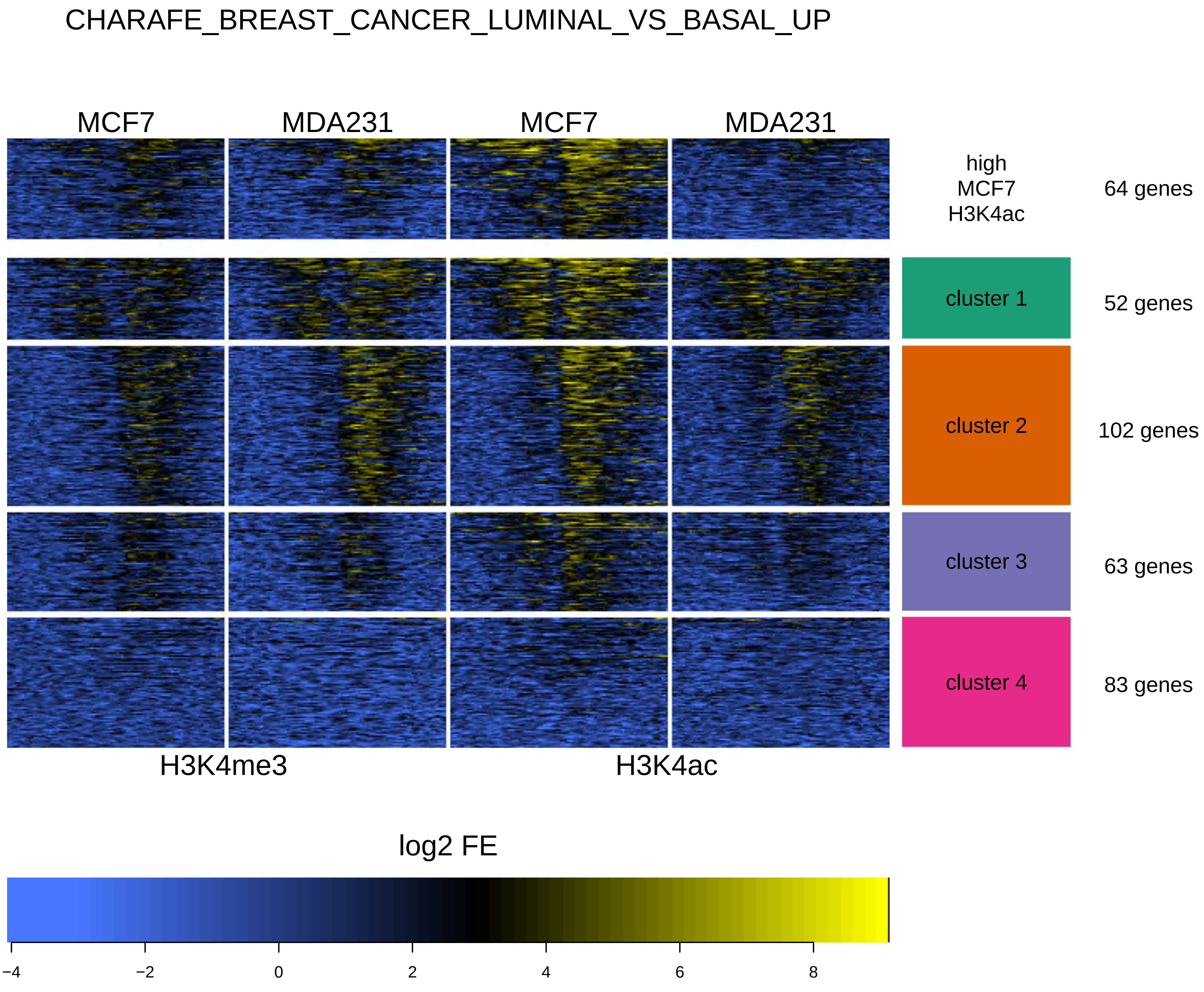
CHARAFE_BREAST_CANCER_LUMINAL_VS_BASAL_UP
MCF7
MDA231
MCF7
MDA231
high
MCF7
H3K4ac
64 genes
cluster 1
52 genes
cluster 2
102 genes
cluster 3
63 genes
cluster 4
83 genes
H3K4me3
H3K4ac
log2 FE
−4
−2
0
2
4
6
8

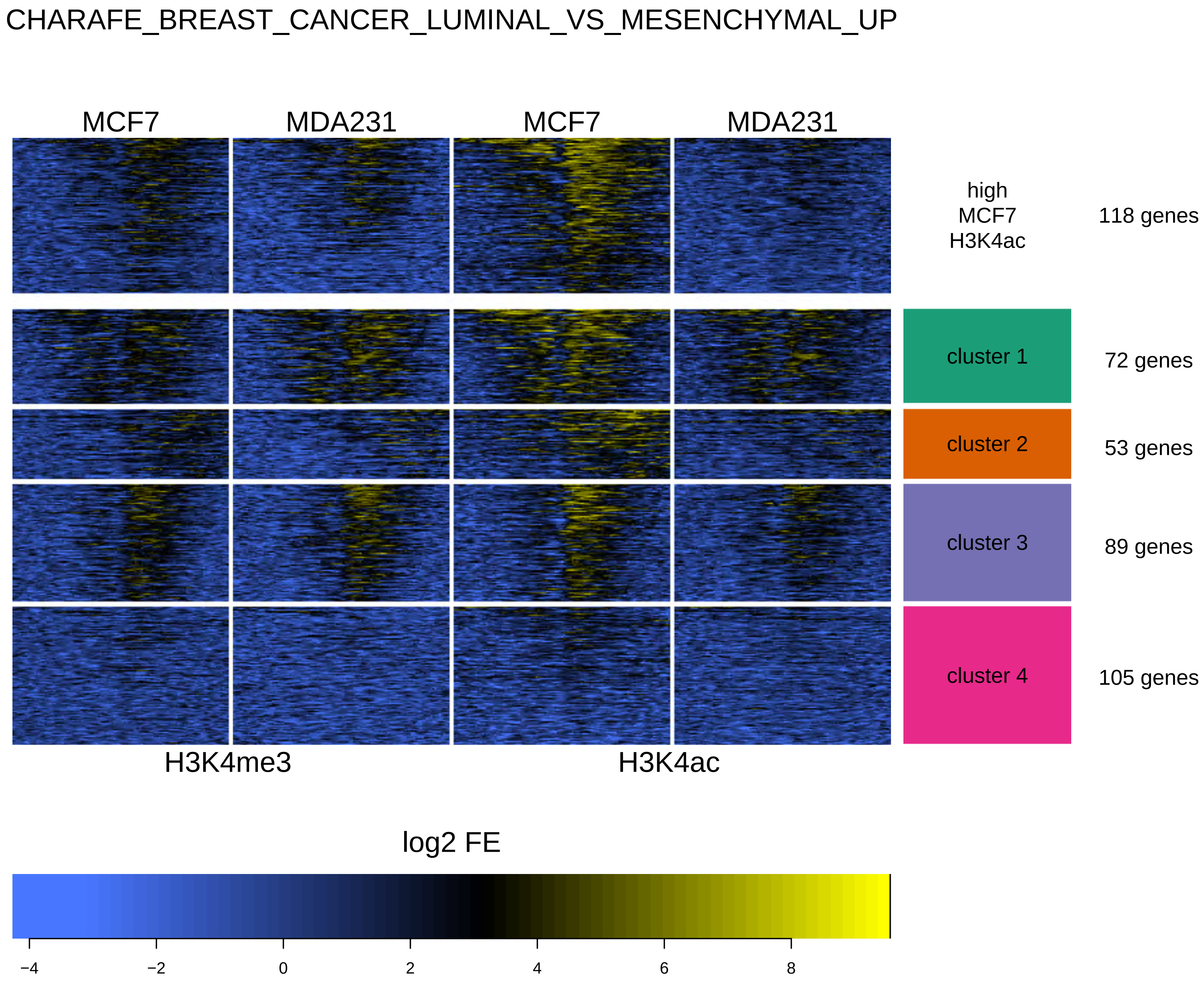

CHARAFE_BREAST_CANCER_LUMINAL_VS_MESENCHYMAL_UP
MCF7
MDA231
MCF7
MDA231
high
MCF7
H3K4ac
118 genes
cluster 1
72 genes
cluster 2
53 genes
cluster 3
89 genes
cluster 4
105 genes
H3K4me3
H3K4ac
log2 FE
−4
−2
0
2
4
6
8

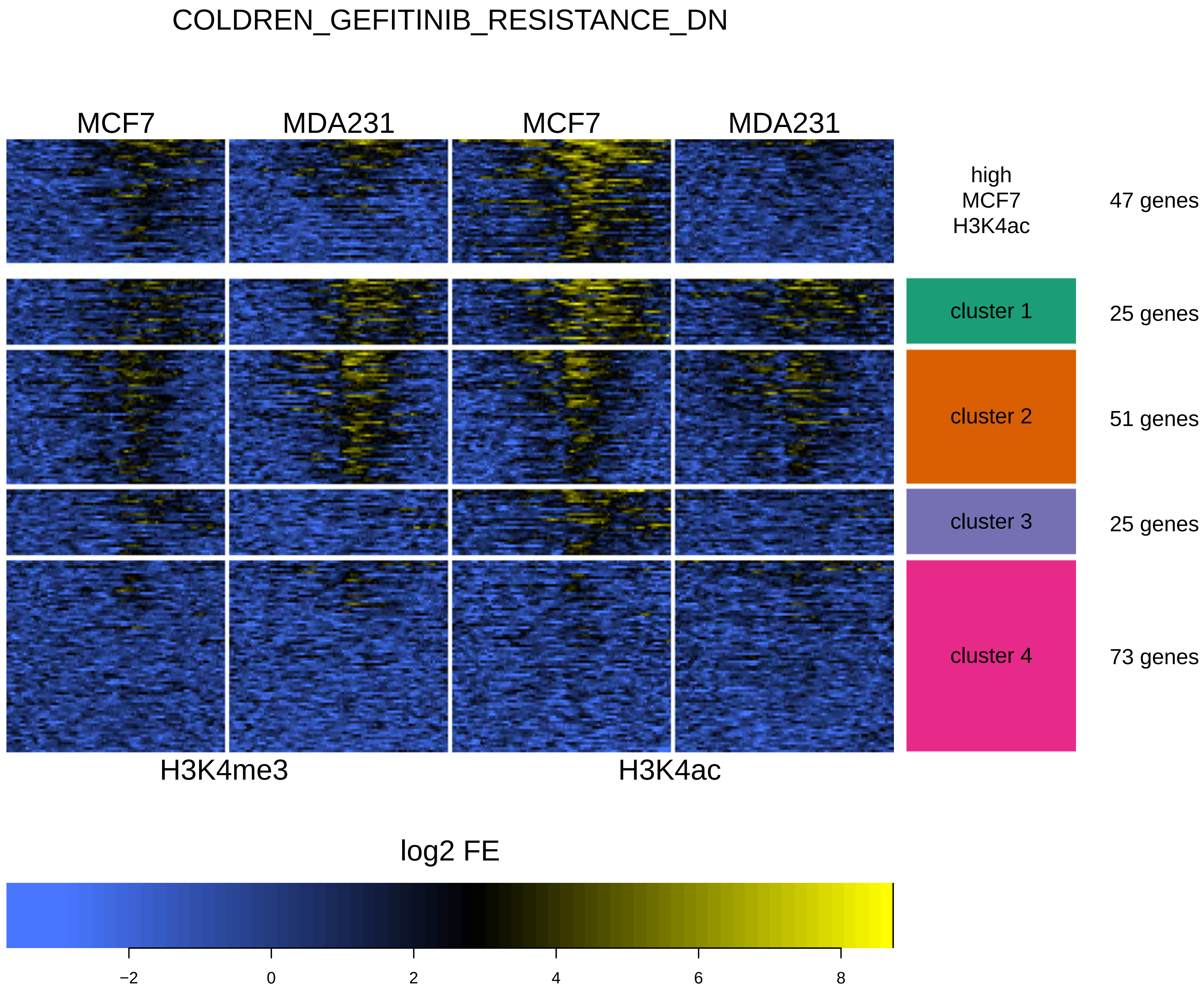
COLDREN_GEFITINIB_RESISTANCE_DN
MCF7
MDA231
MCF7
MDA231
high
MCF7
H3K4ac
47 genes
cluster 1
25 genes
cluster 2
51 genes
cluster 3
25 genes
cluster 4
73 genes
H3K4me3
H3K4ac
log2 FE
−2
0
2
4
6
8

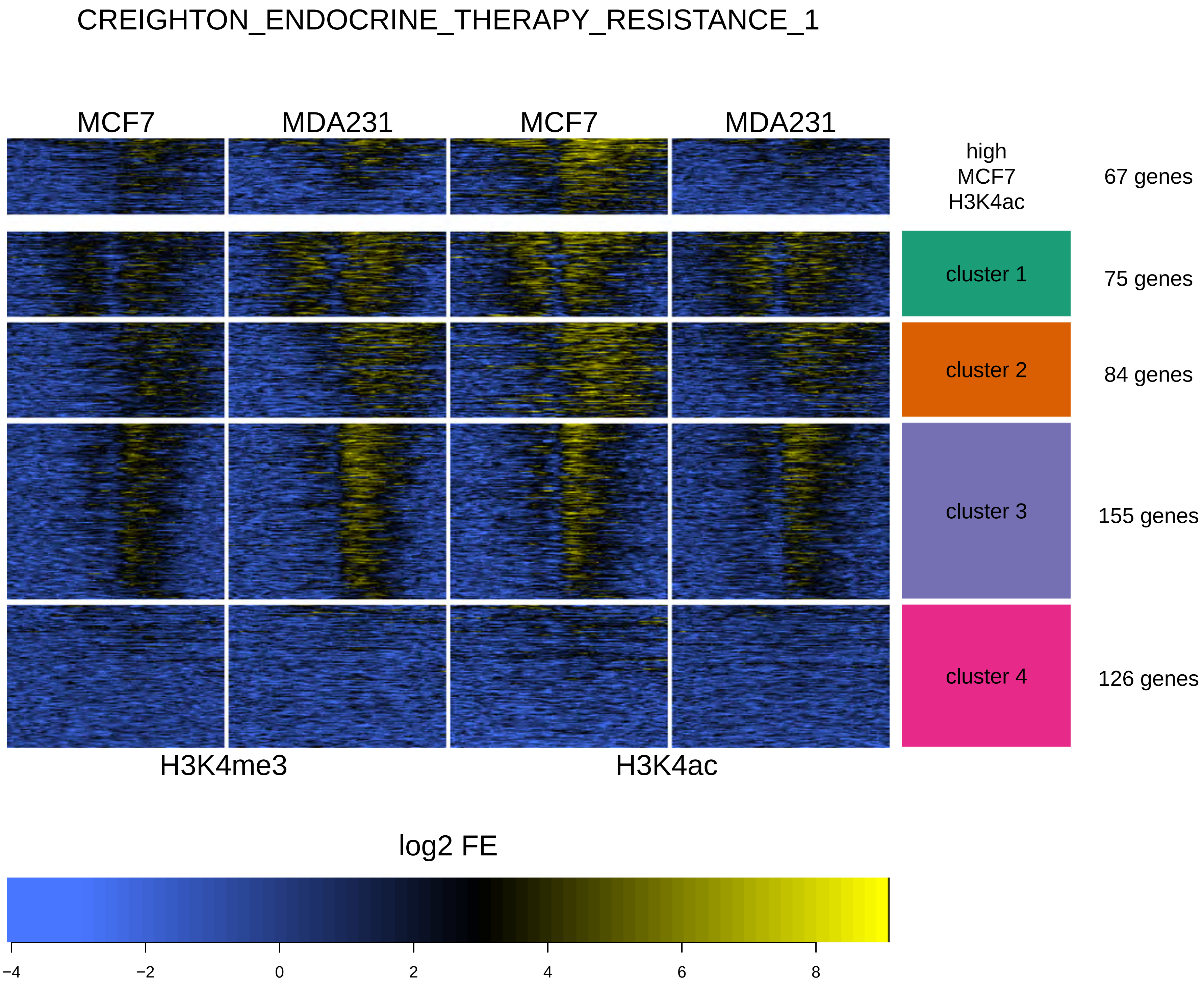
CREIGHTON_ENDOCRINE_THERAPY_RESISTANCE_1
MCF7
MDA231
MCF7
MDA231
high MCF7 H3K4ac
67 genes
cluster 1
75 genes
cluster 2
84 genes
cluster 3
155 genes
cluster 4
126 genes
H3K4me3
H3K4ac
log2 FE
−4
−2
0
2
4
6
8

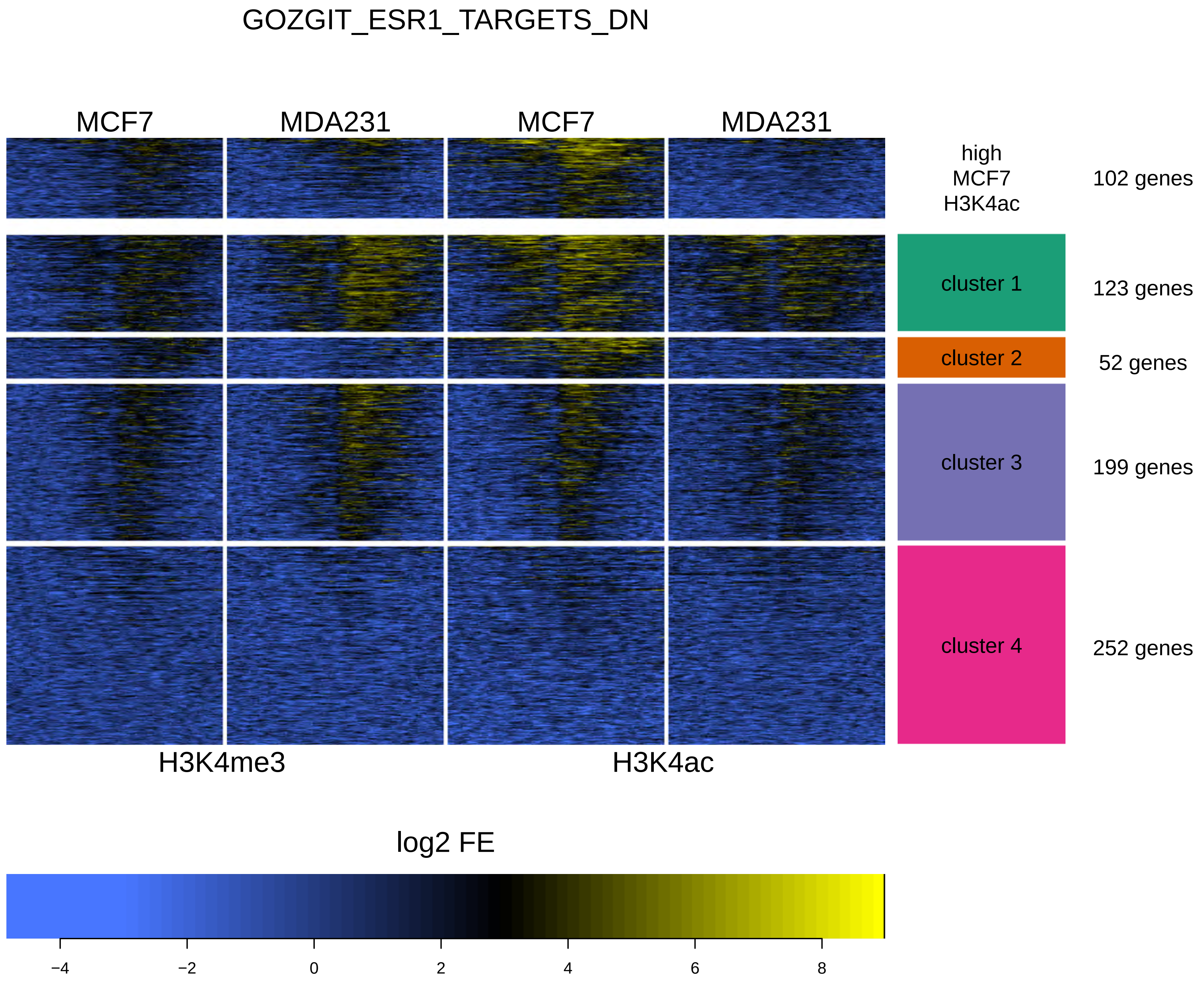
GOZGIT_ESR1_TARGETS_DN
MCF7
MDA231
MCF7
MDA231
high
MCF7
H3K4ac
102 genes
cluster 1
123 genes
cluster 2
52 genes
cluster 3
199 genes
cluster 4
252 genes
H3K4me3
H3K4ac
log2 FE
−4
−2
0
2
4
6
8

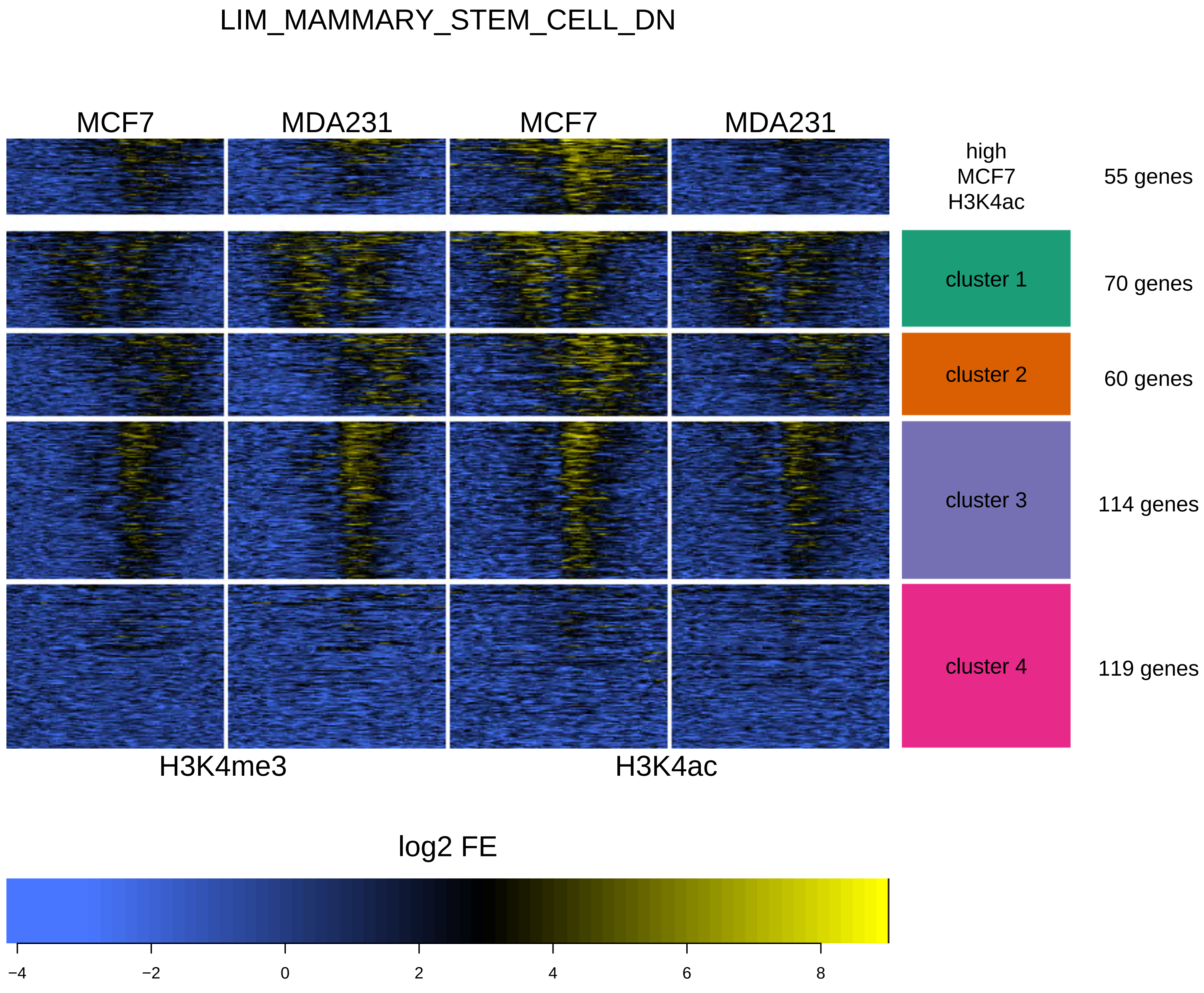
LIM_MAMMARY_STEM_CELL_DN
MCF7
MDA231
MCF7
MDA231
high MCF7 H3K4ac
55 genes
cluster 1
70 genes
cluster 2
60 genes
cluster 3
114 genes
cluster 4
119 genes
H3K4me3
H3K4ac
log2 FE
−4
−2
0
2
4
6
8

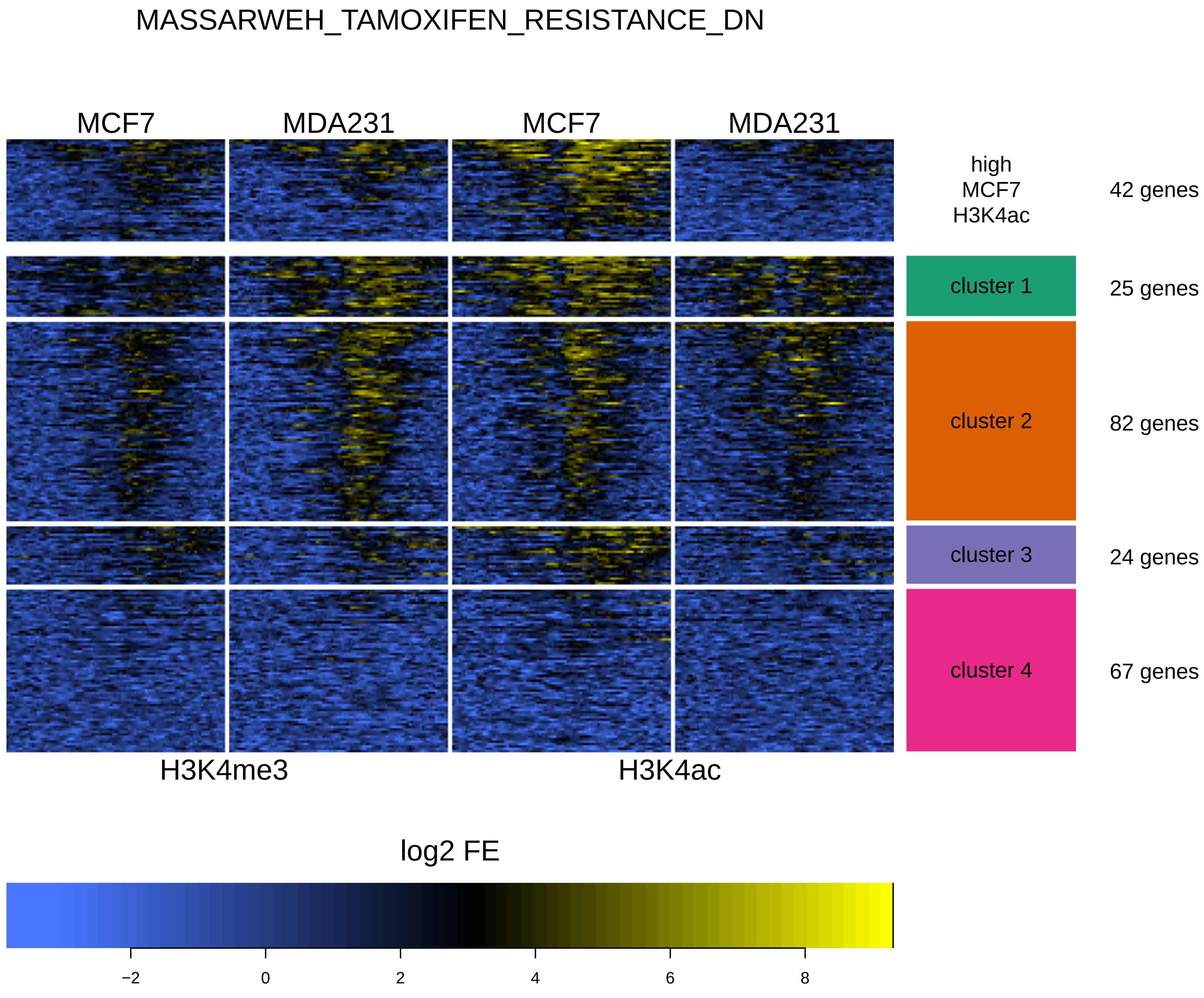
MASSARWEH_TAMOXIFEN_RESISTANCE_DN
MCF7
MDA231
MCF7
MDA231
high MCF7 H3K4ac
42 genes
cluster 1
25 genes
cluster 2
82 genes
cluster 3
24 genes
cluster 4
67 genes
H3K4me3
H3K4ac
log2 FE
−2
0
2
4
6
8

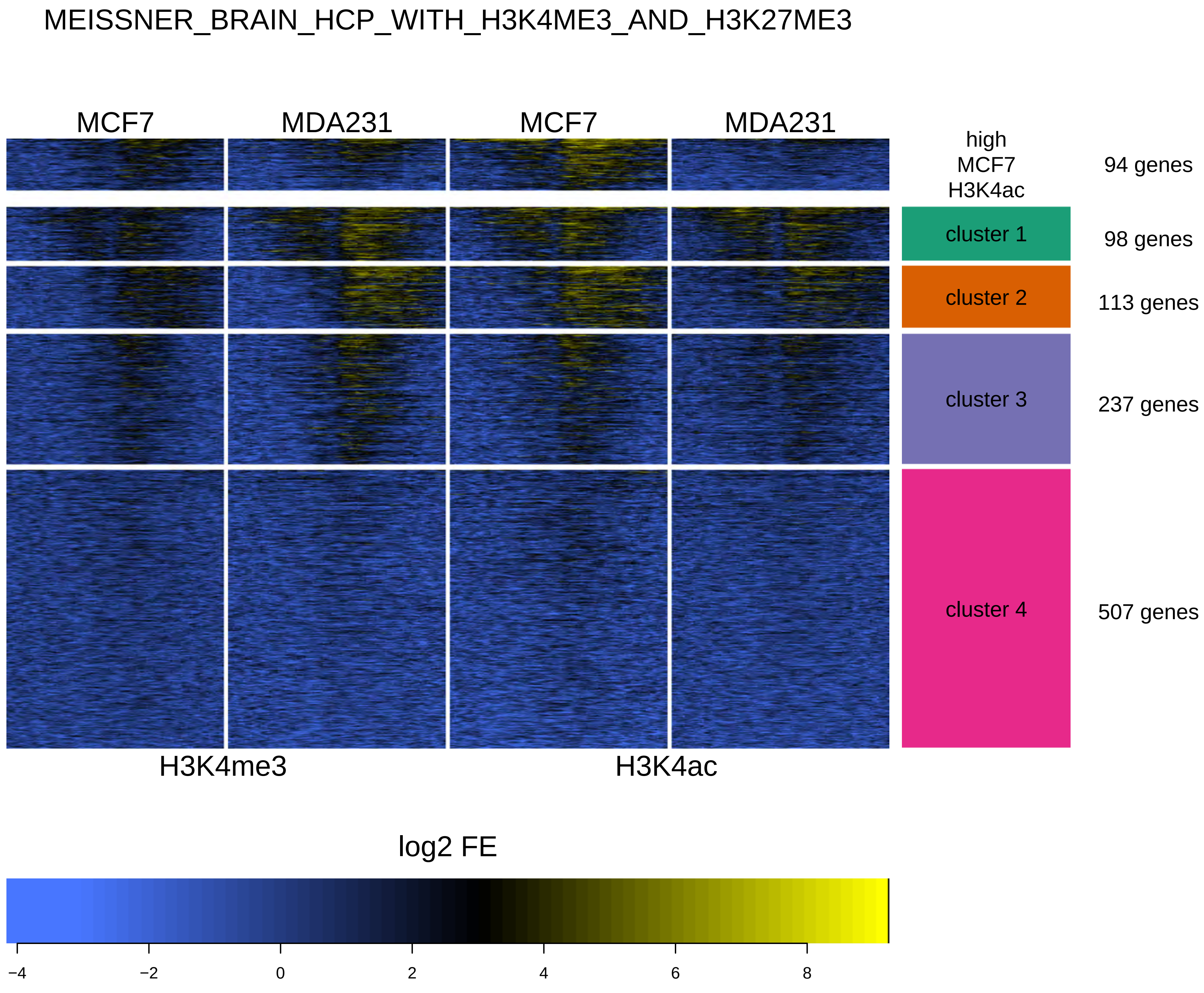
MEISSNER_BRAIN_HCP_WITH_H3K4ME3_AND_H3K27ME3
MCF7
MDA231
MCF7
MDA231
high
MCF7
H3K4ac
94 genes
cluster 1
98 genes
cluster 2
113 genes
cluster 3
237 genes
cluster 4
507 genes
H3K4me3
H3K4ac
log2 FE
−4
−2
0
2
4
6
8

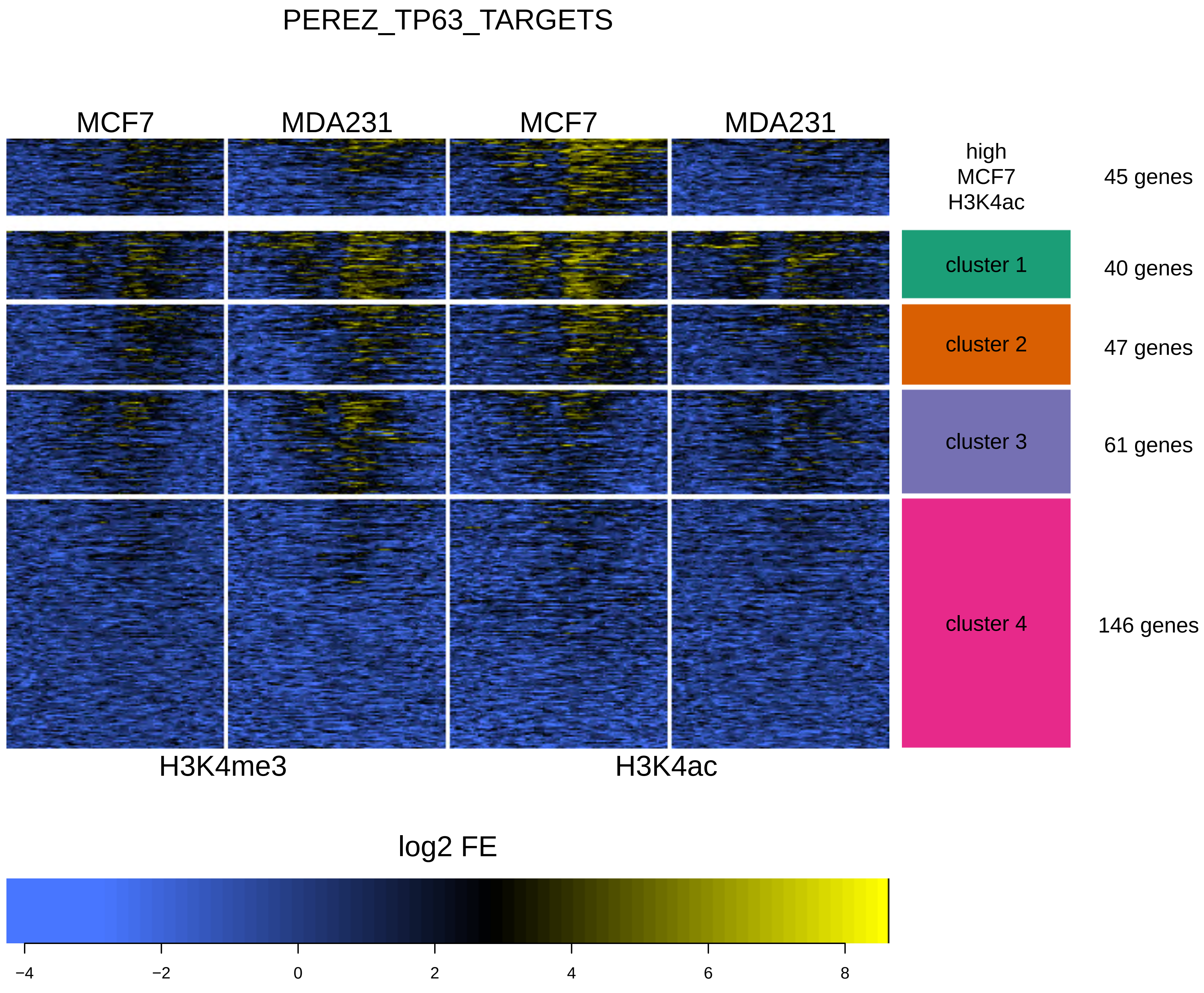
PEREZ_TP63_TARGETS
MCF7
MDA231
MCF7
MDA231
high
MCF7
H3K4ac
45 genes
cluster 1
40 genes
cluster 2
47 genes
cluster 3
61 genes
cluster 4
146 genes
H3K4me3
H3K4ac
log2 FE
−4
−2
0
2
4
6
8

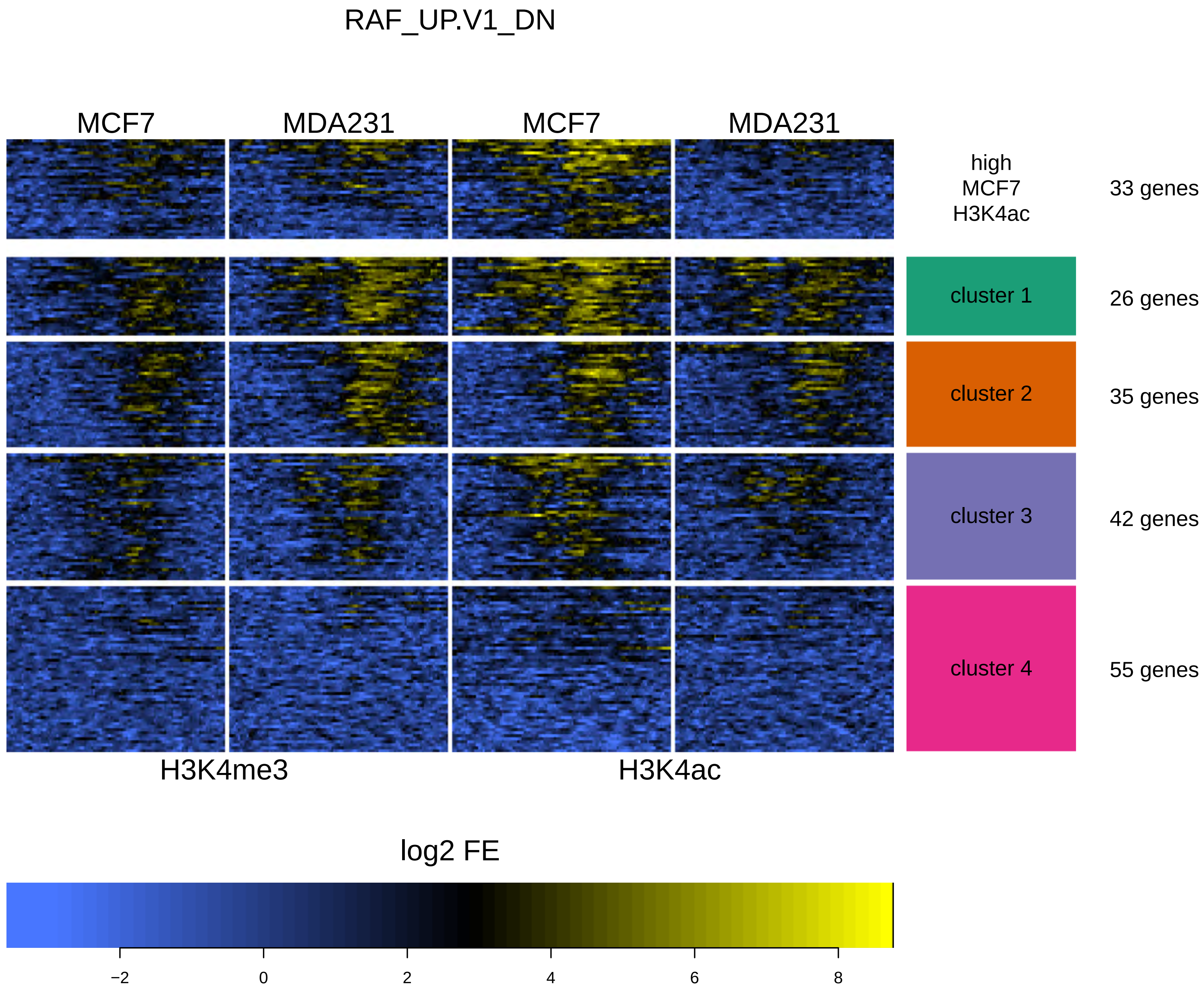
RAF_UP.V1_DN
MCF7
MDA231
MCF7
MDA231
high
MCF7
H3K4ac
33 genes
cluster 1
26 genes
cluster 2
35 genes
cluster 3
42 genes
cluster 4
55 genes
H3K4me3
H3K4ac
log2 FE
−2
0
2
4
6
8

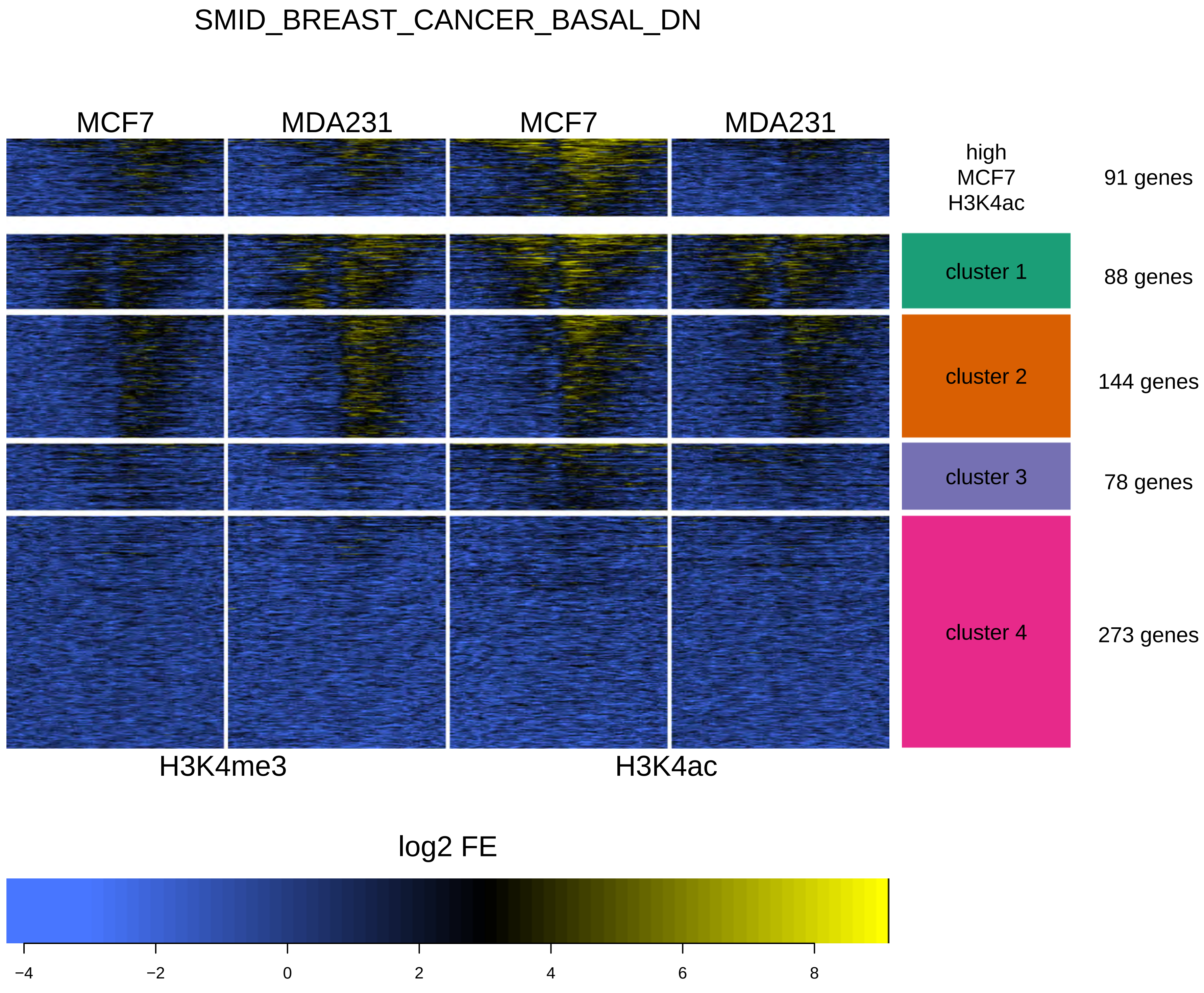
SMID_BREAST_CANCER_BASAL_DN
MCF7
MDA231
MCF7
MDA231
high
MCF7
H3K4ac
91 genes
cluster 1
88 genes
cluster 2
144 genes
cluster 3
78 genes
cluster 4
273 genes
H3K4me3
H3K4ac
log2 FE
−4
−2
0
2
4
6
8

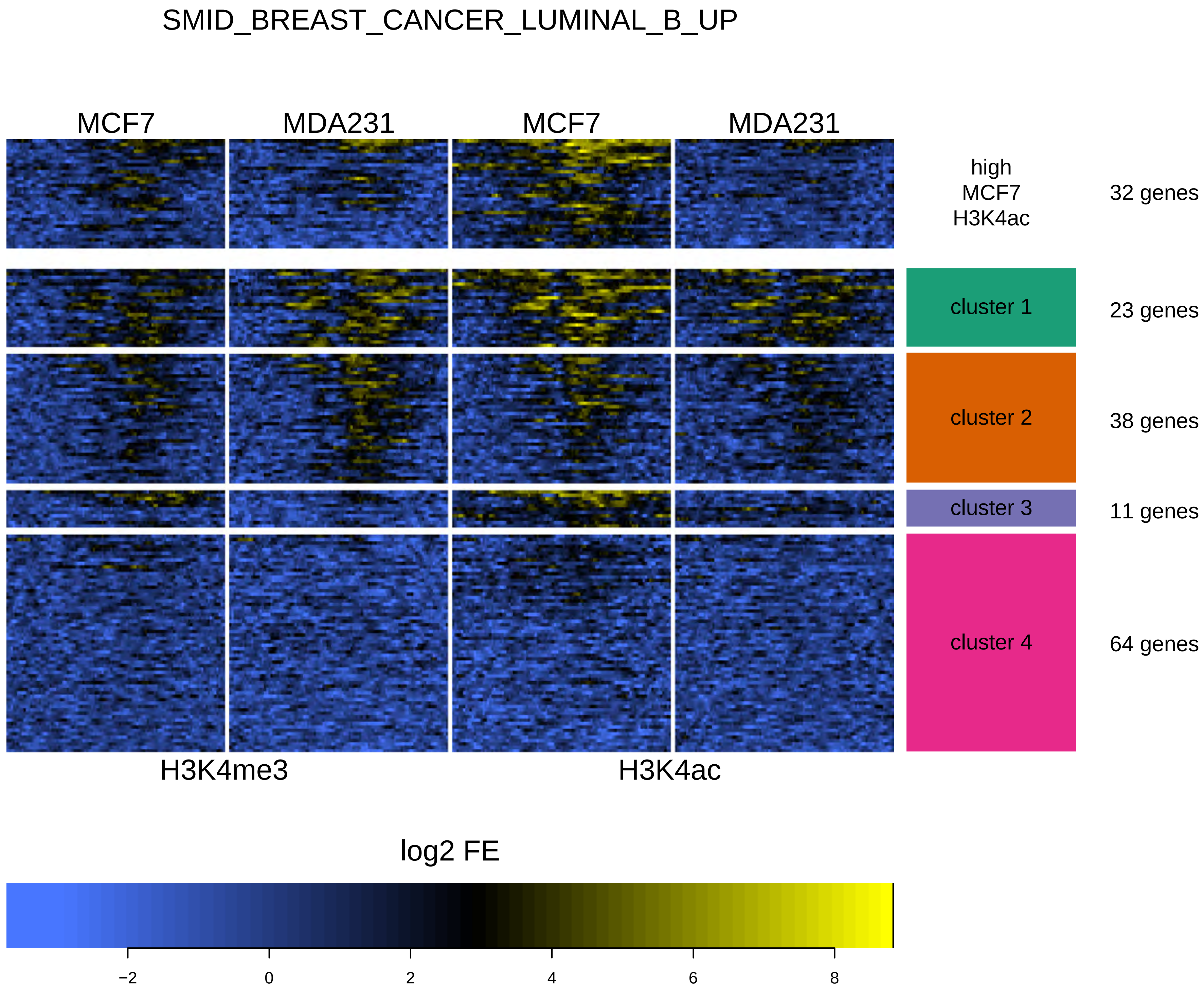
SMID_BREAST_CANCER_LUMINAL_B_UP
MCF7
MDA231
MCF7
MDA231
high
MCF7
H3K4ac
32 genes
cluster 1
23 genes
cluster 2
38 genes
cluster 3
11 genes
cluster 4
64 genes
H3K4me3
H3K4ac
log2 FE
−2
0
2
4
6
8

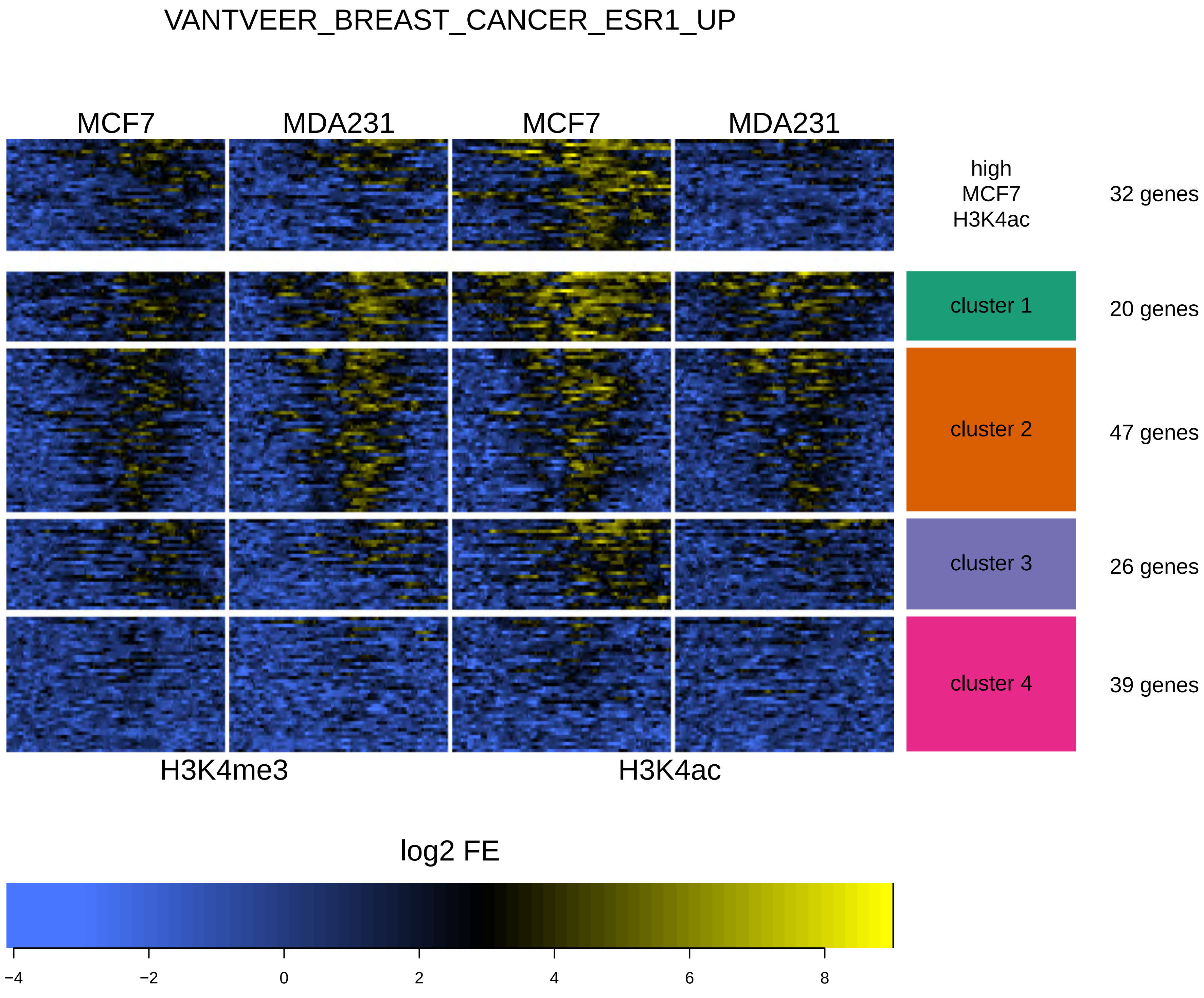

VANTVEER_BREAST_CANCER_ESR1_UP
MCF7
MDA231
MCF7
MDA231
high
MCF7
H3K4ac
32 genes
cluster 1
20 genes
cluster 2
47 genes
cluster 3
26 genes
cluster 4
39 genes
H3K4me3
H3K4ac
log2 FE
−4
−2
0
2
4
6
8

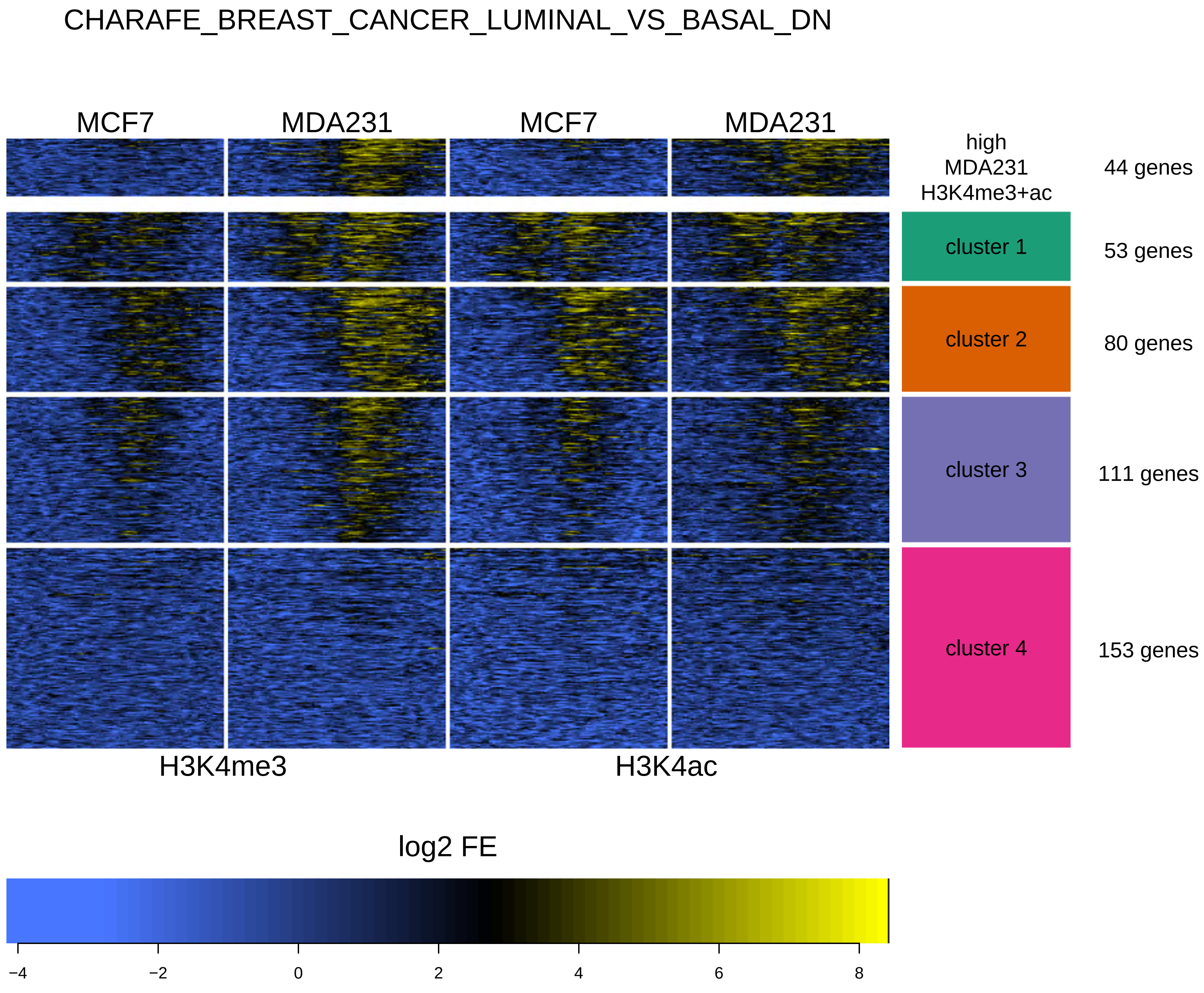
CHARAFE_BREAST_CANCER_LUMINAL_VS_BASAL_DN
MCF7
MDA231
MCF7
MDA231
high
MDA231
H3K4me3+ac
44 genes
cluster 1
53 genes
cluster 2
80 genes
cluster 3
111 genes
cluster 4
153 genes
H3K4me3
H3K4ac
log2 FE
−4
−2
0
2
4
6
8

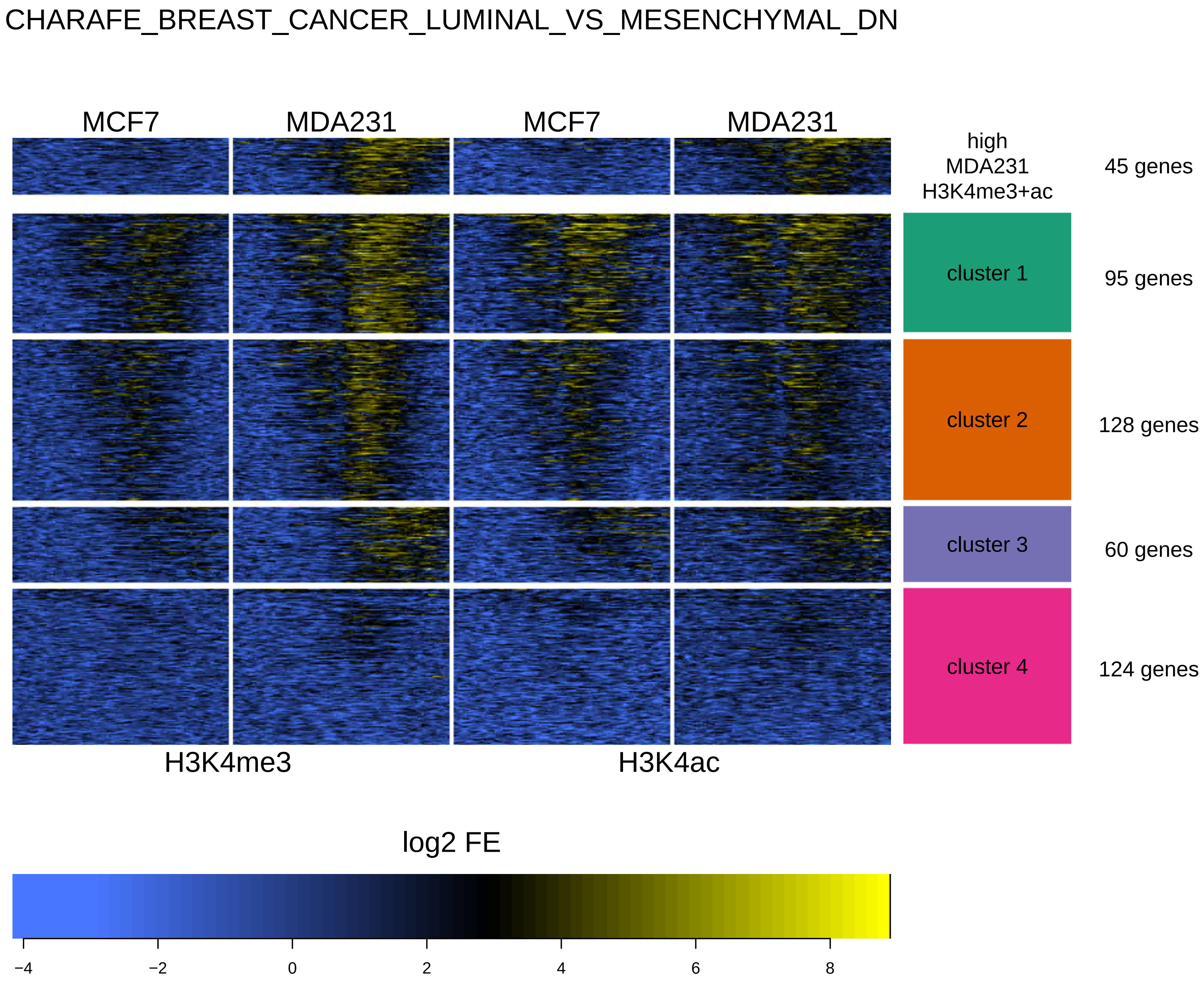
CHARAFE_BREAST_CANCER_LUMINAL_VS_MESENCHYMAL_DN
MCF7
MDA231
MCF7
MDA231
high MDA231 H3K4me3+ac
45 genes
cluster 1
95 genes
cluster 2
128 genes
cluster 3
60 genes
cluster 4
124 genes
H3K4me3
H3K4ac
log2 FE
−4
−2
0
2
4
6
8

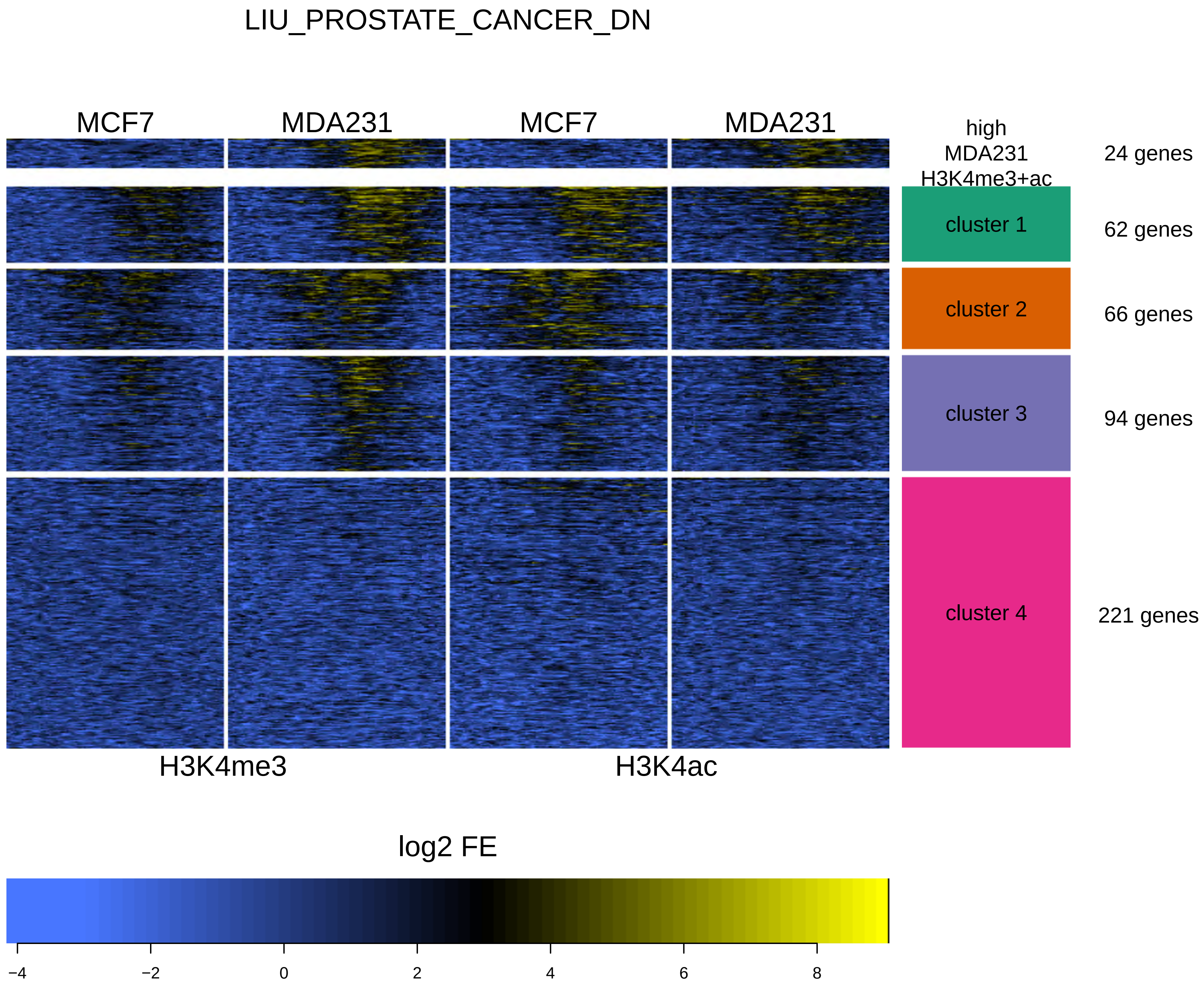

LIU_PROSTATE_CANCER_DN
MCF7
MDA231
MCF7
MDA231
high
MDA231
H3K4me3+ac
24 genes
cluster 1
62 genes
cluster 2
66 genes
cluster 3
94 genes
cluster 4
221 genes
H3K4me3
H3K4ac
log2 FE
−4
−2
0
2
4
6
8

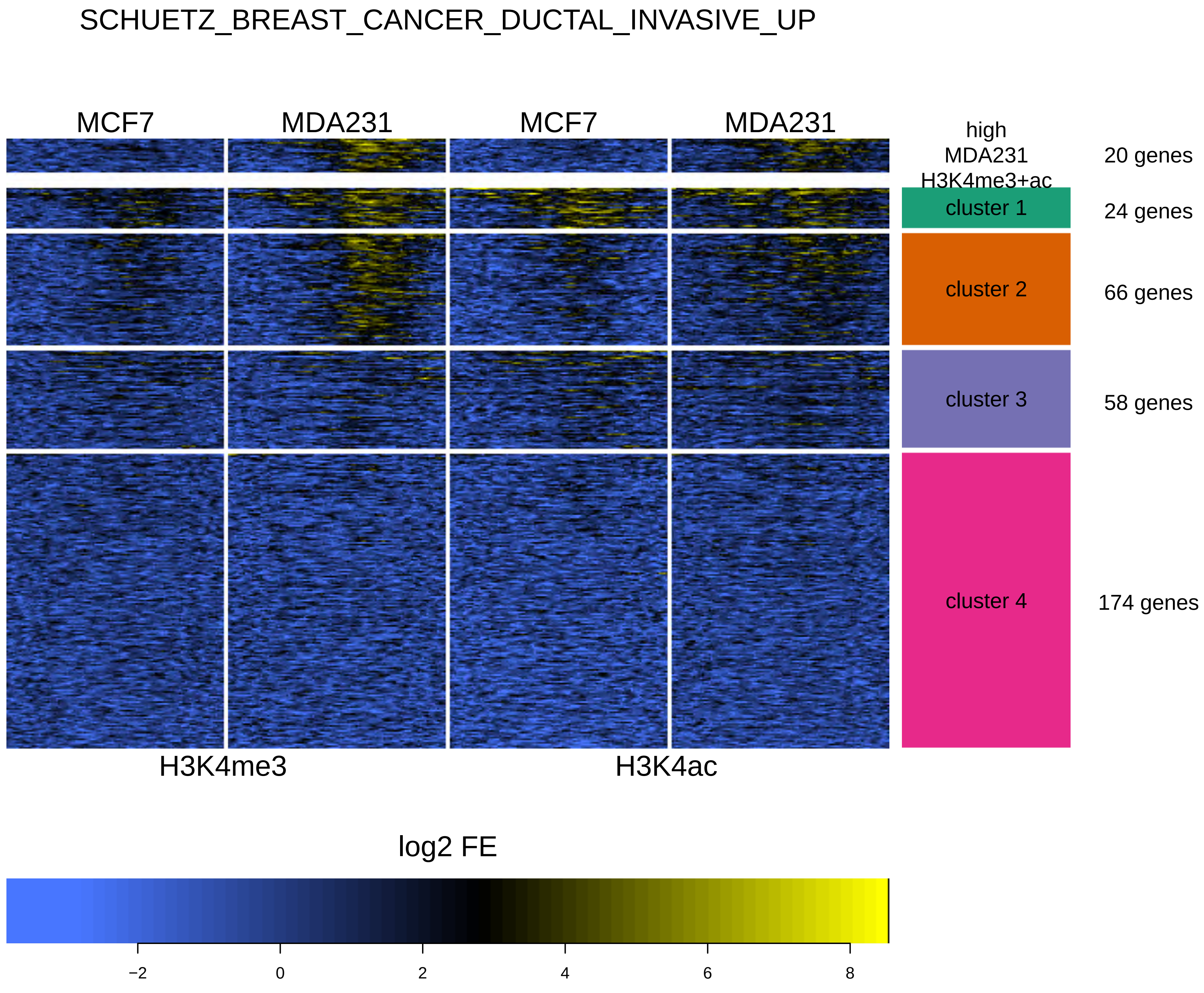
SCHUETZ_BREAST_CANCER_DUCTAL_INVASIVE_UP
MCF7
MDA231
MCF7
MDA231
high
MDA231
H3K4me3+ac
20 genes
cluster 1
24 genes
cluster 2
66 genes
cluster 3
58 genes
cluster 4
174 genes
H3K4me3
H3K4ac
log2 FE
−2
0
2
4
6
8

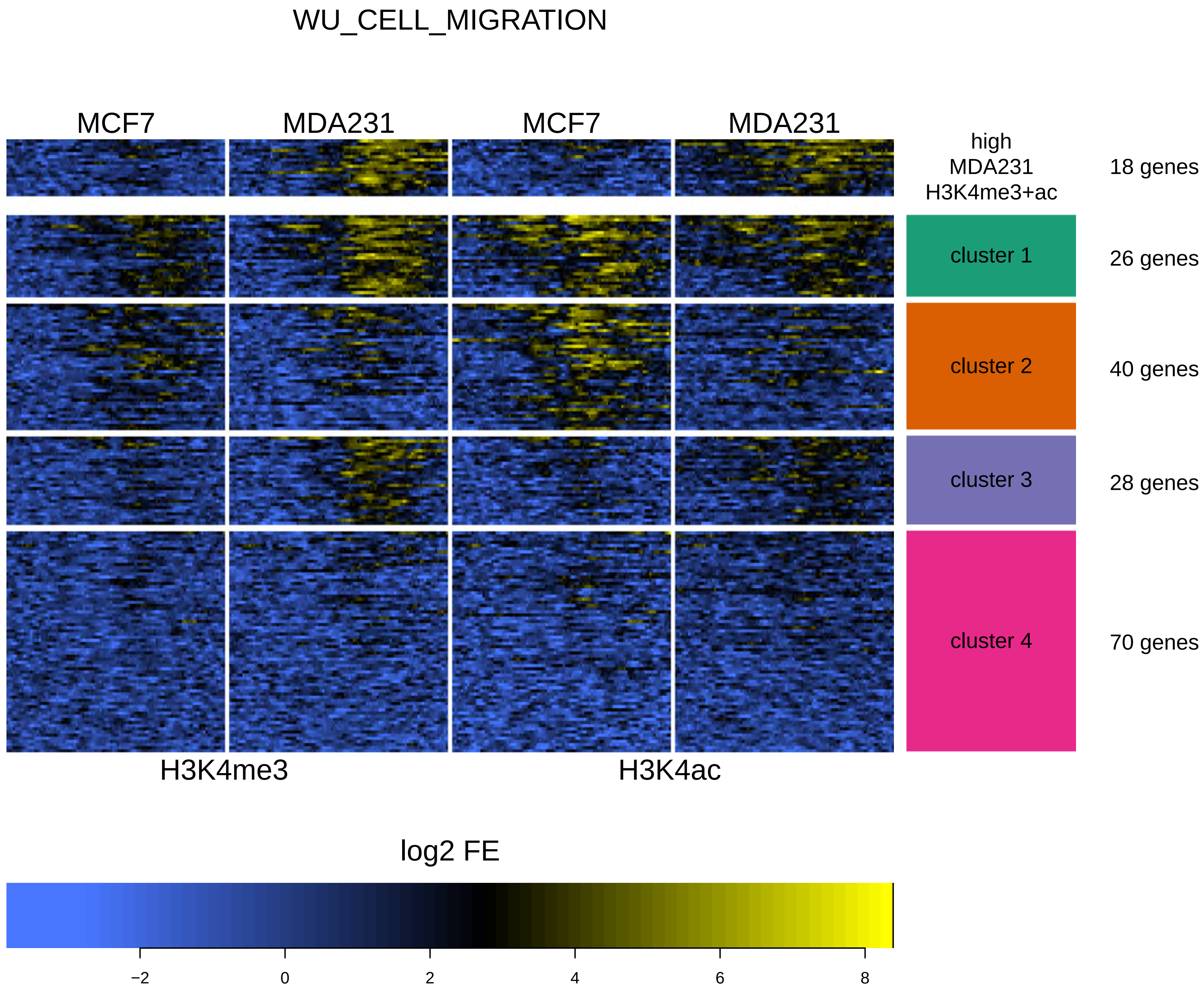
WU_CELL_MIGRATION
MCF7
MDA231
MCF7
MDA231
high
MDA231
H3K4me3+ac
18 genes
cluster 1
26 genes
cluster 2
40 genes
cluster 3
28 genes
cluster 4
70 genes
H3K4me3
H3K4ac
log2 FE
−2
0
2
4
6
8

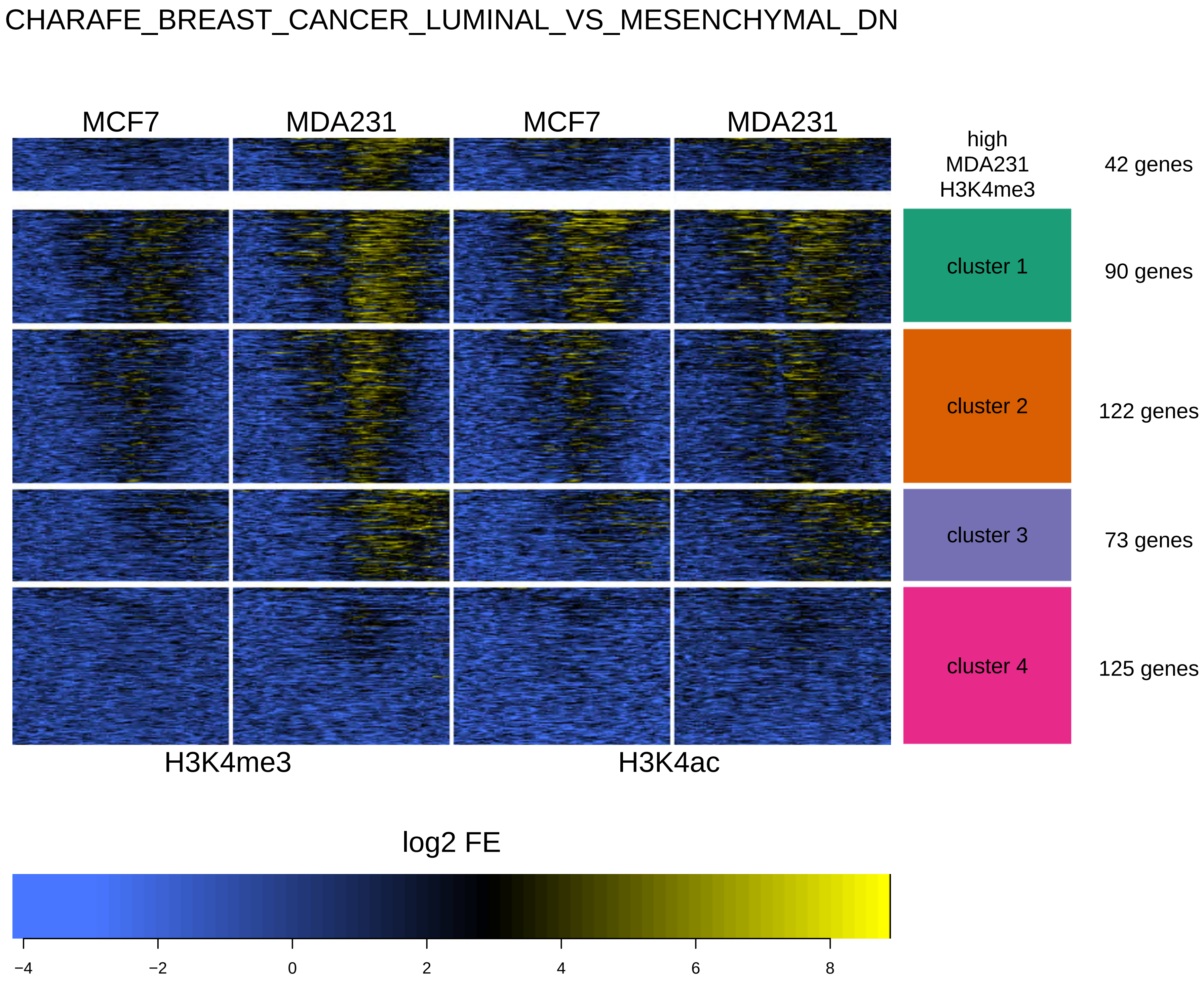
CHARAFE_BREAST_CANCER_LUMINAL_VS_MESENCHYMAL_DN
MCF7
MDA231
MCF7
MDA231
high MDA231 H3K4me3
42 genes
cluster 1
90 genes
cluster 2
122 genes
cluster 3
73 genes
cluster 4
125 genes
H3K4me3
H3K4ac
log2 FE
−4
−2
0
2
4
6
8
